# Microglia and complement mediate early corticostriatal synapse loss and cognitive dysfunction in Huntington’s disease

**DOI:** 10.1038/s41591-023-02566-3

**Published:** 2023-10-09

**Authors:** Daniel K. Wilton, Kevin Mastro, Molly D. Heller, Frederick W. Gergits, Carly Rose Willing, Jaclyn B. Fahey, Arnaud Frouin, Anthony Daggett, Xiaofeng Gu, Yejin A. Kim, Richard L. M. Faull, Suman Jayadev, Ted Yednock, X. William Yang, Beth Stevens

**Affiliations:** 1 F. M. Kirby Neurobiology Center, Department of Neurology, Boston Children’s Hospital, Harvard Medical School, Boston, MA US; 2 Center for Neurobehavioral Genetics, Jane and Terry Semel Institute for Neuroscience and Human Behavior, Department of Psychiatry and Biobehavioral Sciences, David Geffen School of Medicine at University of California, Los Angeles, CA USA; 3 Department of Anatomy with Radiology, Faculty of Medical and Health Sciences, University of Auckland, Auckland, New Zealand; 4 Department of Neurology, University of Washington, Seattle, WA USA; 5 Division of Medical Genetics, Department of Medicine, University of Washington, Seattle, WA USA; 6 Annexon Biosciences, South San Francisco, CA USA; 7 Stanley Center, Broad Institute, Cambridge, MA USA; 8 Howard Hughes Medical Institute, Boston Children’s Hospital, Harvard Medical School, Boston, MA USA

**Keywords:** Microglia, Huntington's disease

## Abstract

Huntington’s disease (HD) is a devastating monogenic neurodegenerative disease characterized by early, selective pathology in the basal ganglia despite the ubiquitous expression of mutant huntingtin. The molecular mechanisms underlying this region-specific neuronal degeneration and how these relate to the development of early cognitive phenotypes are poorly understood. Here we show that there is selective loss of synaptic connections between the cortex and striatum in postmortem tissue from patients with HD that is associated with the increased activation and localization of complement proteins, innate immune molecules, to these synaptic elements. We also found that levels of these secreted innate immune molecules are elevated in the cerebrospinal fluid of premanifest HD patients and correlate with established measures of disease burden.

In preclinical genetic models of HD, we show that complement proteins mediate the selective elimination of corticostriatal synapses at an early stage in disease pathogenesis, marking them for removal by microglia, the brain’s resident macrophage population. This process requires mutant huntingtin to be expressed in both cortical and striatal neurons. Inhibition of this complement-dependent elimination mechanism through administration of a therapeutically relevant C1q function-blocking antibody or genetic ablation of a complement receptor on microglia prevented synapse loss, increased excitatory input to the striatum and rescued the early development of visual discrimination learning and cognitive flexibility deficits in these models. Together, our findings implicate microglia and the complement cascade in the selective, early degeneration of corticostriatal synapses and the development of cognitive deficits in presymptomatic HD; they also provide new preclinical data to support complement as a therapeutic target for early intervention.

## Abstract (web summary)

Microglia mediate the early and selective degeneration of corticostriatal synapses and the development of cognitive deficits in presymptomatic Huntington’s disease via the complement cascade.

## Main

Huntington’s disease (HD) is the most common autosomal dominant neurodegenerative disease. It is characterized by progressive motor, cognitive and psychiatric symptoms, with onset of the manifest phase typically occuring around 45 years of age^1^. Currently, there are no therapies that modify disease onset or progression.

HD is caused by a CAG repeat expansion mutation in the *HTT* (huntingtin) gene encoding an expanded polyglutamine (PolyQ) tract in the mutant huntingtin protein^2,3^. Based on this genetic finding, multiple transgenic animal models have been generated to interrogate the underlying biology of the disease^4,5^; however, the molecular mechanisms that drive early and selective degeneration of basal ganglia circuits and how these relate to cognitive phenotypes remain poorly understood^6–23^. The corticostriatal pathway, which connects intratelencephalic and pyramidal tract neurons in the cortex with medium spiny neurons (MSNs) and cholinergic interneurons (ChIs) in the striatum, is affected at very early stages of disease progression^19,24^. Electrophysiological recordings in mouse models and structural and functional imaging of patients with HD in the premanifest stage of the disease reveal altered white matter structure and functional connectivity in this pathway, which correlates with a more rapid cognitive decline^18,25–29^. Reductions in synaptic marker levels suggest that corticostriatal synapses are lost, but it is unknown whether this loss occurs before onset of motor and cognitive deficits or why corticostriatal synapses are selectively vulnerable^22^.

Studies in mouse models of Alzheimer’s disease and frontotemporal dementia have demonstrated a link between synaptic loss and components of the classical complement cascade, a group of secreted ‘eat me’ signals that mediate recognition and engulfment of synaptic elements by microglia during development and in disease contexts^30–36^. Although this mechanism of synaptic elimination has never been explored in HD, transcriptional profiling, brain imaging and analysis of patient-derived serum and cerebrospinal fluid (CSF) have indicated an altered neuro-immune state in premanifest HD patients^37–44^. In mouse models, microglia have also been found to display changes in morphology, deficits in motility and an altered transcriptional profile during the symptomatic phase of the disease^45–53^. Separately, transcriptomic studies have identified increased expression of complement proteins and their regulators in the basal ganglion of postmortem tissue from patients with HD^54–57^, suggesting that complement proteins and microglia are dysregulated. However, neither complement nor microglia has been studied at early stages of the disease, and it is unknown whether they contribute to synapse loss or the development of early cognitive deficits in HD.

By integrating findings from postmortem HD brain samples and two preclinical HD mouse models, we provide evidence that microglia and complement coordinate to selectively target corticostriatal synapses for early elimination in the dorsal striatum—a process initiated only when mutant HTT (mHTT) is expressed in both cortical and striatal neurons. Inhibition of synaptic elimination through administration of a therapeutic C1q function-blocking antibody (ANX-M1, Annexon Biosciences) or genetic ablation of microglial complement receptor 3 (CR3/ITGAM) reduces loss of corticostriatal synapses and improves visual discrimination learning and cognitive flexibility impairments at early stages of disease progression in HD models. We further show that aspects of this pathological synapse elimination mechanism may be operating in premanifest HD patients, as complement protein levels in the CSF of patients with HD correlate with an established predictor of both pathological severity and disease onset.

## Results

### Selective loss of corticostriatal synapses in patients with HD is associated with complement activation and changes in microglia

**Fig. 1:**
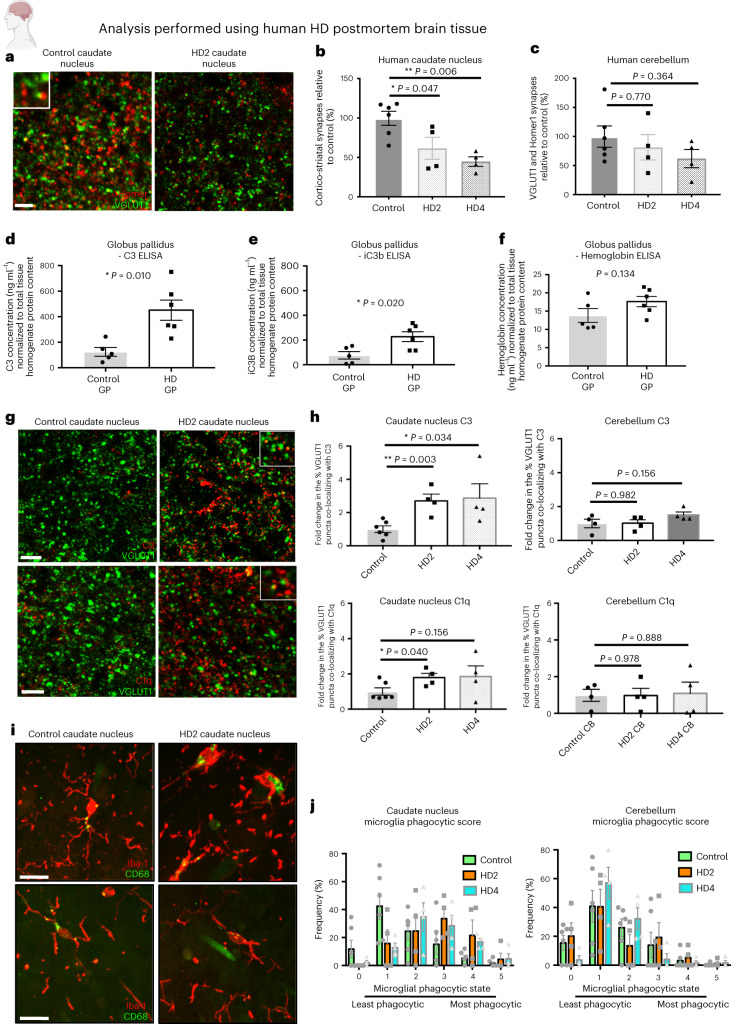
Loss of corticostriatal synapses, increased activation and association of complement proteins with synaptic elements and adoption of a more phagocytic microglial state are evident in postmortem brain tissue from patients with HD. **a**, Representative confocal images showing staining for corticostriatal specific presynaptic marker VGLUT1 and postsynaptic density protein Homer1 in the postmortem caudate nucleus of a control individual and a Vonsattel grade 2 patient with HD (Supplementary Table 2). Scale bar, 5 μm. **b**, Quantification of corticostriatal synapses (co-localized VGLUT1 and Homer1 puncta) in the caudate nucleus of control, Vonsattel grade 2 and Vonsattel grade 4 HD tissue, *n* = 6 control, *n* = 4 HD with Vonsattel 2 tissue grade and 4 HD with Vonsattel 4 tissue grade. One-way ANOVA *P* = 0.0058; Tukey’s multiple comparisons test, control versus HD2 *P* = 0.0474; control versus HD4 *P* = 0.0061; HD2 versus HD4 *P* = 0.537. **c**, The same analysis carried out in the molecular layer of the folia of the cerebellum (an area thought to be less impacted by disease) showed no change, *n* = 6 control individuals, 4 HD Vonsattel grade 2 and 4 HD Vonsattel grade 4. One-way ANOVA, *P* = 0.393; Tukey’s multiple comparisons test, control versus HD2 *P* = 0.770; control versus HD4 *P* = 0.365; HD2 versus HD4 *P* = 0.791. **d**, ELISA measurements of complement component C3 in extracts from the globus pallidus (GP) of postmortem tissue from manifest HD patients and control individuals (Supplementary Table 2) after normalization for total tissue homogenate protein content, *n* = 5 control GP and 6 HD GP. ANCOVA controlling for the effect of age *F*_2,10_ = 8.74, *P* = 0.010. **e**, ELISA measurements of complement component iC3b in the same extracts as **d**. ANCOVA controlling for the effect of age *F*_2,10_ = 6.71, *P* = 0.020. **f**, ELISA hemoglobin measurements in the same extracts as **d**. Unpaired two-tailed *t*-test *P* = 0.134. **g**, Representative confocal images showing staining for VGLUT1 together with C3 or C1q in the postmortem caudate nucleus of a control individual and a patient with HD who has been assessed to be Vonsattel grade 2. Scale bar, 5 μm. Insets show examples of co-localization of both complement proteins with presynaptic marker VGLUT1. **h**, Quantification of the percentage of VGLUT1^+^ glutamatergic synapses associating with C3 and C1q puncta in the caudate nucleus and cerebellum of postmortem tissue from patients with HD assessed to be either Vonsattel grade 2 or Vonsattel grade 4 relative to that seen in tissue from control individuals. For C3 in the caudate nucleus samples, *n* = 6 control individuals, *n* = 4 HD individuals with Vonsattel tissue grade 2 and *n* = 4 HD individuals with Vonsattel grade 4. One-way ANOVA, *P* = 0.029. Unpaired two-tailed *t*-test control versus HD2 *P* = 0.003; control versus HD4 *P* = 0.034. For C1Q in the caudate nucleus samples, *n* = 6 control individuals, *n* = 4 HD individuals with Vonsattel tissue grade 2 and *n* = 4 HD individuals with Vonsattel tissue grade 4. One-way ANOVA, *P* = 0.181. Unpaired two-tailed *t*-test, control versus HD2 *P* = 0.040, control versus HD4 *P* = 0.156. For C3 and C1Q in the cerebellum samples, *n* = 4 control individuals, *n* = 4 HD individuals with Vonsattel tissue grade 2 and *n* = 4 HD individuals with Vonsattel tissue grade 4. For C3, one-way ANOVA, *P* = 0.215. Unpaired two-tailed *t*-test, control versus HD2 *P* = 0.982; control versus HD4 *P* = 0.156. For C1q, one-way ANOVA, *P* = 0.981. Unpaired two-tailed *t*-test, control versus HD2 *P* = 0.978; control versus HD4 *P* = 0.888. **i**, Representative confocal images showing staining for Iba-1 and CD68 in the caudate nucleus and cerebellum of postmortem tissue from a control individual and a patient with HD (Vonsattel grade 2). Scale bar, 20 μm. **j**, Quantification of microglia phagocytic state based on changes in morphology and CD68 levels. For caudate nucleus samples, *n* = 6 control individuals, *n* = 4 HD individuals with Vonsattel tissue grade 2 and *n* = 4 HD individuals with Vonsattel tissue grade 4. Multiple unpaired two-tailed *t*-tests, control versus HD2; score 0 *P* = 0.253, score 1 *P* = 0.01, score 2 *P* = 0.984, score 3 *P* = 0.077, score 4 iP = 0.116 and score 5 *P* = 0.776. Control versus HD4; score 0 *P* = 0.255, score 1 *P* = 0.039, score 2 *P* = 0.382, score 3 *P* = 0.256, score 4 *P* = 0.006 and score 5 *P* = 0.309. For cerebellum samples, *n* = 6 control individuals, *n* = 4 HD individuals with Vonsattel tissue grade 2 and *n* = 4 HD individuals with Vonsattel tissue grade 4. Multiple unpaired two-tailed *t*-tests, control versus HD2; score 0 *P* = 0.635, score 1 *P* = 0.988, score 2 *P* = 0.243, score 3 *P* = 0.706, score 4 *P* = 0.610 and score 5 *P* = 0.477. Control versus HD4; score 0 *P* = 0.120, score 1 *P* = 0.348, score 2 *P* = 0.538, score 3 *P* = 0.415, score 4 *P* = 0.609 and score 5 *P* = 0.418. All error bars represent s.e.m. *P < 0.05, **P < 0.01 and ***P < 0.001. Source data

**Extended Data Fig. 1:**
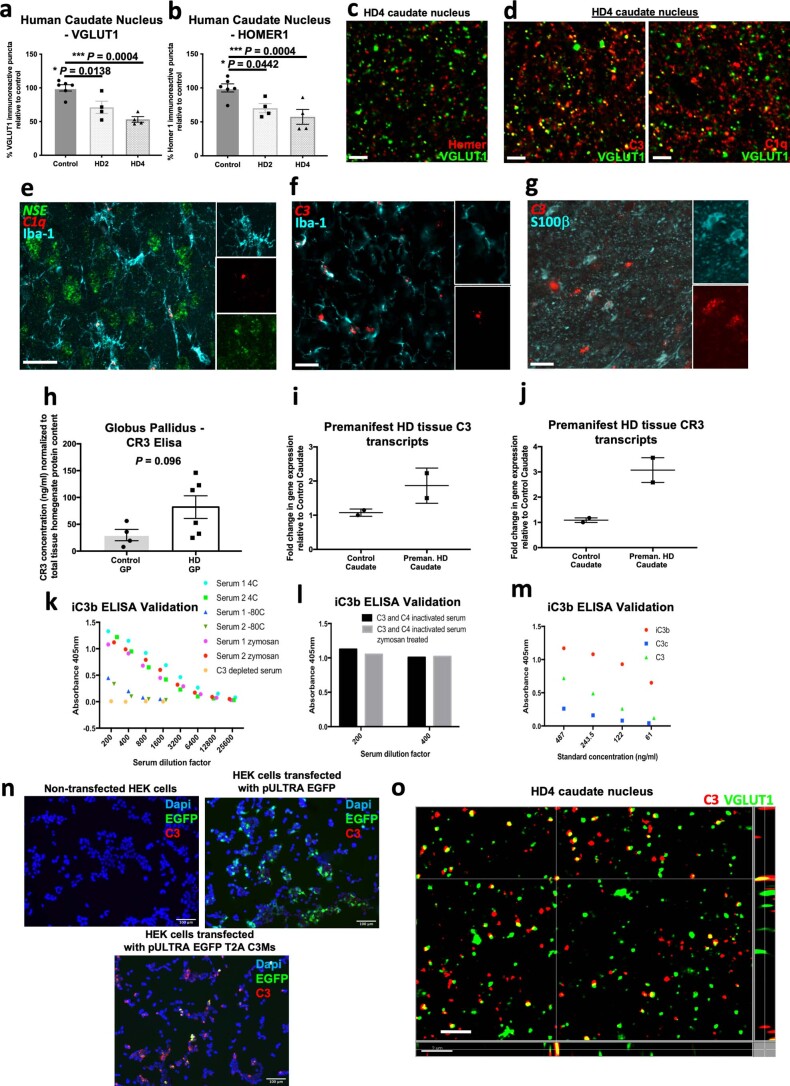
Loss of specific synaptic populations, activation and association of complement proteins with synaptic elements and adoption of a more phagocytic microglial state are evident in postmortem brain tissue from HD patients. **(a)** Bar chart showing quantification of VGLUT1 immunoreactive puncta in the caudate nucleus of control, Vonsattel grade 2 HD and Vonsattel grade 4 HD tissue, n = 6 control, n = 4 HD with Vonsattel 2 tissue grade and 4 HD with Vonsattel 4 tissue grade. One way anova p = 0.0005; Tukey’s multiple comparisons test, control vs HD2 p = 0.0138; control vs HD4 p = 0.0004; HD2 vs HD4 p = 0.172. **(b)** Bar chart showing quantification of HOMER1 immunoreactive puncta in the caudate nucleus of control, Vonsattel grade 2 HD and Vonsattel grade 4 HD tissue, n = 6 control, n = 4 HD with Vonsattel 2 tissue grade and 4 HD with Vonsattel 4 tissue grade. One way anova p = 0.0054; Tukey’s multiple comparisons test, control vs HD2 p = 0.0442; control vs HD4 p = 0.0058; HD2 vs HD4 p = 0.541. **(c)** Representative confocal image showing staining for corticostriatal synaptic markers in the caudate nucleus of an HD patient, who has been assessed to be Vonsattel grade 4. Scale bar = 5 μm. This experiment was repeated 3 times. **(d)** Left panel is a representative confocal image showing staining for C1Q and VGLUT1 in the caudate nucleus of an HD patient, who has been assessed to be Vonsattel grade 4. Scale bar = 5 μm. Right panel is a representative confocal image showing staining for C3 and VGLUT1 in the caudate nucleus of an HD patient, who has been assessed to be Vonsattel grade 4. Scale bar = 5 μm. This experiment was repeated 3 times. **(e)** Representative confocal image of in situ staining for *C1Q* and *NSE* alongside IHC for microglial marker IBA1 in the caudate nucleus of postmortem tissue from an HD patient (Vonsattel grade 2). Scale bar = 20 μm. **(f)** Representative confocal image of in situ staining for *C3* alongside IHC for microglial marker IBA1 in the caudate nucleus of postmortem tissue from an HD patient (Vonsattel grade 2). Scale bar = 20 μm. This experiment was repeated 3 times. **(g)** Representative confocal image of an in situ staining for *C3* alongside IHC for astrocytic marker S100β in the caudate nucleus of postmortem tissue from an HD patient (Vonsattel grade 2). Scale bar = 20 μm. This experiment was repeated 3 times. **(h)** Bar chart showing ELISA measurements of the concentration of complement receptor CR3 in proteins extracted from the globus pallidus (GP) of postmortem tissue from manifest HD patients and control (no documented evidence of neurodegenerative disease; see Methods and supplemental table 2) individuals after normalization for total tissue homogenate protein content, n = 4 control GP and 6 HD GP (there was not enough protein available from one of the control sample that had previously been employed in the C3, iC3b and hemoglobin ELISA’s (the results of which are depicted in Fig. 5a,b and c) and as such it was not tested here). Unpaired two-tailed t-test p = 0.096 **(i)** Dot plot showing the fold change in mRNA levels of complement component C3 in samples from the caudate nucleus of two premanifest HD patients relative to those seen in two clinically normal (see Methods and Supplemental table 2) individuals. **(j)** Dot plot showing the fold change in mRNA levels of complement receptor CR3 in samples from the caudate nucleus of two premanifest HD patients relative to those seen in two clinically normal (see Methods and Supplemental table 2) individuals. **(k)** Superimposed scatter blot showing the relative absorbance values of different serum samples employed in the iC3b ELISA. Note that in two independent serum samples, in which the complement pathway has been activated either by incubating the serum at 4 °C for 7 days or treating with 10 mg/ml of zymosan for 30 min at 37 °C, the absorbance values for the highest concentration of serum tested are approximately double that of the same samples left untreated and maintained at -80 °C. Thus demonstrating that this assay reflects complement cascade activation as would be predicted for an ELISA measuring levels of iC3b, a cleavage fragment of complement component C3 formed following cascade activation. **(l)** Bar graph showing the relative absorbance values of C3/C4 inactivated serum samples employed in the iC3b ELISA. Note that, unlike in **(k)** treatment of this serum with zymosan fails to increase levels of iC3b. Thus confirming the specificity of the ELISA by demonstrating that it reflects changes in a species that increases in response to complement cascade activation but is prevented from forming in the absence of functioning C3 and C4. **(m)** Superimposed scatter blot showing the relative absorbance values of different concentrations of complement component C3 standards purified from human serum using PEG precipitation and DEAE ion chromatography. Note that at all concentrations tested iC3b (generated by the cleavage of C3b with factor I in the presence of factor H) gave a higher absorbance value than uncleaved full length C3 or a subsequent cleavage component C3c (generated by treating iC3b with “trypsin like” proteases). Thus further demonstrating the specificity of this ELISA for iC3b versus the full-length protein or other cleavage components. **(n)** Representative images of non-transfected HEK 293 cells or those transfected with pULTRA EGFP or pULTRA EGFP T2A C3Ms stained with the same C3 antibody employed in the immunohistochemical analysis depicted in this figure and in Fig. 3, Extended Data Fig. 3, Fig. 5, Extended Data Fig. 8g, and Extended Data Fig. 4. Scale bar = 100 μm. **(o)** Orthogonal view of a representative structured illumination image showing C3 and VGLUT1 staining in the caudate nucleus of tissue from an HD patient (Vonsattel grade 4). Scale bar = 2 μm. For bar charts, bars depict the mean This experiment was repeated four times. All error bars represent SEM. Stars depict level of significance with *=p < 0.05, **p = <0.01 and ***p < 0.001. Source data

To test whether there is selective loss of corticostriatal synapses in patients with HD, we assessed glutamatergic excitatory synapses in postmortem tissue from the caudate nucleus (part of the striatum) and cerebellum of control individuals (no documented evidence of neurodegenerative disease) and patients with HD with different Vonsattel grades of striatal HD neuropathology^58–61^. Immunohistochemical (IHC) analysis of corticostriatal synapses, as denoted by co-localization of excitatory postsynaptic marker Homer1 and presynaptic corticostriatal marker VGLUT1, revealed a progressive and significant loss in the caudate of the HD tissue relative to that seen in tissue from control individuals (Fig. 1a,b). Conversely, we observed no difference in VGLUT1-positive glutamatergic synapses in the cerebellum, which is less affected in HD (Fig. 1c and Extended Data Fig. 1a–c).

To determine whether complement proteins could contribute to loss of corticostriatal synapses, we measured levels of C3 and its activated cleavage component iC3b (a cognate ligand for microglial CR3), which functions downstream in the complement pathway as an opsonin (marking substances for removal by phagocytic cells)^62,63^. Extracts from the globus pallidus, a component of the basal ganglion structure^64–66^, of patients with HD showed higher levels of both C3 and iC3b relative to those found in samples from age-matched controls despite both sets of extracts displaying similar levels of blood contamination (Fig. 1d–f and Extended Data Fig. 1k–m).

IHC analysis of postmortem tissue from the caudate nucleus and cerebellum of patients with HD (Vonsattel grade 2 and grade 4) and age-matched controls also revealed increased association of both complement component C1Q, the initiator of the classical complement cascade (expressed by microglia; Extended Data Fig. 1e) and C3 (expressed by both astrocytes and microglia; Extended Data Fig. 1f,g) with corticostriatal synapses in the caudate nucleus of HD brains. There was, however, no increased association of complement proteins with glutamatergic synapses in the cerebellum (a less disease-affected region), which correlates with the relative preservation of these structures in this region (Fig. 1g,h and Extended Data Fig. 1c,d,o).

Microglia in the HD tissue set displayed a region-specific shift toward a more phagocytic phenotype relative to that seen in age-matched controls, adopting a more amoeboid morphology and possessing higher levels of lysosomal marker CD68 (Fig. 1i,j). Consistent with previous transcriptomic studies, we also found that levels of the microglia-specific complement receptor 3 (CR3) were elevated in the HD globus pallidus; however, with the current sample size, this difference was not statistically significant (Extended Data Fig. 1h).

To test whether complement-mediated microglial synaptic elimination could occur in premanifest HD, we performed quantitative RT–PCR on RNA extracted from two rare samples of caudate nucleus from premanifest HD patients and found that levels of *C3* and *CR3* transcripts were elevated relative to controls (Extended Data Fig. 1i,j), consistent with a previous study that used unbiased transcriptomic profiling in the BA9 region of premanifest HD patients^55^. Together these results demonstrate that corticostriatal synapses are selectively and progressively lost in postmortem tissue from patients with HD and that this is accompanied by increased complement protein levels, activation and synaptic localization of complement proteins as well as phenotypic changes in microglia that suggest that complement-mediated synaptic elimination might be contributing to this synaptic pathology.

### Early and specific loss of corticostriatal synapses in HD mouse models

To further explore mechanisms underlying this synaptic pathology and determine whether corticostriatal synapses are selectively vulnerable early in disease, we quantified corticostriatal synapses together with the thalamostriatal synapse population (the other significant source of excitatory input to the striatum)^67–69^ in the dorsolateral striatum of zQ175 knock-in and BACHD human genomic transgenic mouse models of HD^70,71^. Both models develop a variety of electrophysiological abnormalities and have a similar timecourse of striatal and cortical atrophy, with zQ175 mice showing relatively mild motor deficits at 7 months of age^17,19,22,70–76^.

**Fig. 2:**
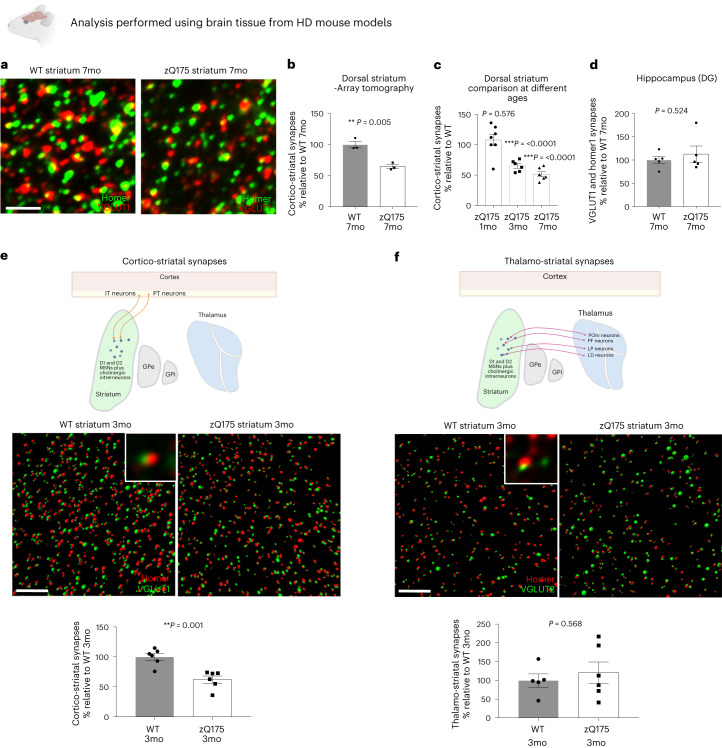
Early and selective loss of corticostriatal synapses in HD. **a**, Representative array tomography projections of dorsolateral striatum of 7-month-old zQ175 and WT littermates stained with antibodies to Homer1 and VGLUT1. Scale bar, 3 μm. **b**, Imaris and MATLAB quantification of synapse numbers in the array tomography projections, *n* = 3 WT mice and 3 zQ175. Unpaired two-tailed *t*-test, *P* = 0.0048. **c**, Quantification of corticostriatal synapses in the dorsolateral striatum of zQ175 mice at different ages expressed as a % of WT numbers at the same age, *n* = 7 WT mice and 7 zQ175 mice at 1 month of age; *n* = 6 WT and 6 zQ175 mice at 3 months of age; and *n* = 6 WT and 6 zQ175 mice at 7 months of age. Unpaired two-tailed *t*-test relative to WT at each age at 1 month of age *P* = 0.576, at 3 months of age *P* = 0.000064 and at 7 months of age *P* = 0.000007. **d**, Quantification of VGLUT1-labeled glutamatergic synapses in the molecular layer of the dentate gyrus of the hippocampus of 7-month-old zQ175 mice and WT littermates, *n* = 5 WT mice and 5 zQ175 mice. Unpaired two-tailed *t*-test, *P* = 0.524. **e**, SIM images show a significant reduction in the number of corticostriatal synapses in the dorsolateral striatum of 3-month-old zQ175 mice. Pictograms show synapses defined as presynaptic and postsynaptic spheres (rendered around immunofluorescent puncta) whose centers are less than 0.3 μm apart. Inset shows a representative example of co-localized presynaptic and postsynaptic puncta rendered through SIM imaging. Scale bar, 5 μm. Bar chart shows MATLAB quantification of corticostriatal synapses, *n* = 6 WT mice and 6 zQ175 mice. Unpaired two-tailed *t*-test, *P* = 0.001. **f**, SIMs showing no difference in the numbers of thalamostriatal synapses at 3 months of age as denoted by staining with the presynaptic marker VGLUT2. Scale bar, 5 μm. Bar chart shows quantification of these images, *n* = 5 WT and 6 zQ75 mice. Unpaired two-tailed *t*-test, *P* = 0.568. For bar charts, bars depict the mean. All error bars represent s.e.m. *P < 0.05, **P < 0.01 and ***P < 0.001. GPe, globus pallidus externa; GPi, globus pallidus internus; IT, intratelencephalic; LD, laterodorsal nucleus; LP, lateral posteria nucleus; mo, months old; POm, posterior medial nucleus; PF, parafascicular thalamic nucleus; PT, pyramidal tract; SIM, structured illumination microscopy. Source data

**Extended Data Fig. 2:**
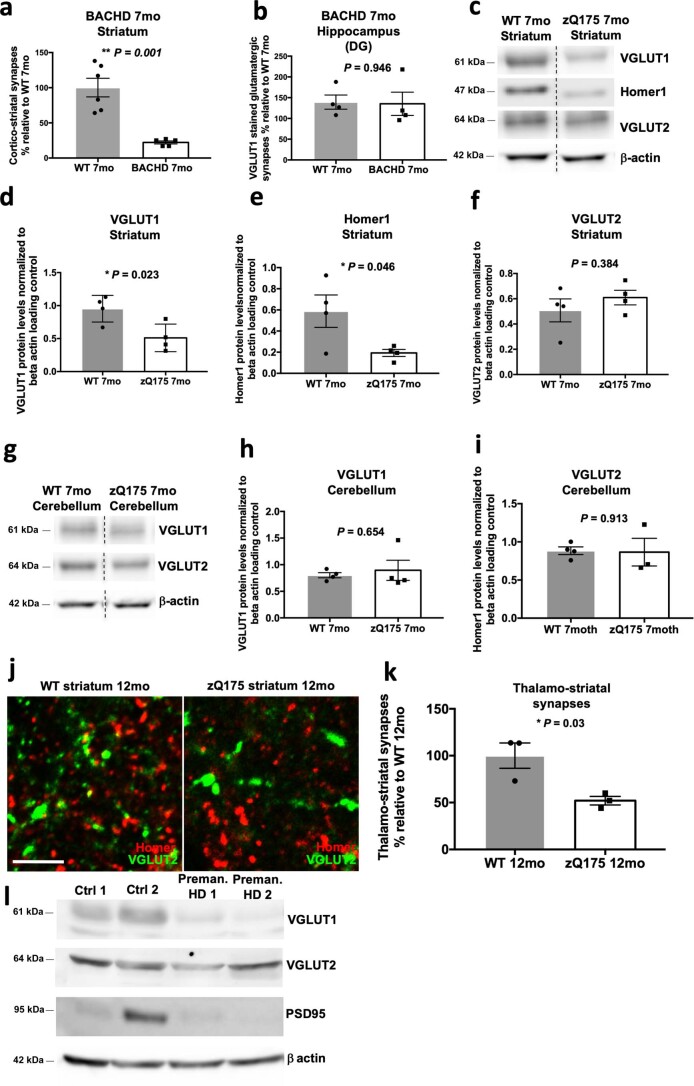
Early and selective loss of corticostriatal synapses in a mouse model of Huntington’s disease. **(a)** Bar chart shows quantification of corticostriatal synapses in the dorsolateral striatum of 7 mo BACHD mice and WT littermates, n = 6 WT 5 BACHD mice (3 F and 3 M for WT and 2 F and 3 M for BACHD). Unpaired two-tailed t-test p = 0.001. **(b)** Bar chart shows quantification of VGLUT1 stained glutamatergic synapses in the hippocampus (dentate gyrus) of 7 mo BACHD mice and WT littermates, n = 4 WT and 4 BACHD mice (2 F and 2 M for both genotypes). Unpaired two-tailed t-test p = 0.946. **(c)** Representative immunoblot showing that levels of pre and postsynaptic markers of the corticostriatal synapse, VGLUT1 and Homer1 respectively, are reduced in the striatum of 7 mo zQ175 mice relative to that seen in WT littermate controls but levels of the thalamostriatal synapse marker, VGLUT2, are not. Hashed lines are used to denote the fact that non-adjacent lanes from the same immunoblot are being depicted. Images showing the full lane chemiluminescent signal can be found in Source data 1. **(d,e,f)** Bar charts showing quantification of the relative protein levels of these synaptic markers in 7 mo zQ175 mice and WT littermates, n = 4 WT and 4 zQ175 mice (2 F and 2 M for both genotypes). Unpaired two-tailed t-tests, VGLUT1 p = 0.0227; Homer1 p = 0.0455; VGLUT2 p = 0.3836 **(g)** Representative immunoblot showing that protein levels of VGLUT1 and VGLUT2 are not changed in the cerebellum of 7 mo zQ175 mice relative to that seen in WT littermate controls. Hashed lines are used to denote the fact that non adjacent lanes from the same immunoblot are being depicted. Images showing the full lane chemiluminescent signal can be found in Source data 1. **(h,i)** Bar charts showing quantification of relative protein levels in the cerebellum of 7 mo zQ175 mice and WT littermates, for VGLUT1 n = 4 WT and 4 zQ175 mice (2 F and 2 M for both genotypes) for VGLUT2 n = 4 WT and 3 zQ175 mice (2 F and 2 M for WT and 2 F and 1 M for zQ175). Unpaired two-tailed t-tests, VGLUT1 p = 0.654; VGLUT2 p = 0.913. **(j)** Representative confocal images of VGLUT2 and Homer1 staining in the dorsolateral striatum of 12 mo zQ175 mice and WT littermates. Scale bar = 5 μm. **(k)** Bar chart shows quantification of colocalized VGLUT2 and Homer1 puncta denoting thalamostriatal synapses in these mice, n = 3 WT and 3 zQ175 mice (2 F and 1 M for both genotypes). Unpaired two-tailed t-test, p = 0.03 **(l)** Immunoblot of protein samples from the caudate nucleus of two premanifest HD patients and two clinically normal individuals stained with antibodies to VGLUT1, VGLUT2, PSD95 and β actin. Note the reduction in VGLUT1 levels (a marker of the corticostriatal synapse) and decrease in PSD-95 levels (a marker of the postsynaptic density) but no change in VGLUT2 levels (a marker of the thalamostriatal synapse), n = 2 caudate samples from control (clinically normal; see Methods and supplemental table 2) individuals and 2 caudate samples from individuals with premanifest HD. Images showing the full lane chemiluminescent signal can be found in source data 2. For bar charts, bars depict the mean. All error bars represent SEM. Stars depict level of significance with *=p < 0.05, **p = <0.01 and ***p < 0.001. Source data

Consistent with previous functional studies that suggested a reduction of glutamatergic inputs onto MSNs, we found ~50% fewer corticostriatal synapses in the dorsolateral striatum of 7-month-old zQ175 mice^72,74,77,78^ (Fig. 2a–c). Interestingly, this loss was not observed in the dentate gyrus of the hippocampus, a less disease-affected brain region (Fig. 2d), and was replicated in the BACHD model (Extended Data Fig. 2a,b). Concordantly, immunoblot analysis found reduced levels of synaptic markers in the striatum but not in the less disease-affected cerebellum in 7-month-old zQ175 mice (Extended Data Fig. 2c–i).

To test whether synapse loss occurs before onset of motor and cognitive deficits, we repeated the analysis in 3-month-old zQ175 mice and still saw a significant reduction of corticostriatal synapses (Fig. 2c,e). Notably, this did not appear to result from a developmental failure in synapse formation as, consistent with previous findings, no difference could be detected at 1 month of age in these mice (Fig. 2c)^79^.

The striatum receives excitatory inputs from both the cortex and thalamus, which can be distinguished by staining for presynaptic vesicular proteins VGLUT1 and VGLUT2, respectively^67,68,80–84^. Using antibodies to these markers, we found that corticostriatal synapses, but not thalamostriatal synapses, were lost in 3-month-old zQ175 mice (Fig. 2e,f). Only when mice were old enough to display motor deficits were both synaptic populations reduced, in line with previous reports in other HD models^70,85–87^ (Extended Data Fig. 2j,k). One group reported fewer thalamostriatal synapses in a different region of the striatum on postnatal day 21 (P21) and P35, presumably reflecting a developmental failure of synapse formation, although they used a different combination of synaptic markers that have been shown to be dependent on synaptic maturity for their localization at synaptic contacts^79,88^.

Consistent with the selective vulnerability of corticostriatal synapses, immunoblot analysis of two rare samples of caudate nucleus from premanifest HD patients also showed reductions in levels of corticostriatal marker VGLUT1 and postsynaptic marker PSD95 but not thalamostriatal marker VGLUT2 (Extended Data Fig. 2l), further suggesting selective vulnerability of the corticostriatal connection in HD. Taken together, these results show that corticostriatal synapse loss is an early event in the pathogenesis of mouse models of HD, occurring before onset of motor and cognitive deficits.

### Complement components are upregulated and specifically localize to vulnerable corticostriatal synapses in HD mouse models

**Fig. 3:**
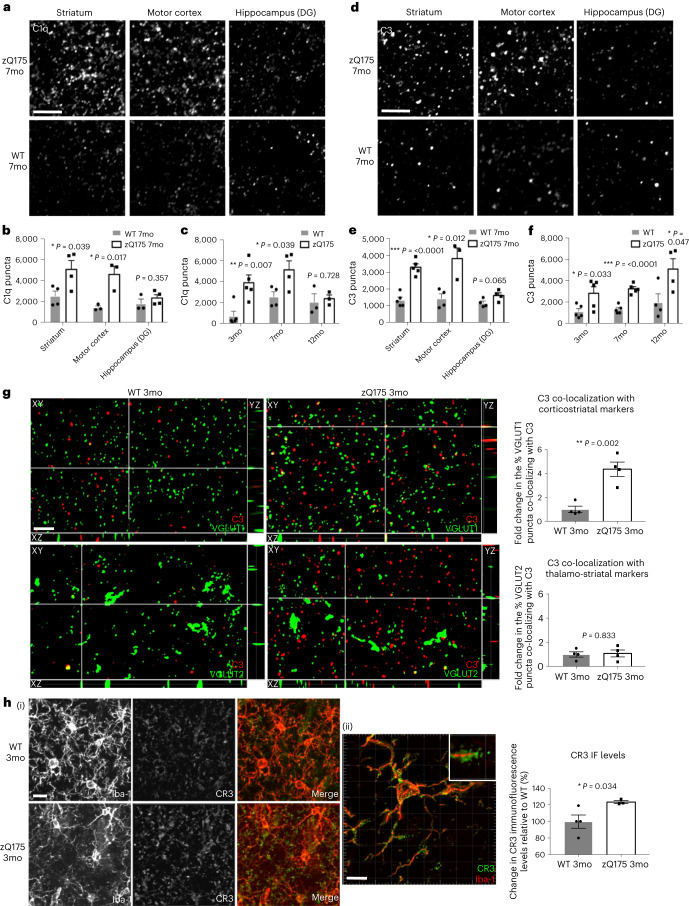
Complement proteins associate with specific synapses and microglia increase their expression of complement receptors in HD mouse models. **a**, Representative confocal images showing C1q staining in disease-affected (dorsolateral striatum and motor cortex) and less-affected regions (dentate gyrus (DG) of the hippocampus) of zQ175 mice and WT littermate controls. Scale bar, 5 μm. **b**, Quantification of C1q puncta at 7 months of age, striatum *n* = 4 WT and 4 zQ175 mice, motor cortex *n* = 3 WT and 3 zQ175 mice and hippocampus *n* = 3 WT and 4 zQ175 mice. Unpaired two-tailed *t*-test for comparisons of WT and zQ175 in each brain region, striatum *P* = 0.039, motor cortex *P* = 0.017 and hippocampus (DG) *P* = 0.357. **c**, Quantification of C1q puncta in the dorsolateral striatum at different ages in zQ175 mice and WT littermates—3 months of age *n* = 5 WT and 5 zQ175 mice; 7 months of age: same data represented in **b**; and 12 months of age *n* = 3 WT and 3 zQ175 mice. Unpaired two-tailed *t*-test for comparisons of WT and zQ175 at each age—3 months of age *P* = 0.007, 7 months of age *P* = 0.039 and 12 months of age *P* = 0.728. **d**, Representative confocal images showing C3 staining in disease-affected and less-affected regions of zQ175 mice and WT littermate controls. Scale bar, 5 μm. **e**, Quantification of C3 puncta in 7-month-old zQ175 mice and WT littermate controls—striatum *n* = 5 WT and 5 zQ175 mice, motor cortex *n* = 4 WT and 3 zQ175 mice and hippocampus (DG) *n* = 4 WT and 4 zQ175 mice. Unpaired two-tailed *t*-test for comparisons of WT and zQ175 in each brain region—striatum *P* = 0.000081, motor cortex *P* = 0.012 and hippocampus (DG) *P* = 0.065. **f**, Quantification of C3 puncta in the dorsolateral striatum at different ages in zQ175 mice and WT littermates—3 months of age *n* = 5 WT and 5 zQ175 mice; 7 months of age: same data represented in **e**; and 12 months of age *n* = 4 WT and 4 zQ175 mice. Unpaired two-tailed *t*-test for comparisons of WT and zQ175 at each age—3 months of age *P* = 0.033, 7 months of age *P* = 0.000081 and 12 months of age *P* = 0.047. **g**, Orthogonal views of SIM images showing C3 and VGLUT1 or C3 and VGLUT2 staining in 3-month-old zQ175 or WT dorsolateral striatum. Scale bar, 5 μm. Bar charts show MATLAB quantification of co-localized C3 and VGLUT puncta from Imaris-processed images, *n* = 4 WT and 4 zQ175 mice. Unpaired two-tailed *t*-tests for comparisons of WT and zQ175—VGLUT1 and C3 *P* = 0.002; VGLUT2 and C3 *P* = 0.833. **h** (I), Representative confocal images of CR3 and Iba1 staining in the dorsal striatum of 3-month-old zQ175 and WT mice. Although levels of microglia (and macrophage) marker Iba1 do not change (Extended Data Fig. 4g), CR3 levels are increased in the process tips of microglia from zQ175 mice. Scale bar, 10 μm. **h** (II), SIM image showing CR3 localized to the process tips of microglia in the dorsal striatum of zQ175 mice. Scale bar, 10 μm. Bar chart shows quantification of average CR3 fluorescence intensity in the dorsal striatum, *n* = 4 WT and 3 zQ175 mice. Unpaired two-tailed *t*-test, *P* = 0.034. For bar charts, bars depict the mean. All error bars represent s.e.m. *P < 0.05, **P < 0.01 and ***P < 0.001. mo, months old. IF, immunofluorescence. Source data

**Extended Data Fig. 3:**
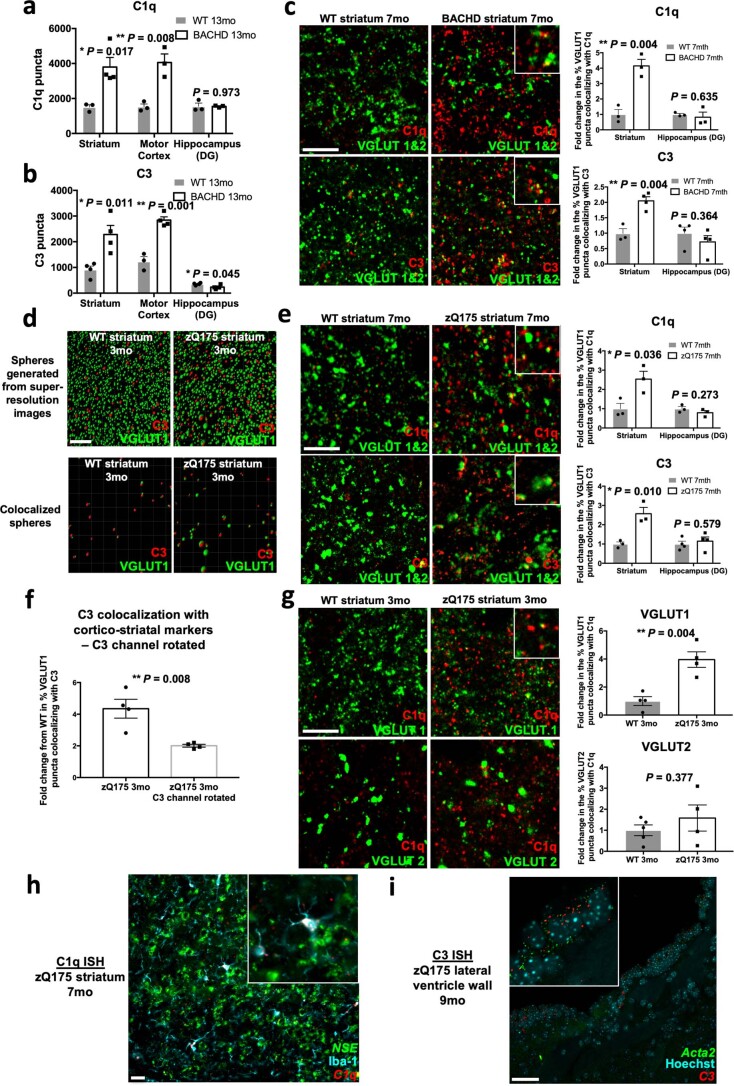
Complement proteins associate with specific synaptic connections in HD mouse models. **(a)** Bar charts showing quantification of C1q puncta in different brain regions of 13 mo BACHD mice and WT littermates. In disease affected regions (dorsolateral striatum and motor cortex) of BACHD mice but not the less affected dentate gyrus there is a significant increase in the levels of C1q relative to that seen in WT littermates, striatum n = 3 WT and 4 BACHD mice (2 F and 1 M for WT and 2 F and 2 M for BACHD), motor cortex n = 3 WT and 3 BACHD mice (2 F and 1 M for both genotypes) and hippocampus (DG) n = 3 WT and 3 BACHD mice (2 F and 1 M for both genotypes). Unpaired two-tailed t-test for comparisons of WT and BACHD in each brain region, striatum p = 0.017, motor cortex p = 0.008, hippocampus (DG) p = 0.973. **(b)** Bar charts showing quantification of C3 puncta in different brain regions of 13 mo BACHD mice and WT littermates, striatum n = 4 WT and 4 BACHD mice (2 F and 2 M for both genotypes), motor cortex n = 3 WT and 4 BACHD mice (2 F and 1 M for WT and 2 F and 2 M for BACHD) and hippocampus (DG) n = 4 WT and 4 BACHD mice (2 F and 2 M for both genotypes). Unpaired two-tailed t-test for comparisons of WT and BACHD in each brain region, striatum p = 0.011, motor cortex p = 0.001, hippocampus (DG) p = 0.045. **(c)** Representative confocal images of the dorsolateral striatum of 7 mo WT and BACHD mice co-stained with C1q and VGLUT1 and 2 or C3 and VGLUT1 and 2. Note the increased association of both complement proteins with these synaptic markers in the BACHD tissue. Insets show examples of complement proteins co-localized with the presynaptic markers VGLUT1 and 2. Scale bar = 5 μm. Bar charts show quantification of the percentage of VGLUT 1 and 2 puncta colocalizing with C1q or C3 in the disease affected striatum and less disease affected hippocampus (DG), for C1q n = 3 WT and 3 BACHD mice (1 F and 2 M for WT and 2 F and 1 M for BACHD) for C3 n = 3 WT and 4 BACHD mice (2 F and 1 M for WT and 2 F and 2 M for BACHD). Unpaired two-tailed t-test for comparisons of WT and BACHD, Striatum C1q p = 0.004, Hippocampus (DG) C1q p = 0.635, Striatum C3 p = 0.004, Hippocampus (DG) C3 p = 0.364 **(d)** Representative pictographs of Imaris processed super-resolution images from the dorsolateral striatum of 7 mo WT and zQ175 mice co-stained with C3 and VGLUT1 in which the spheres function has been used to reflect immunoreactive puncta. The top two panels show all spheres generated from the super-resolution images and the bottom panels only show C3 and VGLUT1 spheres which are colocalized (defined as a distance of 0.3 μm or less between the center of each sphere). Scale bar = 5 μm. Note that there are more colocalized spheres in the 3 mo zQ175 striatum than in the 3 mo WT striatum. Quantification of these images is shown in the bar charts in Fig. 3g. **(e)** Confocal images of the dorsolateral striatum of 7 mo WT and zQ175 mice co-stained with C1q and VGLUT1 and 2 or C3 and VGLUT1 and 2. Bar charts show quantification of the percentage of VGLUT1 and 2 puncta colocalizing with C1q or C3 in disease affected regions (striatum) or less affected regions (dentate gyrus of the hippocampus), for C1q n = 3 WT and 3 zQ175 mice (3 F and 3 M for both genotypes), for C3 striatum n = 3 WT and 3 zQ175 mice (3 F and 3 M for both genotypes) and for C3 hippocampus (DG) n = 4 WT and 4 zQ175 mice (2 F and 2 M for both genotypes). Unpaired two-tailed t-test for comparisons of WT and zQ175, Striatum C1q p = 0.036, Hippocampus (DG) C1q p = 0.273, Striatum C3 p = 0.010, Hippocampus (DG) C3 p = 0.579. **(f)** Bar chart comparing the fold enrichment of C3 at VGLUT1 puncta in 3 mo zQ175 mice (taken from Fig. 3g) with that same analysis carried out after rotating the C3 channel 90 degrees, n = 4 WT and 4 zQ175 mice (2 F and 2 M for both genotypes). Unpaired two-tailed t-test p = 0.008 **(g)** Representative confocal images of the dorsolateral striatum of 3 mo zQ175 and WT mice co-stained with antibodies to C1q and VGLUT1 or C1q and VGLUT2. Scale bar = 5 μm. Bar charts show quantification of the % of VGLUT1 or VGLUT2 puncta colocalizing with C1q in both genotypes, for VGLUT1 n = 4 WT and 4 zQ175 mice (2 F and 2 M for both genotypes), for VGLUT2 n = 5 WT and 4 zQ175 mice (3 F and 2 M for WT and 2 F and 2 M for zQ175). Unpaired two-tailed t-test for comparisons of WT and zQ175, VGLUT1 p = 0.004, VGLUT2 p = 0.377 **(h)** Representative in situ hybridization (ISH) staining of *C1q* and *NSE* together with IHC for Iba1 in the dorsal striatum of 7 mo zQ175 mice. Inset shows a magnification of a selected area in the field. Scale bar = 20 μm. This experiment was repeated four times. **(i)** Representative ISH staining of *C3* and *Acta2* in the wall of the lateral ventricle. Inset shows a magnification of a selected area in the field Scale bar = 50 μm. This experiment was repeated three times. For bar charts, bars depict the mean. All error bars represent SEM. Stars depict level of significance with *=p < 0.05, **p = <0.01 and ***p < 0.001. Source data

**Extended Data Fig. 4:**
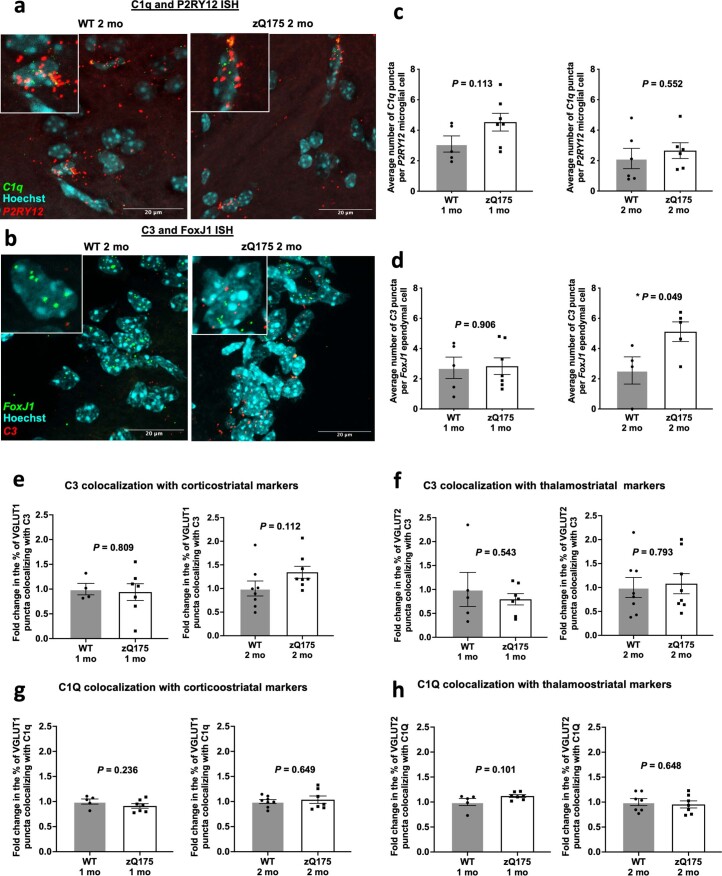
Expression and synaptic localization of complement proteins in HD mouse models. **(a)** Representative in situ hybridization (ISH) staining of *C1q* and *P2RY12* in the dorsal striatum of a 2 mo zQ175 mouse and a WT littermate. **(b)** Representative ISH staining of *C3* and *FoxJ1* in the wall of the lateral ventricle of a 2 mo zQ175 mouse and a WT littermate. For both **a** and **b** scale bar = 20 μm. **(c)** Bar charts showing quantification of RNAScope staining with the average number of IF *C1q* puncta in *P2RY12* expressing microglia (in the dorsal striatum) determined for zQ175 mice and WT littermates at both 1 and 2 mo; 1 mo n = 5 WT and 7 zQ175 (3 F and 2 M for WT and 3 F and 4 M for zQ175) and 2 mo n = 6WT and 6 zQ175 (2 F and 4 M for WT and 3 F and 3 M for zQ175). Unpaired t test, at 1 mo p = 0.113 and at 2 mo p = 0.552. **(d)** Bar charts showing quantification of RNAScope staining with the average number of IF *C3* puncta in FoxJ1 ependymal cells (present in the wall of the lateral ventricle) determined for zQ175 mice and WT littermates at both 1 and 2 mo; 1 mo n = 5 WT and 7 zQ175 (3 F and 2 M for WT and 3 F and 4 M for zQ175) and 2 mo n = 4 WT and 5 zQ175 (2 M and 2 F for WT and 2 F and 3 M for zQ175). Unpaired t test, at 1 mo p = 0.906 and at 2 mo p = 0.049. **(e)** Bar chart showing quantification of the percentage of VGLUT1 puncta colocalizing with C3 in the dorsolateral striatum of 1 and 2 mo zQ175 mice and WT littermates; At 1 mo n = 4 WT and 7 zQ175 mice (2 F and 2 M for WT and 4 M and 3 F for zQ175) and at 2 mo n = 8 WT and 8 zQ175 mice (2 F and 6 M for WT and 3 F and 5 M for zQ175). Unpaired two-tailed t-test, at 1 mo p = 0.809 and at 2 mo p = 0.112. **(f)** Bar chart showing quantification of the percentage of VGLUT2 puncta colocalizing with C3 in the dorsolateral striatum of 1 and 2 mo zQ175 mice and WT littermates; At 1 mo n = 5 WT and 7 zQ175 mice (2 F and 3 M for WT and 4 M and 3 F for zQ175) and at 2 mo n = 8 WT and 8 zQ175 mice (2 F and 6 M for WT and 3 F and 5 M for zQ175). Unpaired two-tailed t-test, at 1 mo p = 0.543 and at 2 mo p = 0.793. **(g)** Bar chart showing quantification of the percentage of VGLUT1 puncta colocalizing with C1Q in the dorsolateral striatum of 1 and 2 mo zQ175 mice and WT littermates; At 1 mo n = 5 WT and 7 zQ175 mice (2 F and 3 M for WT and 4 M and 3 F for zQ175) and at 2 mo n = 8 WT and 7 zQ175 mice (2 F and 6 M for WT and 3 F and 4 M for zQ175). Unpaired two-tailed t-test, at 1 mo p = 0.236 and at 2 mo p = 0.649. **(h)** Bar chart showing quantification of the percentage of VGLUT2 puncta colocalizing with C1Q in the dorsolateral striatum of 1 and 2 mo zQ175 mice and WT littermates; At 1 mo n = 5 WT and 7 zQ175 mice (2 F and 3 M for WT and 4 M and 3 F for zQ175) and at 2 mo n = 7 WT and 7 zQ175 mice (2 F and 5 M for WT and 3 F and 4 M for zQ175). Unpaired two-tailed t-test, at 1 mo p = 0.101 and at 2 mo p = 0.648. All error bars represent SEM. Stars depict level of significance with *=p < 0.05, **p = <0.01 and ***p < 0.001. Source data

Emerging research implicates classical complement cascade activation in synaptic elimination both during normal development and in disease and injury contexts^30–36^. To test whether complement proteins could mediate selective loss of corticostriatal synapses, we investigated whether levels of C1q and C3 were elevated in disease-vulnerable brain regions. IHC analysis revealed that, similar to our findings in the postmortem caudate nucleus of patients with HD, levels of C1q and C3 were elevated in the striatum and motor cortex of 7-month-old zQ175 mice but not in the less disease-affected hippocampus (dentate gyrus)^17,89^ (Fig. 3a,b,d,e). In line with other studies, we found *C1q* to be predominantly expressed by microglia, whereas *C3* was expressed by ependymal cells lining the lateral ventricle wall (Extended Data Fig. 3h,i)^90,91^. Both complement proteins were also significantly elevated at 3 months of age in the dorsolateral striatum of zQ175 mice, correlating with the earliest timepoint that we observed fewer corticostriatal synapses (Fig. 3c,f). Although further investigation showed no changes at 1 month of age, consistent with the absence of synapse loss at this timepoint, expression of *C3* by ependymal cells was already significantly elevated in 2-month-old zQ175 mice, and C3 association with corticostriatal synapses was increased, demonstrating that changes in complement biology occur alongside some of the earliest reported pathologies in this mouse line^71,72^ (Extended Data Fig. 4a–h). These findings were replicated in the BACHD model (Extended Data Fig. 3a,b).

To assess whether C1q and C3 preferentially localize to specific subsets of synapses, we co-stained sections with antibodies to both complement proteins together with markers of corticostriatal and thalamostriatal synapses. We found a significantly higher percentage of glutamatergic synapses co-localized with C1q and C3 in the dorsolateral striatum but not in the hippocampus of 7-month-old zQ175 and BACHD mice relative to wild-type (WT) littermate controls (Extended Data Fig. 3c,e). This is consistent with unbiased proteomic assessments that showed enrichment of C1q in isolated synaptic fractions from the striatum of 6-month-old zQ175 mice^92^. Strikingly, at 3 months of age, when there is a selective loss of corticostriatal synapses in zQ175 mice, we observed an increased percentage of this synaptic population co-localizing with both C3 and C1q but no increased association of complement proteins with neighboring thalamostriatal synapses (Fig. 3g and Extended Data Figs. 3g and 4e–h). To demonstrate that this increase was not a result of a random association, we repeated the analysis with the C3 images rotated 90°, to simulate a chance level of co-localization, and saw that, for zQ175 mice, the association of complement proteins with corticostriatal markers (relative to that seen in WT littermates) was significantly reduced (Extended Data Fig. 3f).

CR3 is expressed exclusively by myeloid cells in the brain and binds to cleaved forms of C3 that opsonize cell membranes, prompting engulfment and elimination of these structures. In the dorsal striatum of 3-month-old zQ175 mice, CR3 levels were significantly increased and localized to microglia processes (Fig. 3h). Collectively, these results show that complement proteins localize specifically to vulnerable corticostriatal synaptic connections before onset of motor and cognitive deficits and that levels of their receptors are elevated on microglial cells, thereby demonstrating that they are present at the right time and place to mediate selective elimination of corticostriatal synapses.

### Microglia engulf corticostriatal projections and synaptic elements in HD mouse models

**Extended Data Fig. 5:**
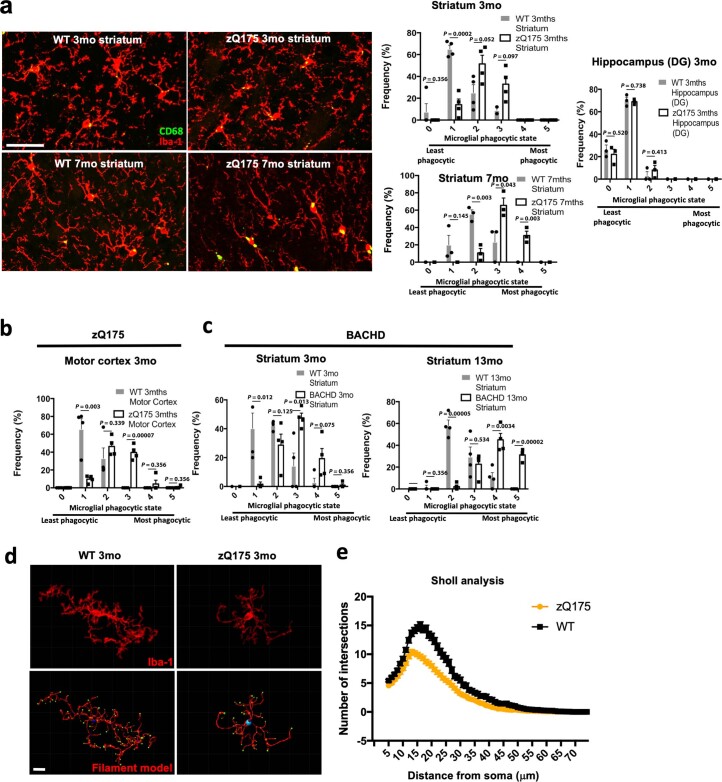
Microglia in the striatum and motor cortex of HD mice adopt a more phagocytic profile. **(a)** Representative maximum intensity projections generated from confocal images of Iba1 and CD68 staining in the dorsal striatum of 3 and 7 mo zQ175 mice and their WT littermates. Scale bar = 20 μm. Note the increased level of the lysosomal marker CD68 and the reduced branching and thicker process of the microglia in the zQ175 mice at both ages. Bar charts show quantification of the phagocytic state of microglia in these images with 5 being the most phagocytic and 0 the least. At both 3 and 7 mo microglia in the dorsal striatum of zQ175 mice show a shift towards a more phagocytic state, for 3 mo n = 4 WT and 4 zQ175 mice (2 F and 2 M for both genotypes); for 7 mo n = 3 WT and 3 zQ175mice (3 F and 3 M for both genotypes). Multiple unpaired two-tailed t-tests, at 3 mo for score 0 p = 0.356, for score 1 p = 0.0002, for score 2 p = 0.052, for score 3 p = 0.097; at 7 mo for score 1 p = 0.145, for score 2 p = 0.003, for score 3 p = 0.043, for score 4 p = 0.003. This is not the case in less disease affected regions such as the dentate gyrus of the hippocampus, n = 3 WT and 3 zQ175 mice. Multiple unpaired two-tailed t-tests for score 0 p = 0.520, for score 1 p = 0.738, for score 2 p = 0.413. **(b)** Bar chart showing quantification of microglial phagocytic state in the motor cortex of 3 mo zQ175 mice and WT littermates. There is a shift towards a more phagocytic state in the zQ175 mice, n = 4 WT and 4 zQ175 mice (2 F and 2 M for both genotypes). Multiple unpaired two-tailed t-tests for score 1 p = 0.003, for score 2 p = 0.339, for score 3 p = 0.00007, for score 4 p = 0.356, for score 5 p = 0.356. **(c)** Bar charts, showing that there is also a shift towards a more phagocytic microglial state in the striatum of 3 mo and 13 mo BACHD mice, for both 3 mo and 13 mo n = 4 WT and 4 BACHD mice (2 F and 2 M for both genotypes). Multiple unpaired two-tailed t-tests for 3 mo score 1 p = 0.012, for score 2 p = 0.125, for score 3 p = 0.013, for score 4 p = 0.075 and for score 5 p = 0.356; for 13 mo score 1 p = 0.356, for score 2 p = 0.00005, for score 3 p = 0.534, for score 4 p = 0.0034, score 5 p = 0.00002. **(d)** Confocal images and filament renderings of individual microglia stained with Iba1 in the dorsal striatum of 3 mo zQ175 mice and WT littermates. Scale bar = 10 μm. In the filament renderings the blue sphere indicates the soma, orange spheres denote branch points and green spheres indicate terminal points of microglial processes. **(e)** Sholl analysis of confocal images of microglia from the dorsal striatum of 3 mo zQ175 mice and WT littermates. Analysis was performed on filament rendered images using Imaris software, n = 3 WT and 4 zQ175 mice (2 F and 1 M for WT and 2 F and 2 M for zQ175) with over 100 cells analyzed per genotype. Two way anova, p = <0.0001 with Sidak’s multiple comparisons test shows a significant difference between WT and zQ175 at distances from the soma ranging from 11 to 30 μm p < 0.0001. For bar charts, bars depict the mean. All error bars represent SEM. Stars depict level of significance with *=p < 0.05, **p = <0.01 and ***p < 0.001. Source data

**Extended Data Fig. 6:**
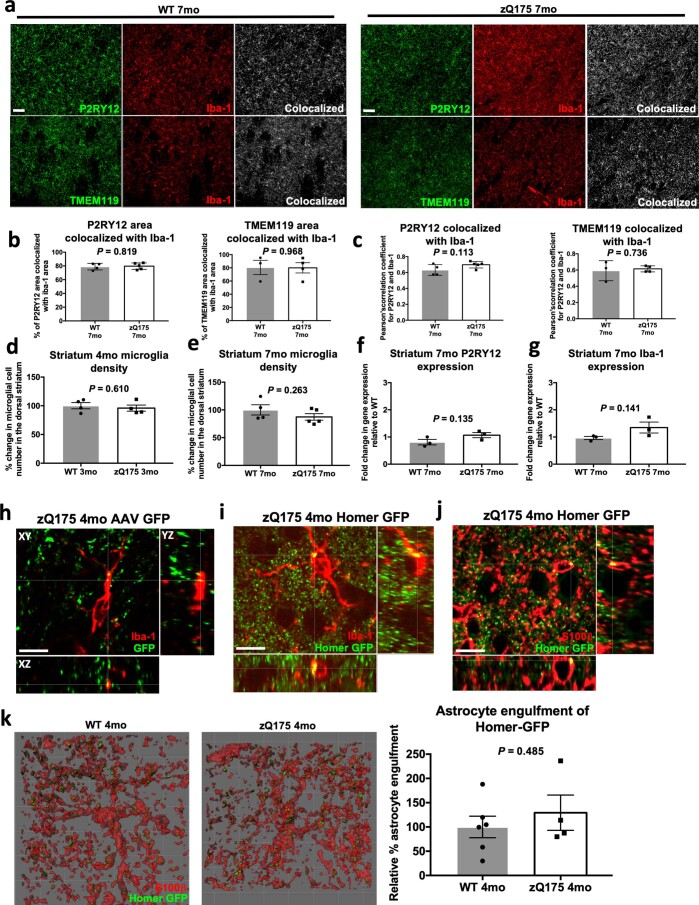
Microglia density and their levels of identity markers do not change in HD mice and striatal astrocytes do not engulf a greater amount of synaptic material. **(a)** Confocal images of microglia in the dorsal striatum of 7 mo zQ175 mice and WT littermates co-stained with antibodies to Iba1 and putative microglia identity markers P2RY12 and TMEM119. Scale bar = 50 μm **(b)** Bar charts show the % of P2RY12 and TMEM119 immunoreactive area above a set threshold (determined using an algorithm developed by Costes and Lockett^203^) that colocalizes with the area of Iba1 staining for the images in **(a)**, for P2RY12 and Iba1 n = 4 WT and 5 zQ175 mice (2 F and 2 M for WT and 3 F and 2 M for zQ175); for TMEM119 and Iba1 n = 3 WT and 4 zQ175 mice (2 F and 1 M for WT and 2 M and 2 F for zQ175). Unpaired two-tailed t-test for comparison of WT and zQ175 mice, for P2RY12 and Iba1 p = 0.819 and for TMEM119 and Iba1 p = 0.968. **(c)** Bar charts show the Pearson’s correlation coefficient for Iba1 and P2RY12 and Iba1 and TMEM119 for the images shown in **(a)**, for P2RY12 and Iba1 n = 4 WT and 5 zQ175 mice (2 F and 2 M for WT and 3 F and 2 M for zQ175); for TMEM119 and Iba1 n = 3 WT and 4 zQ175 mice (2 F and 1 M for WT and 2 M and 2 F for zQ175). Unpaired t test for comparison of WT and zQ175 mice P2RY12 and Iba1 p = 0.113 and for TMEM119 and Iba1 p = 0.736. **(d)** Bar chart shows quantification of microglial cell density in the dorsal striatum of 4 mo zQ175 mice and WT littermates, n = 4 WT and 4 zQ175 mice (2 F and 2 M for both genotypes). Unpaired two-tailed t-test p = 0.610. **(e)** Bar chart shows quantification of microglial cell density in the striatum of 7 mo Q175mice and WT littermates, n = 4 WT and 5 zQ175 mice (2 F and 2 M for WT and 3 F and 2 M for zQ175). Unpaired two-tailed t-test p = 0.263 **(f)** Bar chart shows the level of P2RY12 transcripts in striatal extracts from 7 mo zQ175 mice and WT littermates, n = 3 WT and 3 zQ175 mice (3 F and 3 M for both genotypes). Unpaired two-tailed t-test p = 0.135. **(g)** Bar chart shows the level of Iba1 transcripts in striatal extracts from 7 mo zQ175 and mice and WT littermates, n = 3 WT and 3 zQ175 mice (3 F and 3 M for both genotypes). Unpaired two-tailed t-test p = 0.141. **(h)** Representative orthogonal view of an Iba1 stained microglia in the dorsal striatum of a 4 mo zQ175 mice that had previously received a motor cortex injection of pAAV2-hsyn-EGFP at P1/2. Scale bar = 10 μm This experiment was repeated 6 times. **(i)** Representative orthogonal view of an Iba1 stained microglia in the dorsal striatum of a 4 mo zQ175 Homer-GFP mouse. Scale bar = 10 μm. This experiment was repeated 5 times. **(j)** Representative orthogonal view of an S100β stained astrocyte in the dorsal striatum of a 4 mo zQ175 Homer-GFP mouse. Scale bar = 10 μm. This experiment was repeated four times. **(k)** Representative surface rendered images of S100β stained astrocytes (red) and engulfed Homer-GFP inputs (green) in the dorsal striatum of 4 mo zQ175 Homer-GFP mice and WT Homer-GFP littermates. Scale bar = 10 μm. Bar chart shows quantification of the relative % astrocyte engulfment of Homer-GFP (the volume of engulfed Homer-GFP expressed as a percentage of the total volume of the astrocyte) in 4 mo zQ175 Homer-GFP mice relative to that seen in WT Homer-GFP littermate controls, n = 6 WT Homer-GFP and 4 zQ175 Homer-GFP mice (3 F and 3 M for WT Homer-GFP and 2 F and 2 M for zQ175 Homer-GFP mice). Unpaired two-tailed t-test, p = 0.485. For bar charts, bars depict the mean. All error bars represent SEM. Stars depict level of significance with *=p < 0.05, **p = <0.01 and ***p < 0.001. Source data

To test whether microglia engulf synaptic elements in HD models, we first investigated region-specific changes in microglia phenotypes consistent with adoption of a more phagocytic state^93,94^. Using an established combination of markers that incorporates both changes in cell morphology (Iba1) and CD68 lysosomal protein levels^30,95^, we found a significant shift toward a more phagocytic microglial phenotype in the striatum and motor cortex (another disease-affected brain region^96,97^) of 3-month-old and 7-month-old zQ175 mice. However, this was not seen in the less disease-affected hippocampus (Extended Data Fig. 5a,b). Similar changes in morphology were observed using Sholl analysis, and a shift in phagocytic state was also seen in the striatum of BACHD mice (Extended Data Fig. 5c–e). To establish that the observed cells were not invading monocytes, we co-stained with Iba1 and microglia identity markers Tmem119 and P2ry12 (Extended Data Fig. 6a–c)^98,99^. Like complement proteins, phagocytic microglia are, thus, present at the right time and place to play a role in synaptic elimination.

**Fig. 4:**
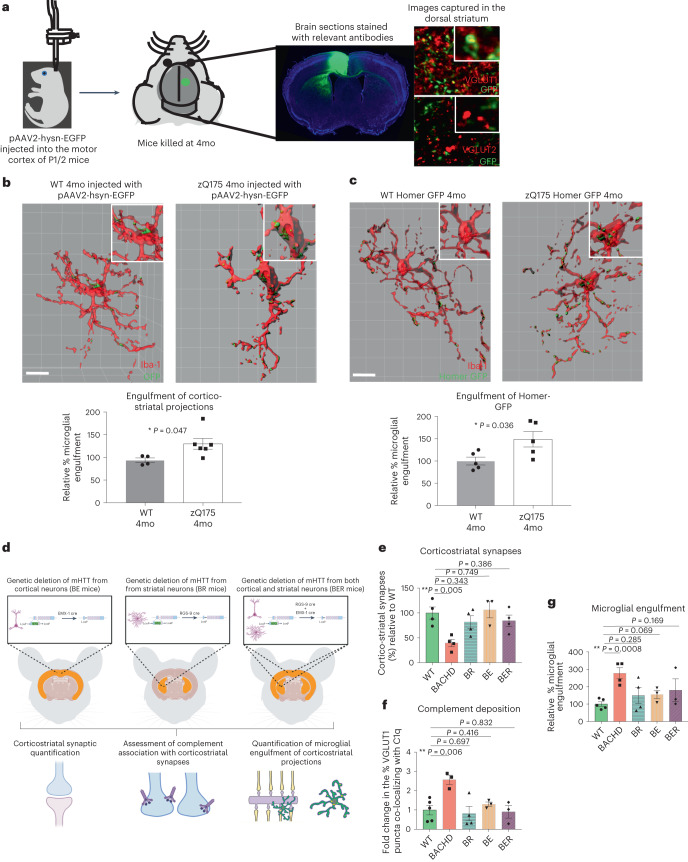
Microglia in the striatum of HD mice engulf more corticostriatal projections and synaptic material in a manner that is dependent on expression of neuronal mHTT. **a**, Schematic showing labeling of corticostriatal projections with pAAV2-hsyn-EGFP injection into the motor cortex of neonatal mice. Panels to the right show co-localization of GFP signal in the dorsal striatum with VGLUT1, a marker of the corticostriatal synapse, but not VGLUT2, a marker of the thalamostriatal synapse. **b**, Representative surface-rendered images of Iba1^+^ microglia and engulfed GFP-labeled corticostriatal projections in the dorsal striatum of 4-month-old zQ175 and WT mice. Scale bar, 10 μm. Bar chart shows quantification of the relative % microglia engulfment (the volume of engulfed inputs expressed as a percentage of the total volume of the microglia) in 4-month-old zQ175 mice relative to that seen in WT littermate controls. All engulfment values were normalized to the total number of inputs in the field, *n* = 4 WT and 6 zQ175 mice. Unpaired two-tailed *t*-test, *P* = 0.047. **c**, Representative surface-rendered images of Iba1^+^ microglia and engulfed Homer-GFP in the dorsal striatum of 4-month-old zQ175 Homer-GFP mice and WT Homer-GFP littermates. Scale bar, 10 μm. Bar charts show quantification of the relative % microglia engulfment of Homer-GFP in 4-month-old zQ175 Homer-GFP mice relative to that seen in WT Homer-GFP littermate controls, *n* = 5 WT Homer-GFP and 5 zQ175 Homer-GFP mice. Unpaired two-tailed *t*-test, *P* = 0.036. **d**, Schematic showing the strategy that we adopted to genetically ablate mHTT from striatal neurons or cortical neurons or both populations before interrogating corticostriatal synaptic density, complement association with corticostriatal markers and microglial-mediated engulfment of these synapses in these mice. **e**, Quantification of the % of corticostriatal synapses in the dorsal striatum of BACHD, BR (deletion of mHTT in striatal neurons), BE (deletion of mHTT in cortical neurons) and BER (deletion of mHTT in striatal and cortical neurons) mice normalized to those seen in WT littermates, *n* = 4 WT mice, 4 BACHD mice, 4 BR mice, 3 BE mice and 4 BER mice. Unpaired two-tailed *t*-test with comparison to WT, BACHD *P* = 0.005, BR *P* = 0.343, BE *P* = 0.749 and BER *P* = 0.386. **f**, % of VGLUT1 puncta co-localized with C1q in the dorsal striatum of BACHD, BR, BE and BER mice and WT littermate controls, *n* = 5 WT mice, 3 BACHD mice, 4 BR mice, 3 BE mice and 3 BER mice. Unpaired two-tailed *t*-test with comparison to WT, BACHD *P* = 0.006, BR *P* = 0.697, BE *P* = 0.416 and BER *P* = 0.832. **g**, Quantification of the relative % microglial engulfment of corticostriatal projections in the dorsal striatum of BACHD, BR, BE, BER and WT mice, *n* = 5 WT mice, 4 BACHD mice, 4 BR mice, 3 BE mice and 3 BER mice. Unpaired two-tailed *t*-test with comparison to WT, BACHD *P* = 0.0008, BR *P* = 0.285, BE *P* = 0.069 and BER *P* = 0.169. For bar charts, bars depict the mean. All error bars represent s.e.m. *P < 0.05, **P < 0.01 and ***P < 0.001. mo, months old. Source data

To directly test whether microglia engulf more corticostriatal inputs in HD mice, we labeled cortical neurons with a stereotactic injection of pAAV2-hsyn-EGFP into the motor cortex of PD1 zQ175 and WT littermate mice (schematized in Fig. 4a). Microglial engulfment in the ipsilateral dorsal striatum was then assessed at 4 months of age (as described in refs. ^95,100^). We found that microglia in the striatum of 4-month-old zQ175 mice contained a greater volume of labeled projections compared to WT littermate controls (Fig. 4b and Extended Data Fig. 6h). Notably, no differences in the number of Iba1-stained cells or transcripts for microglia-specific markers, which could have confounded interpretation of these data, were detected in the zQ175 striatum (Extended Data Fig. 6d–g).

As this viral labeling method does not distinguish between engulfment of presynaptic bouton structures and axonal material, which might come from exosomes released from neurons or axosome shedding, we used a transgenic method to specifically assay engulfment of synaptic elements. zQ175 mice were crossed with mice in which postsynaptic marker homer1c is fused to a GFP tag (hereafter referred to as homer-GFP)^101^. Microglia in the dorsal striatum of zQ175 mice engulfed significantly more homer-GFP than those of their WT littermates (Fig. 4c and Extended Data Fig. 6i). Interestingly, in line with this result, a recent ultrastructural study found that microglia process interaction with synaptic clefts was also increased before onset of motor deficits in a different HD mouse model^51^. To ensure that expression of the transgene had not affected pathological synapse loss, we quantified homer-GFP puncta and Homer1 immunoreactive puncta at 7 months of age and saw the expected reduction of both in the zQ175 homer-GFP mice relative to homer-GFP littermates (Supplementary Fig. 1a,b). Collectively, these results show that microglia adopt a more phagocytic phenotype and engulf more synaptic material in the dorsal striatum of zQ175 mice.

Astrocyte dysfunction is linked to corticostriatal circuit abnormalities and motor and cognitive dysfunctions in HD mouse models, and astrocytes can also phagocytose synaptic material during developmental refinement^102–105^. To test whether astrocytes engulf corticostriatal synapses in HD models, we stained sections from Homer-GFP and zQ175 Homer-GFP mice with astrocyte marker S100β and performed engulfment analysis. Although astrocytes engulf synaptic elements in the striatum, the average volume of engulfed material was not altered in zQ175 mice relative to that seen in WT littermates (Extended Data Fig. 6j,k), demonstrating that engulfment of synaptic material by astrocytes is unlikely to play a significant role in the early synaptic loss that we observed.

### Microglial engulfment and synapse loss are driven by mutant huntingtin expression in both cortical neurons and MSNs in the striatum

Mutant huntingtin (mHTT) is expressed ubiquitously; however, previous work demonstrated that specific HD pathologies can be driven by mHTT expression in different cell types^10,19,49,106–111^. To determine if elimination of corticostriatal synapses requires mHTT in both cortical and striatal neurons, we quantified synapse density, complement deposition and microglial engulfment in BACHD mice in which mHTT had been selectively ablated from the cortex (using Emx1-cre, ‘BE’ mice), striatum (using RGS9-cre ‘BR’ mice) or both (using both Emx-1 and RGS9-cre, ‘BER’ mice)^19^ (schematized in Fig. 4d and Supplementary Fig. 1g). We found that genetic deletion of mHTT from the cortex, striatum or both completely rescued the loss of synapses normally seen in the BACHD model (Fig. 4e), consistent with a previous study showing that synaptic marker loss and corticostriatal synaptic transmission deficits are ameliorated by genetically deleting mHTT in either striatal or cortical neurons^19^. In addition, deposition of complement component C1q, as well as microglial engulfment, was reduced to WT levels (Fig. 4f,g). Thus, mHTT expression in both MSNs and cortical neurons is specifically required to initiate the complement deposition, microglial engulfment of synaptic elements and synapse loss that we observed in the BACHD model.

### Early loss of corticostriatal synapses is rescued by inhibiting activation of the classical complement pathway or genetically ablating microglial CR3

**Fig. 5:**
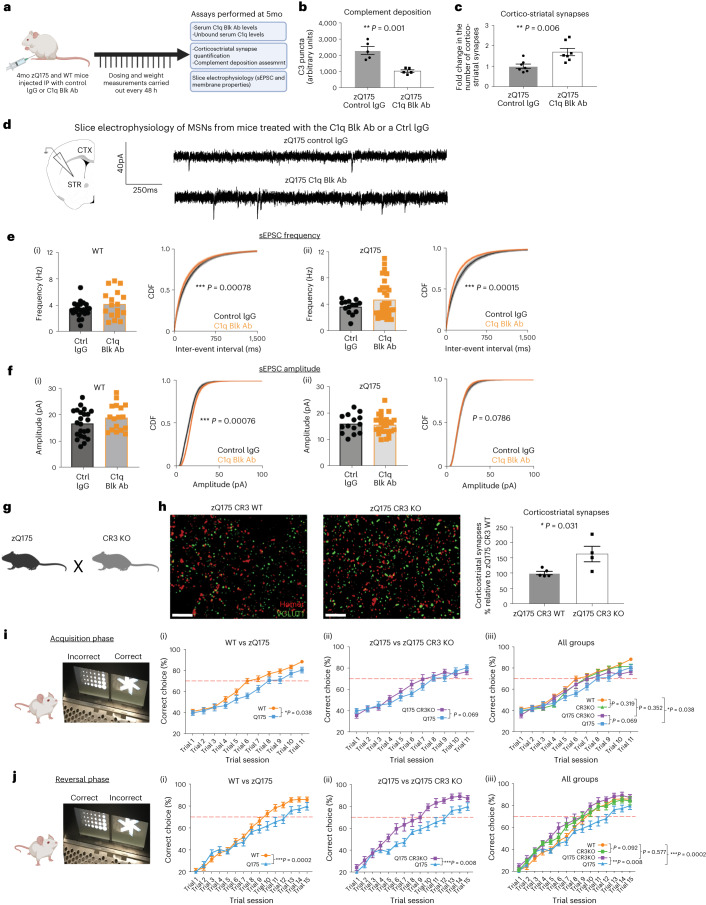
Blocking complement deposition or microglial recognition of complement opsonized structures can reduce synaptic loss and prevent the development of cognitive deficits in HD mice. **a**, Schematic showing the experimental paradigm in which 4-month-old zQ175 mice and WT littermates were treated with the C1q Blk Ab or a control IgG for 1 month before being euthanized. **b**, Quantification of C3 puncta in the dorsolateral striatum of zQ175 mice treated with the C1q function-blocking antibody or a control IgG, *n* = 5 zQ175 mice treated with control IgG and 5 zQ175 mice treated with C1q function-blocking antibody. Unpaired two-tailed *t*-test, *P* = 0.001. **c**, Quantification of corticostriatal synapses in the dorsolateral striatum of zQ175 mice treated with the C1q function-blocking antibody or a control IgG, *n* = 7 zQ175 mice treated with control IgG and 6 zQ175 mice with the C1q function-blocking antibody. Unpaired two-tailed *t*-test, *P* = 0.006. **d**, Diagram depicting the strategy for carrying out electrophysiology recordings from coronal sections of treated mice and representative traces of sEPSCs recorded from MSNs in striatal slices from 5-month-old zQ175 mice that had been treated for 1 month with control IgG or the C1q function-blocking antibody. **e**, Cumulative distribution plots of inter-spike intervals (ISIs) obtained from whole-cell voltage-clamp recordings of MSN sEPSCs. Recordings were carried out in slices from WT and zQ175 mice after a 1-month treatment with control IgG or the C1q function-blocking antibody (Black = Control IgG, Orange = C1q function-blocking antibody). Bar charts show average frequency (Hz) per cell recorded across conditions. *n* = 23 cells from 7 mice for WT Ctrl IgG; *n* = 17 cells from 7 mice for WT C1q Blk Ab; *n* = 14 cells from 4 mice for zQ175 Ctrl IgG; and *n* = 23 cells from 7 mice for zQ175 C1q Blk Ab. For the cumulative distribution plots for WT, *P* = 0.00078, and for zQ175, *P* = 0.00015. **f**, Cumulative distribution plots of amplitude obtained from the same whole-cell voltage-clamp recordings of MSN sEPSCs as in **e**. Black = Control IgG, Orange = C1q function-blocking antibody. Bar charts show average amplitude (pA) per cell recorded across conditions for the same cells/mice represented in **e**. For the cumulative distribution plots, for WT *P* = 0.00076 and for zQ175 *P* = 0.0786. **g**, Diagram showing the two transgenic mice lines crossed together to generate the genotypes employed in assessments of visual discrimination learning and reversal performance. **h**, Representative SIM images of Homer1 and VGLUT1 staining in the dorsolateral striatum of 4-month-old zQ175 CR3 WT and zQ175 CR3 KO mice. Scale bar, 5 μm. Bar chart shows quantification of corticostriatal synapses, *n* = 5 zQ175 CR3 WT mice and 4 zQ175 CR3 KO mice. Unpaired two-tailed *t*-test, *P* = 0.031. **i**, After completion of shaping tasks (see Methods for details), the visual discrimination performance of 4-month-old WT, zQ175, CR3 KO and zQ175 CR3 KO mice was assessed using the Bussey–Sakisda operant touchscreen platform. Line charts show performance over 11 trial sessions (60 trials per session) of (i) WT and zQ175 mice, (ii) zQ175 and zQ175 CR3 KO mice and (iii) all groups, *n* = 29 WT mice, 18 zQ175 mice, 24 CR3KO mice and 13 zQ175 CR3KO mice. Two-way ANOVA: for WT versus zQ175 *P* = 0.038 for the combination of genotype × trial session as a significant source of variation and *P* = 0.01 for genotype as a significant source of variation; for zQ175 versus zQ175 CR3KO *P* = 0.069 for the combination of genotype × trial session as a significant source of variation and *P* = 0.568 for genotype as a significant source of variation; for WT versus zQ175 CR3KO *P* = 0.352 for the combination of genotype and trial as a significant source of variation and *P* = 0.068 for genotype as a significant source of variation; for WT versus CR3 KO *P* = 0.319 for the combination of genotype × trial session as a significant source of variation and *P* = 0.593 for genotype as a significant source of variation. **j**, After completion of the acquisition phase, visual presentations were switched so that the visual stimuli previously associated with reward provision were now the incorrect choice. Performance of WT, zQ175, CR3 KO and zQ175 CR3 KO mice in this reversal phase of the task was then assessed using the Bussey–Sakisda operant touchscreen platform. Line charts show performance over 15 trial sessions (60 trials per session) of (i) WT and zQ175 mice, (ii) zQ175 and zQ175 CR3 KO mice and (iii) all groups, for the same mice tested in **i**. Two-way ANOVA: for WT versus zQ175 *P* = 0.0002 for the combination of genotype × trial session as a significant source of variation and *P* = 0.080 for genotype as a significant source of variation; for zQ175 versus zQ175 CR3KO *P* = 0.008 for the combination of genotype × trial session as a significant source of variation and *P* = 0.003 for genotype as a significant source of variation; for WT versus zQ175 CR3KO *P* = 0.577 for the combination of genotype and trial as a significant source of variation and *P* = 0.072 for genotype as a significant source of variation; for WT versus CR3 KO *P* = 0.092 for the combination of genotype × trial session as a significant source of variation and *P* = 0.304 for genotype as a significant source of variation. For bar charts, bars depict the mean, and all error bars represent s.e.m. *P < 0.05, **P < 0.01 and ***P < 0.001. CDF, cumulative distribution function. Source data

**Extended Data Fig. 7:**
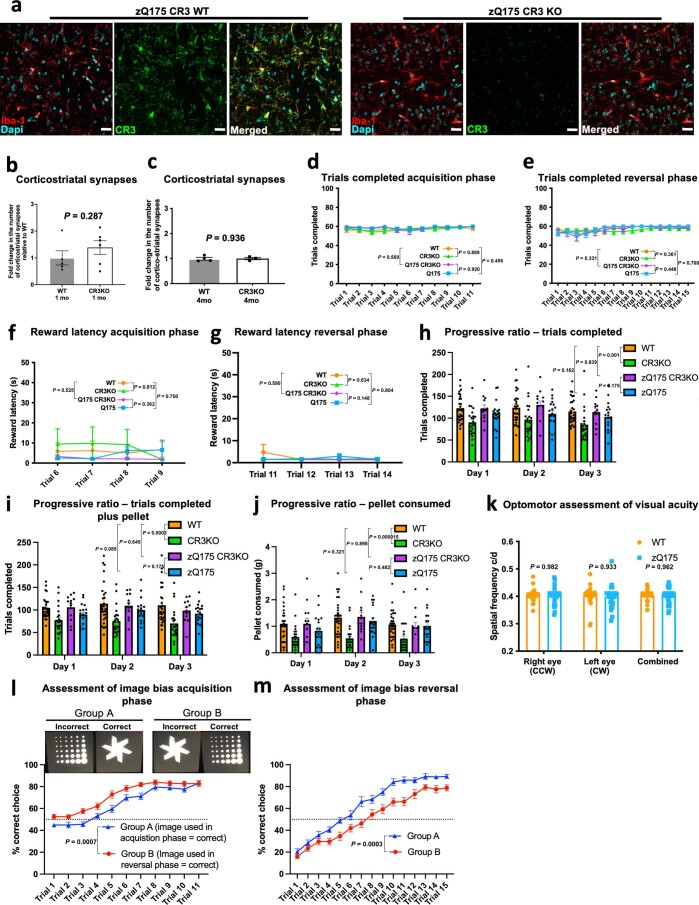
Blocking complement deposition can reduce synaptic loss in HD mice. **(a)** Representative confocal images of the dorsal striatum of 4 mo zQ175 CR3 WT and 4 mo zQ175 CR3 KO mice stained with antibodies to Iba-1 and CR3. Scale bar = 20 µm. For bar charts, bars depict the mean and all error bars represent SEM. Stars depict level of significance with *=p < 0.05, **p = <0.01 and ***p < 0.0001. **(b)** Bar chart showing quantification of corticostriatal synapses in the dorsolateral striatum of 1 mo CR3KO mice and WT littermates, n = 5 WT mice and 6 CR3KO mice. Unpaired two-tailed t-test p = 0.287. **(c)** Bar chart showing quantification of corticostriatal synapses in the dorsolateral striatum of 4 mo CR3KO mice and WT littermates, n = 4 WT mice and 3 CR3KO mice (2 F and 2 M for WT and 2 F and 1 M for CR3KO). Unpaired two-tailed t-test p = 0.9356. **(d)** Line graph showing the mean number of trials completed on each trial day for each genotype during the acquisition phase (maximum = 60), n = 29 WT mice (16 M, 13 F), 18 zQ175 mice (8 M, 10 F), 24 CR3KO mice (13 M, 11 F) and 13 zQ175 CR3KO mice (9 M, 4 F). Two way anova: for WT vs zQ175 p = 0.535 for the combination of genotype x trial session as a significant source of variation and p = 0.456 for genotype as a significant source of variation (shown on graph); for zQ175 vs zQ175 CR3KO p = 0.501 for the combination of genotype x trial session as a significant source of variation and p = 0.920 for genotype as a significant source of variation (shown on graph); for WT vs zQ175 CR3KO p = 0.689 for the combination of genotype x trial session as a significant source of variation and p = 0.569 for genotype as a significant source of variation (shown on graph); for WT vs CR3 KO p = 0.908 for the combination of genotype x trial session as a significant source of variation and p = 0.889 for genotype as a significant source of variation (shown on graph). **(e)** Line graph showing the mean number of trials completed on each trial day for each genotype during the reversal phase (maximum = 60), n = 29 WT mice, 18 zQ175 mice, 24 CR3KO mice and 13 zQ175 CR3KO mice. Two way anova: for WT vs zQ175 p = 0.787 for the combination of genotype x trial session as a significant source of variation and p = 0.708 for genotype as a significant source of variation; for zQ175 vs zQ175 CR3KO p = 0.899 for the combination of genotype x trial session as a significant source of variation and p = 0.448 for genotype as a significant source of variation; for WT vs zQ175 CR3KO p = 0.960 for the combination of genotype x trial as a significant source of variation and p = 0.331 for genotype as a significant source of variation; for WT vs CR3 KO p = 0.132 for the combination of genotype x trial session as a significant source of variation and p = 0.361 for genotype as a significant source of variation. **(f)** Line graph showing reward latency for each genotype for trial days 6,7,8 and 9 in the acquisition phase, n = 29 WT mice, 18 zQ175 mice, 24 CR3KO mice and 13 zQ175 CR3KO mice. Two way anova: for WT vs zQ175 p = 0.499 for the combination of genotype x trial session as a significant source of variation and p = 0.750 for genotype as a significant source of variation; for zQ175 vs zQ175 CR3KO p = 0.526 for the combination of genotype x trial session as a significant source of variation and p = 0.362 for genotype as a significant source of variation; for WT vs zQ175 CR3KO p = 0.868 for the combination of genotype x trial as a significant source of variation and p = 0.525 for genotype as a significant source of variation; for WT vs CR3 KO p = 0.240 for the combination of genotype x trial session as a significant source of variation and p = 0.812 for genotype as a significant source of variation. **(g)** Line graph showing reward latency for each genotype for trial days 11,12,13 and 14 in the reversal phase, n = 29 WT mice, 18 zQ175 mice, 24 CR3KO mice and 13 zQ175 CR3KO mice. Two way anova: for WT vs zQ175 p = 0.487 for the combination of genotype x trial session as a significant source of variation and p = 0.804 for genotype as a significant source of variation; for zQ175 vs zQ175 CR3KO p = 0.271 for the combination of genotype x trial session as a significant source of variation and p = 0.148 for genotype as a significant source of variation; for WT vs zQ175 CR3KO p = 0.751 for the combination of genotype x trial as a significant source of variation and p = 0.590 for genotype as a significant source of variation; for WT vs CR3 KO p = 0.431 for the combination of genotype x trial session as a significant source of variation and p = 0.634 for genotype as a significant source of variation. **(h)** Bar chart showing the average total number of trials completed by each genotype in the progressive ratio task, n = 29 WT mice, 18 zQ175 mice, 24 CR3KO mice and 13 zQ175 CR3KO mice. Two way anova: for WT vs zQ175 p = 0.919 for the combination of genotype x testing day as a significant source of variation and p = 0.162 for genotype as a significant source of variation; for zQ175 vs zQ175 CR3KO p = 0.391 for the combination of genotype x testing day as a significant source of variation and p = 0.175 for genotype as a significant source of variation; for WT vs zQ175 CR3KO p = 0.583 for the combination of genotype x testing day as a significant source of variation and p = 0.839 for genotype as a significant source of variation; for WT vs CR3 KO p = 0.754 for the combination of genotype x testing day as a significant source of variation and p = 0.0012 for genotype as a significant source of variation. **(i)** Bar chart showing the average total number of trials completed by each genotype in the progressive ratio task when the assay is conducted in the presence of a food pellet, n = 29 WT mice, 18 zQ175 mice, 24 CR3KO mice and 13 zQ175 CR3KO mice. Two way anova: for WT vs zQ175 p = 0.813 for the combination of genotype x testing day as a significant source of variation and p = 0.085 for genotype as a significant source of variation; for zQ175 vs zQ175 CR3KO p = 0.592 for the combination of genotype x testing day as a significant source of variation and p = 0.175 for genotype as a significant source of variation; for WT vs zQ175 CR3KO p = 0.247 for the combination of genotype x testing day as a significant source of variation and p = 0.645 for genotype as a significant source of variation; for WT vs CR3 KO p = 0.051 for the combination of genotype x testing day as a significant source of variation and p = 0.0003 for genotype as a significant source of variation. **(j)** Bar chart showing the amount in grams of a food pellet consumed by each genotype while carrying out the progressive ratio task, n = 29 WT mice, 18 zQ175 mice, 24 CR3KO mice and 13 zQ175 CR3KO mice. Two way anova: for WT vs zQ175 p = 0.470 for the combination of genotype x testing day as a significant source of variation and p = 0.321 for genotype as a significant source of variation; for zQ175 vs zQ175 CR3KO p = 0.399 for the combination of genotype x testing day as a significant source of variation and p = 0.482 for genotype as a significant source of variation; for WT vs zQ175 CR3KO p = 0.826 for the combination of genotype x testing day as a significant source of variation and p = 0.898 for genotype as a significant source of variation; for WT vs CR3 KO p = 0.153 for the combination of genotype x testing day as a significant source of variation and p = 0.000015 for genotype as a significant source of variation. **(k)** Bar chart showing the average performance of WT and zQ175 mice in the optomotor assay of visual acuity, n = 33 WT mice (18 M, 15 F) and 28 zQ175 mice (15 M, 13 F). Two way anova for WT vs zQ175 p = 0.598 for genotype as a significant source of variation with p = 0.982, 0.933 and 0.962 for the right eye, left eye or the combined performance of both respectively via Sidak’s multiple comparisons test. **(l)** Line graph showing image bias during the acquisition phase of the visual discrimination task, n = 15 WT mice in group A (where the image presentation is the same as that used for the testing in Fig. 5i) and 15 WT mice in group B (where the image presentation is the reverse of the used for the testing in Fig. 5i). Two way anova for Group A vs Group B p = 0.088 for group x trial as a significant source of variation and p = 0.0007 for group as a significant source of variation. **(m)** Line graph showing image bias during the reversal phase of the task, n = 15 WT mice in group A (9 M, 6 F) and 15 WT mice in group B (10 M, 5 F). Two way anova for Group A vs Group B p = 0.014 for genotype x group as a significant source of variation and p = 0.0003 for genotype as a significant source of variation. For bar charts, bars depict the mean and all error bars represent SEM. Stars depict level of significance with *=p < 0.05, **p = <0.01 and ***p < 0.001. Source data

To test whether increased synaptic complement deposition and engulfment of corticostriatal inputs by microglia mediates selective elimination of these synapses in zQ175 mice, we blocked these processes using several approaches. To prevent microglial interaction with bound complement proteins, we genetically ablated CR3 in zQ175 mice (Extended Data Fig. 7a). This increased the number of corticostriatal synapses present in the dorsolateral striatum by 60% relative to zQ175 littermates expressing CR3 (Fig. 5g,h). This increase was not due to persistent effects related to differences in developmental pruning, as has been observed at the retinogeniculate synapse in CR3KO mice, or other aspects of altered biology, as CR3 ablation on a WT background did not alter corticostriatal synapse number at either 1 month or 4 months of age (Extended Data Fig. 7b,c). CR3 ablation also did not change the percentage of corticostriatal synapses found to be associated with C1q or C3, confirming that deposition of these proteins is an upstream event in the synaptic elimination mechanism and also that these complement proteins are not immediately cleared from synaptic terminals through other mechanisms (Extended Data Fig. 9d,e).

**Extended Data Fig. 8:**
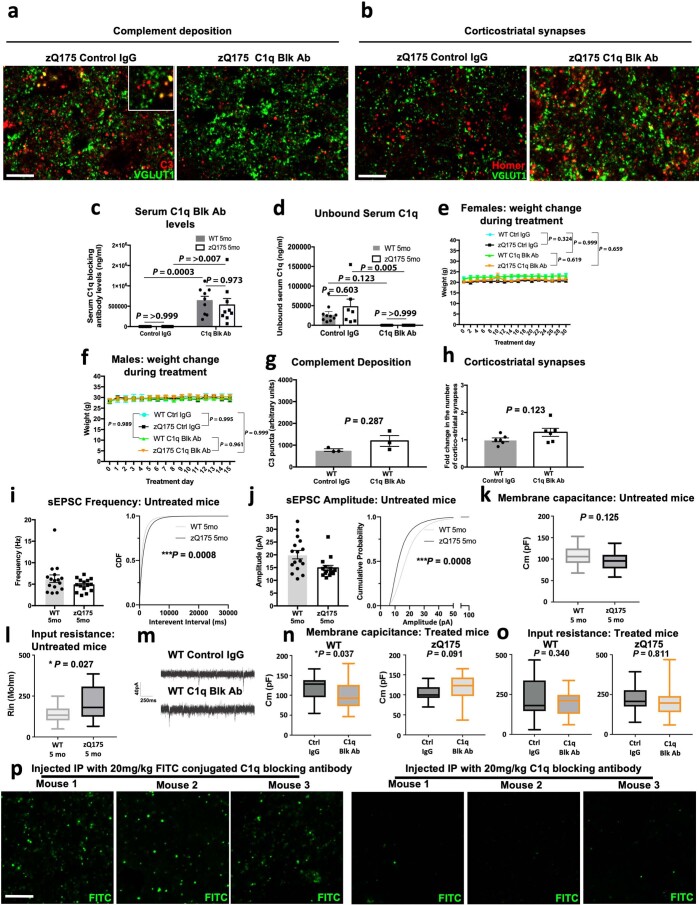
Preventing microglial recognition of complement opsonized structures can reduce synaptic loss and prevent the development of cognitive deficits in HD mice. **(a)** Representative confocal images of VGLUT1 and C3 staining in the dorsolateral striatum of 4 mo zQ175 mice that received intraperitoneal injections of a C1q function blocking antibody (M1) or a control IgG. Scale bar = 5 μm. **(b)** Representative confocal images of Homer1 and VGLUT1 staining in the dorsolateral striatum of zQ175 mice treated with the C1q function blocking antibody or a control IgG. Scale bar = 5 μm. **(c)** Bar chart showing quantification of the serum levels of the C1q function blocking antibody in 5 mo zQ175 mice and WT littermates following 1 mo treatment with either the blocking antibody or a control IgG, n = 10 WT mice with control IgG (5 F and 5 M); 9 WT mice with C1q Blk Ab (4 F and 5 M); 7 zQ175 mice with control IgG (3 F and 4 M); 9 zQ175 mice with C1q Blk Ab (5 F and 4 M). Two way anova p = <0.0001 for treatment type and p = 0.6 for genotype with WT control IgG vs zQ175 control IgG p = >0.999; WT control IgG vs WT C1q Blk Ab p = 0.0003; WT control IgG vs zQ175 C1q Blk Ab p = 0.003; zQ175 control IgG vs WT C1q Blk Ab p = 0.001; zQ175 control IgG vs zQ175 C1q Blk Ab p = 0.007; and WT C1q Blk Ab vs zQ175 C1q Blk Ab p = 0.973 via Sidak’s multiple comparisons test. **(d)** Bar chart showing levels of unbound C1q in the same serum tested in **(c)**, n = 10 WT mice with control IgG; 9 WT mice with C1q Blk Ab; 7 zQ175 mice with control IgG; 9 zQ175 mice with C1qBlkAb. Two way anova p = 0.0001 for treatment type and p = 0.286 for genotype with WT control IgG vs zQ175 control IgG p = 0.603; WT control IgG vs WT C1q Blk Ab p = 0.123; WT control IgG vs zQ175 C1q Blk Ab p = 0.123; zQ175 control IgG vs WT C1q Blk Ab p = 0.005; zQ175 control IgG vs zQ175 C1q Blk Ab p = 0.005; WT C1q Blk Ab vs zQ175 C1q Blk Ab p = >0.999 via Sidak’s multiple comparisons test. **(e)** Weight changes in female mice treated with the C1q function blocking antibody or a control IgG, n = 7 WT with Ctrl IgG (3 F and 4 M), n = 5 zQ175 with Ctrl IgG (3 F and 2 M), n = 7 WT with C1q Blk Ab (3 F and 4 M), n = 4 zQ175 with C1q Blk Ab (2 F and 2 M). Two way anova with genotype/treatment as a source of variation p = 0.223; WT Ctrl IgG vs zQ175 Ctrl IgG p = 0.324; WT Ctrl IgG vs WT C1q Blk Ab p = 0.999; zQ175 Ctrl IgG vs zQ175 C1q Blk Ab p = 0.970; WT C1q Blk Ab vs zQ175 C1q Blk Ab p = 0.619; WT Ctrl IgG vs zQ175 C1q Blk Ab p = 0.659; zQ175 Ctrl IgG vs WT C1q Blk Ab p = 0.294 via Sidak’s multiple comparisons test. **(f)** Weight changes in male mice treated with the C1q function blocking antibody or a control IgG, n = 4 WT Ctrl IgG (2 F and 2 M), n = 5 zQ175 Ctrl IgG (2 F and 3 M), n = 5 WT C1q Blk Ab (3 F and 2 M), n = 7zQ175 C1q Blk Ab (3 F and 4 M). Two way anova with genotype/treatment as a source of variation p = 0.959, with WT Ctrl IgG vs zQ175 Ctrl IgG p = 0.995; WT Ctrl IgG vs WT C1q Blk Ab p = 0.989; WT Ctrl IgG vs zQ175 C1q Blk Ab p = 0.999; zQ175 Ctrl IgG vs zQ175 C1q Blk Ab p = 0.979; WT C1q Blk Ab vs zQ175 C1q Blk Ab p = 0.961; zQ175 Ctrl IgG vs WT C1q Blk Ab p = 0.999 via Sidak’s multiple comparisons test **(g)** Bar chart showing quantification of C3 puncta in the neuropil of 5 mo WT mice treated for 1 mo with the C1q function blocking antibody or a control IgG, n = 3 mice (2 F and 1 M for both genotypes). Unpaired two-tailed t-test p = 0.177. **(h)** Bar chart showing quantification of corticostriatal synapses in the dorsolateral striatum of 5 mo WT mice treated for 1 mo with the C1q function blocking antibody or a control IgG, n = 3 mice (2 F and 1 M for both genotypes). Unpaired two-tailed t-test p = 0.287. **(i)** Cumulative distribution plot of inter-spike intervals (ISI) obtained from whole-cell voltage clamp recordings of medium spiny neuron spontaneous excitatory postsynaptic currents. Recordings were carried out in slices from 5 mo WT and zQ175 mice (Grey = WT, Black = zQ175). Bar chart shows average frequency (Hz) per cell recorded across conditions, n = 16 WT cells and 15 zQ175 cells from 5 WT and 4 zQ175 mice. Kolmogorov-Smirnov test for the cumulative distribution plot p = 0.0008. **(j)** Cumulative distribution plot of amplitude obtained from whole-cell voltage clamp recordings of medium spiny neuron spontaneous excitatory postsynaptic currents. Recordings were carried out in slices from 5 mo WT and zQ175 mice (Grey = WT, Black = zQ175). Bar chart shows average amplitude (pA) per cell recorded across conditions, n = 16 WT cells and 15 zQ175 cells from 5 WT and 4 zQ175 mice. Kolmogorov-Smirnov test for the cumulative distribution plot p = 0.0008. **(k)** Box and whisker plots (box extends from 25^th^ to 75^th^ percentiles and whiskers equal min to max) showing the mean capacitance per cell from sEPSC recordings of MSN’s in slices taken from 5 mo zQ175 mice and WT littermates, n = 16 WT cells and 15 zQ175 cells from 5 WT and 4 zQ175 mice. Unpaired two-tailed t-test, p = 0.125. **(l)** Box and whisker plots (box extends from 25^th^ to 75^th^ percentiles and whiskers equal min to max) showing the mean input resistance per cell from sEPSC recordings of MSN’s in slices taken from 5 mo zQ175 mice and WT littermates, n = 16 WT cells and 15 zQ175 cells from 5 WT and 4 zQ175 mice. Unpaired two-tailed t-test, p = 0.027. **(m)** Representative traces of sEPSCs recorded from MSNs in striatal slices from 5 mo WT mice which had been treated for 1 mo with control IgG or the C1q function blocking antibody. **(n)** Box and whisker plots (box extends from 25^th^ to 75^th^ percentiles and whiskers equal min to max) showing the mean capacitance per cell from sEPSC recordings of MSN’s in slices taken from 5 mo zQ175 mice and WT littermates treated with the C1q Blk antibody (M1) or a control IgG, n = 23 cells from 7 mice for WT Ctrl IgG; n = 17 cells from 7 mice for WT C1q Blk Ab.; n = 14 cells from 4 mice for zQ175 Ctrl IgG; and n = 23 cells from 7 mice for zQ175 C1q Blk Ab. Unpaired two-tailed t-test for WT p = 0.037; for zQ175 p = 0.091. **(o)** Box and whisker plots (box extends from 25^th^ to 75^th^ percentiles and whiskers equal min to max)showing the mean input resistance per cell from sEPSC recordings of MSN’s in slices taken from 5 mo zQ175 mice and WT littermates treated with the C1q Blk antibody (M1) or a control IgG, n = 23 cells from 7 mice for WT Ctrl IgG; n = 17 cells from 7 mice for WT C1q Blk Ab.; n = 14 cells from 4 mice for zQ175 Ctrl IgG; and n = 23 cells from 7 mice for zQ175 C1q Blk Ab. Unpaired two-tailed t-test for WT p = 0.340; for zQ175 p = 0.811. **(p)** Representative confocal images of the dorsal striatum of 7 mo zQ175 mice injected IP with 20 mg/kg of FITC conjugated C1q function blocking antibody, or unconjugated blocking antibody 24 h prior to sacrifice. Scale bar = 10 μm. Note that only in mice treated with FITC conjugated C1q function blocking antibody is there evidence of punctate staining in the neuropil, n = 3 7 mo zQ175 mice treated with 20 mg/kg of FITC conjugated C1q function blocking antibody and n = 3 7 mo zQ175 mice treated with 20 mg/kg of unconjugated C1q function blocking antibody (2 F and 1 M for both conditions). For bar charts, bars depict the mean and all error bars represent SEM. Stars depict level of significance with *=p < 0.05, **p = <0.01 and ***p < 0.001. Source data

To reduce complement deposition, we injected a monoclonal C1q function-blocking antibody, also referred to as ANX-M1, intraperitoneally (IP) into 4-month-old zQ175 mice and WT littermates over a 1-month period (Fig. 5a). ANX-M1 binds to an epitope in the globular head domains of C1q, preventing C1q binding to its substrates and downstream activation of the proteases C1r and C1s (refs. ^112–114^), which results in suppression of the classical complement cascade activation and reduced cleavage and subsequent deposition of C3 (refs. ^30,34,115^). ELISA assays confirmed that treated mice had high levels of ANX-M1 and significantly less unbound C1q in their serum relative to mice treated with a control IgG (Extended Data Fig. 8c,d). FITC-tagged versions of ANX-M1 could also be detected in the neuropil of these mice, demonstrating that it can cross the blood–brain barrier (Extended Data Fig. 8p), a finding that is consistent with other studies in which rodents have received peripheral administration of antibodies^116–118^.

Treatment with the C1q function-blocking antibody reduced complement component C3 deposition in the dorsolateral striatum of zQ175 mice by approximately 60% and increased the number of corticostriatal synapses by 70% relative to treatment with a control IgG (Fig. 5b,c and Extended Data Fig. 8a,b), but it had no effect on baseline complement deposition or corticostriatal synapse numbers in WT mice (Extended Data Fig. 8g,h). It was also well tolerated, with no change in body weight during the study (Extended Data Fig. 8e,f).

To test whether these synapses were functional, we performed ex vivo electrophysiology using striatal brain slices from mice treated with the C1q function-blocking antibody or the control IgG. In line with the reduced number of corticostriatal synapses that we observed at 3 months and in keeping with what others have reported at older ages with this HD line^72,74,77,78^, untreated 5-month-old zQ175 mice showed a reduction in spontaneous excitatory postsynaptic current (sEPSC) frequency, reflecting a decline in either the presynaptic release probability or the number of presynaptic inputs onto the cell in the zQ175 mice when compared to their WT littermates (Extended Data Fig. 8i). We also saw the expected increase in input resistance, a membrane property thought to reflect a state in which the cell responds more significantly to a given stimulus and subtle reductions in amplitude that reflect changes in the density of postsynaptic receptors at an individual synapse (Extended Data Fig. 8j–l).

Treatment with the C1q function-blocking antibody significantly increased the sEPSC frequency, shifting the cumulative probability distribution plot curves for both zQ175 (*P* < 0.0002) and WT (*P* < 0.0008) relative to that seen in mice treated with control IgG. These data demonstrate that cells from mice treated with the blocking antibody had a greater number of presynaptic inputs or a greater release probability (Fig. 5d,e and Extended Data Fig. 8m). In contrast, no treatment-induced changes were observed in amplitude response or intrinsic membrane properties of these cells, such as capacitance and input resistance (Fig. 5f and Extended Data Fig. 8n,o). Taken together, these results demonstrate that blocking complement activity in zQ175 mice leads to more functional excitatory synapses on MSNs and increased excitatory input to the striatum, thereby showing that strategies that target complement in this way can reduce loss of corticostriatal synapses in HD mice.

### Development of early cognitive deficits is prevented by genetic ablation of microglial CR3

Cognitive deficits are a key clinical hallmark of HD, and impaired performance in tasks related to visual discrimination and cognitive flexibility are some of the earliest quantifiable changes observed in premanifest HD patients, occurring up to 20 years before predicted onset of manifest disease and in the absence of detectable motor phenotypes^119–121^. Impairments in learning and memory tasks have also been observed in zQ175 mice, but these assessments have been carried out only in older mice after development of striatal atrophy and other pathological hallmarks^17,71–73,122–125^.

To determine whether cognitive deficits can be observed at earlier stages of disease progression, at timepoints corresponding to the loss of corticostriatal synapses, we used an operant touchscreen platform to monitor performance in a task of visual object discrimination, in which mice had to learn which image presentation was associated with dispensing of a liquid reward. Assessment of cognitive flexibility was then performed by reversing the object display associated with reward such that the previously incorrect choice now yielded a reward response and vice versa. Four-month-old zQ175 mice displayed a significantly impaired performance in visual discrimination learning compared to WT littermates, but they were eventually able to achieve a threshold of 70% correct choice over 60 trial sessions on three consecutive trial days (Fig. 5i(i)). When the reward association was subsequently reversed, it again took the zQ175 mice much longer to reach a 70% correct choice threshold, indicating an impairment in cognitive flexibility (Fig. 5j(i)). These deficits were unlikely to be driven by differences in visual acuity, motor performance or motivation to complete the task, as optomotor measurements, the speed and number of trials completed and performance in progressive ratio tasks showed no genotype-dependent differences (Extended Data Fig. 7d–k).

To test whether strategies that block complement and microglia-mediated synapse elimination rescue the cognitive flexibility impairments observed in zQ175 mice, we also assessed the performance of zQ175 CR3KO mice. Genetic ablation of CR3 prevented the impaired rate of visual discrimination learning observed in zQ175 mice, with performance of zQ175 CR3KO mice not being significantly different to that of WT and CR3KO littermate controls (Fig. 5i (ii and iii)). Furthermore, in the reversal phase of the task, zQ175 CR3KO mice showed significantly improved performance when contrasted with zQ175 littermates, taking 240 fewer trials to achieve the 70% correct threshold (Fig. 5j (ii and iii)).

**Extended Data Fig. 9:**
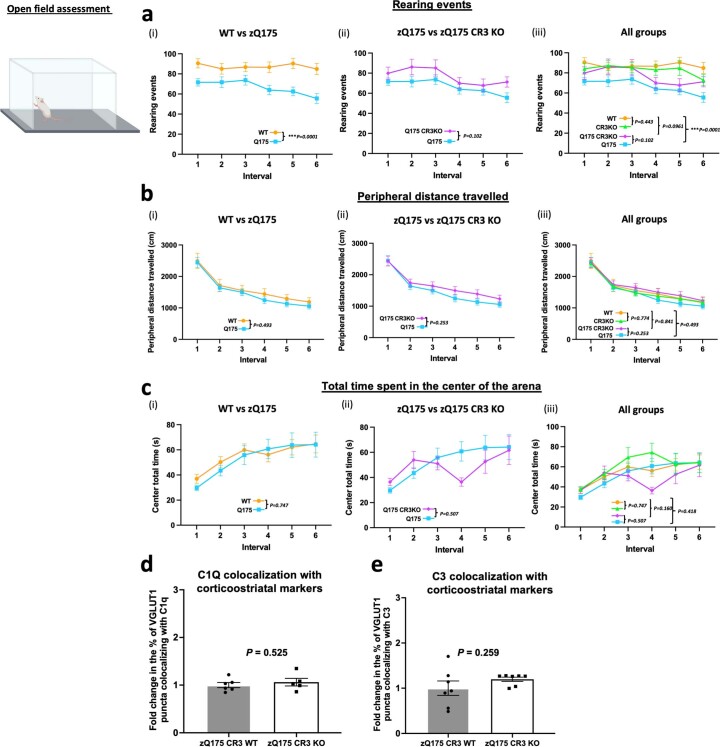
Preventing microglial recognition of complement opsonized structures can reduce development of impairments in some exploratory behaviors in HD mice. **(a)** The activity of 4 mo WT, zQ175, CR3 KO and zQ175 CR3 KO mice was assessed using the Kinder Scientific Smart Frame Open Field System and Motor Monitor II software. Line charts show the number of rearing events that took place over 6 5 minute intervals during a 30 minute assessment period **(i)** WT vs zQ175 mice, **(ii)** zQ175 vs zQ175 CR3 KO mice and **(iii)** all groups. Note that throughout the session WT mice carried out more rearing events than their zQ175 littermates and that this measurement of inspective and diversive exploration was not reduced in zQ175 mice which had complement receptor 3 genetically ablated, n = 17 WT mice (9 F, 8 M), 24 zQ175 mice (13 F, 11 M), 19 CR3KO (7 F, 12 M) and 15 zQ175 CR3KO (7 F, 9 M) mice. Two way anova: for WT vs zQ175 p = 0.149 for the combination of genotype x trial session as a significant source of variation and p = 0.0001 for genotype as a significant source of variation; for zQ175 vs zQ175 CR3KO p = 0.665 for the combination of genotype x interval as a significant source of variation and p = 0.102 for genotype as a significant source of variation; for WT vs zQ175 CR3KO p = 0.039 for the combination of genotype and trial as a significant source of variation and p = 0.096 for genotype as a significant source of variation; for WT vs CR3 KO p = 0.601 for the combination of genotype x trial session as a significant source of variation and p = 0.443 for genotype as a significant source of variation. **(b)** Line graphs showing the distance travelled by mice in the peripheral quadrant of a Kinder Scientific Smart Frame Open Field System **(i)** WT vs zQ175 mice, **(ii)** zQ175 vs zQ175 CR3 KO mice and **(iii)** all groups, n = 16 WT mice (8 F, 8 M), 24 zQ175 mice, 19 CR3KO mice and 15 zQ175 CR3KO mice. Two way anova: for WT vs zQ175 p = 0.956 for the combination of genotype x trial session as a significant source of variation and p = 0.493 for genotype as a significant source of variation; for zQ175 vs zQ175 CR3KO p = 0.719 for the combination of genotype x trial session as a significant source of variation and p = 0.253 for genotype as a significant source of variation; for WT vs zQ175 CR3KO p = 0.900 for the combination of genotype and trial as a significant source of variation and p = 0.841 for genotype as a significant source of variation; for WT vs CR3 KO p = 0.984 for the combination of genotype x trial session as a significant source of variation and p = 0.774 for genotype as a significant source of variation. For bar charts, bars depict the mean and all error bars represent SEM. Stars depict level of significance with *=p < 0.05, **p = <0.01 and ***p < 0.0001. **(c)** Line graphs showing the total time spent by mice in the central quadrant of a Kinder Scientific Smart Frame Open Field System **(i)** WT vs zQ175 mice, **(ii)** zQ175 vs zQ175 CR3 KO mice and **(iii)** all groups, n = 16 WT mice, 24 zQ175 mice, 19 CR3KO mice and 15 zQ175 CR3KO mice. Two way anova: for WT vs zQ175 p = 0.914 for the combination of genotype x trial session as a significant source of variation and p = 0.747 for genotype as a significant source of variation; for zQ175 vs zQ175 CR3KO p = 0.146 for the combination of genotype x trial session as a significant source of variation and p = 0.507 for genotype as a significant source of variation; for WT vs zQ175 CR3KO p = 0.443 for the combination of genotype and trial as a significant source of variation and p = 0.160 for genotype as a significant source of variation; for WT vs CR3 KO p = 0.631 for the combination of genotype x trial session as a significant source of variation and p = 0.418 for genotype as a significant source of variation. (**d**) Bar chart showing quantification of the percentage of VGLUT1 puncta colocalizing with C1q in the dorsolateral striatum of zQ175 and zQ175 CR3 KO littermates n = 6 zQ175 (3 F,3 M) and 5 zQ175 CR3 KO (3 F,2 M). Unpaired two-tailed t-test p = 0.525. (**e**) Bar chart showing quantification of the percentage of VGLUT1 puncta colocalizing with C3 in the dorsolateral striatum of zQ175 and zQ175 CR3 KO littermates n = 7 zQ175 and 7 zQ175 CR3 KO (3 F and 4 M for both genotypes). Unpaired two-tailed t-test p = 0.259. All error bars represent SEM. Stars depict level of significance with *=p < 0.05, **p = <0.01 and ***p < 0.001. Source data

Although we observed no impairments in motor performance metrics in the touchscreen paradigm, we wanted to see whether subtle changes in exploratory behavior could be observed in 4-month-old zQ175 mice and whether these were also rescued in the absence of CR3. To achieve this, we performed open field assessments in the dark phase of the light cycle and saw that, as expected, the total distance traveled was consistent across the genotypes (Extended Data Fig. 9b,c) In contrast, the number of rearing events, which can represent both inspective and diversive exploration of an environment, were reduced. Notably, this phenotype was rescued in mice in which CR3 was ablated, with zQ175 CR3KO mice showing similar performance to WT littermates (Extended Data Fig. 9a).

Taken together, these results show that zQ175 mice display deficits related to visual processing and cognitive flexibility as well as subtle changes in exploratory behavior at 4 months of age at a timepoint where we also observed loss of corticostriatal synapses. Notably, strategies that reduce complement-mediated synaptic elimination prevent the appearance of these deficits and return performance to WT levels.

### Complement protein levels in the CSF are correlated with measures of disease burden in premanifest HD patients

CSF is a unique biological fluid containing proteins with enriched expression in brain and spinal cord^126–129^. Modifications to its proteome reflect transcriptional changes in disease-associated regions of the HD brain^130^, and, importantly, it is possible to safely obtain CSF from patients before the manifestation of clinical symptoms, enabling the observation of early pathological changes^131,132^. To determine if changes in complement protein levels or activity can be detected in the CSF of premanifest HD patients and, if so, whether they are associated with established measures of disease burden, we developed assays to measure complement cascade proteins and their activation fragments (Extended Data Fig. 1k–m).

**Fig. 6:**
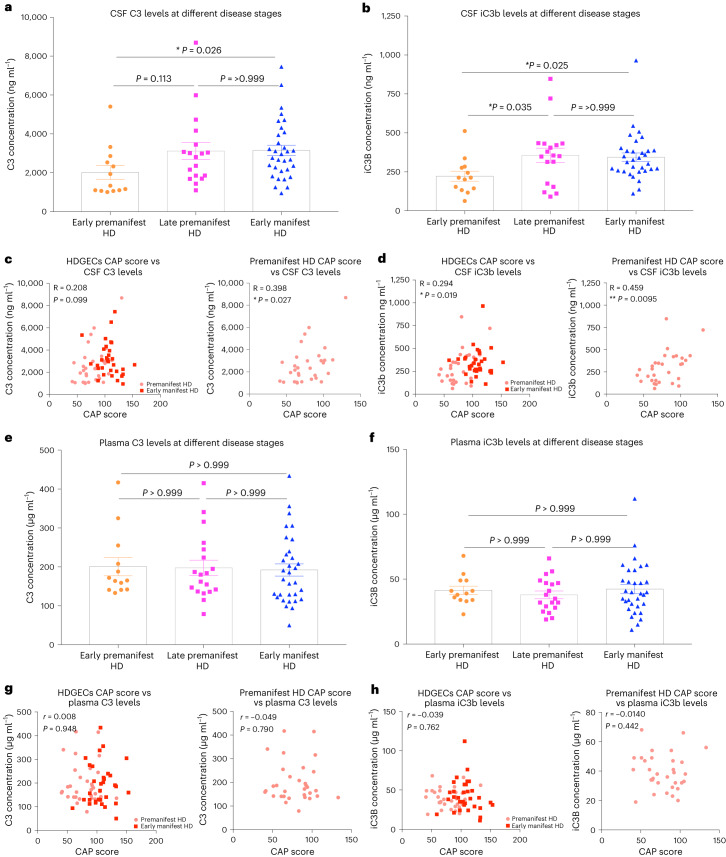
Association between disease stage and complement component levels in the CSF and plasma of patients with HD. **a**, Quantification of the concentration of complement component C3 in CSF samples from early premanifest HD patients, late premanifest HD patients and early manifest HD patients (see Methods for details) recruited into the HDClarity study. Each dot represents a sample from a separate individual, and the bar denotes the mean for each group—*n* = 13 early premanifest HD, *n* = 18 late premanifest HD and *n* = 32 early manifest HD. Kruskal–Wallis test (non-parametric ANOVA) *P* = 0.028 with early premanifest versus late premanifest HD *P* = 0.113, late premanifest versus early manifest HD *P* = >0.999 and early premanifest versus early manifest HD *P* = 0.026 via Dunn’s multiple comparison test. **b**, Quantification of the concentration of iC3b, the cleaved component of C3 that is formed after activation of the complement cascade, in the same CSF samples assayed in **a**. Kruskal–Wallis test (non-parametric ANOVA) *P* = 0.017 with early premanifest HD versus late premanifest HD *P* = 0.035, late premanifest HD versus early manifest HD *P* > 0.999 and early premanifest HD versus early manifest HD *P* = 0.025. **c**, Graphs showing association between CAP score and CSF C3 concentration for all samples from HDGECs as well as those just from premanifest HD patients recruited into the HDClarity study, *n* = 63 HDGECs and *n* = 31 premanifest HD. Spearman *r* for HDGECs *P* = 0.099; for premanifest HD *P* = 0.027. **d**, Graphs showing association between CAP score and CSF iC3b concentration for all samples from HDGECs as well as those just from premanifest HD patients recruited into the HDClarity study, *n* = 63 HDGECs and *n* = 31 premanifest HD. Spearman *r* for HDGECs *P* = 0.019; for premanifest HD *P* = 0.0095. **e**, Bar chart showing the concentration of complement component C3 in plasma samples from early premanifest HD patients, late premanifest HD patients and early manifest HD patients (see Methods for inclusion criteria) recruited into the HDClarity study, *n* = 13 early premanifest HD, *n* = 19 late premanifest HD and *n* = 32 early manifest HD. Kruskal–Wallis test (non-parametric ANOVA) *P* = 0.797 with early premanifest versus late premanifest HD *P* > 0.999, late premanifest versus early manifest HD *P* > 0.999 and early premanifest versus early manifest HD *P* > 0.999 via Dunn’s multiple comparison test. **f**, Bar chart showing the concentration of iC3b, the cleaved component of C3 formed after activation of the complement cascade, in the same plasma samples assayed in **e**. Kruskal–Wallis test (non-parametric ANOVA) *P* = 0.609 with early premanifest HD versus late premanifest HD *P* > 0.999, late premanifest HD versus early manifest HD *P* > 0.999 and early premanifest HD versus early manifest HD *P* > 0.999. **g**, Graphs showing the association between CAP score and plasma C3 concentration for all samples from HDGECs as well as those just from premanifest HD patients recruited into the HDClarity study, *n* = 64 HDGECs and *n* = 32 premanifest HD. Spearman *r* for HDGECs *P* = 0.948; for premanifest HD *P* = 0.790. **h**, Graphs showing association between CAP score and plasma iC3b concentration for all samples from HDGECs as well as those just from premanifest HD patients recruited into the HDClarity study, *n* = 64 HDGECs and *n* = 32 premanifest HD. Spearman *r* for HDGECs *P* = 0.762; for premanifest HD *P* = 0.442. For bar charts, bars depict the mean. All error bars represent s.e.m. *P < 0.05, **P < 0.01 and ***P < 0.001. Source data

Analysis of CSF samples from the HDClarity study group with ELISA assays that detect C3 and iC3b showed that both proteins were altered with disease stage (as defined by both clinical assessment and burden of pathology score) in patients with HD (Fig. 6a,b). Levels of C3 and iC3b were significantly increased in patients with early manifest HD relative to those with early premanifest HD. Notably, for iC3b, this increase was also seen between early premanifest HD patients and late premanifest HD patients, a transition associated with reductions in brain and striatal volume, white matter microstructure damage, reduced functional activity of the basal ganglia while performing tasks and multiple subtle differences in motor and cognitive performance as well as differences in neuropsychiatric assessments^1,26,131,133,134^. This demonstrates that changes in abundance of iC3b occur before onset of motor symptoms.

**Extended Data Fig. 10:**
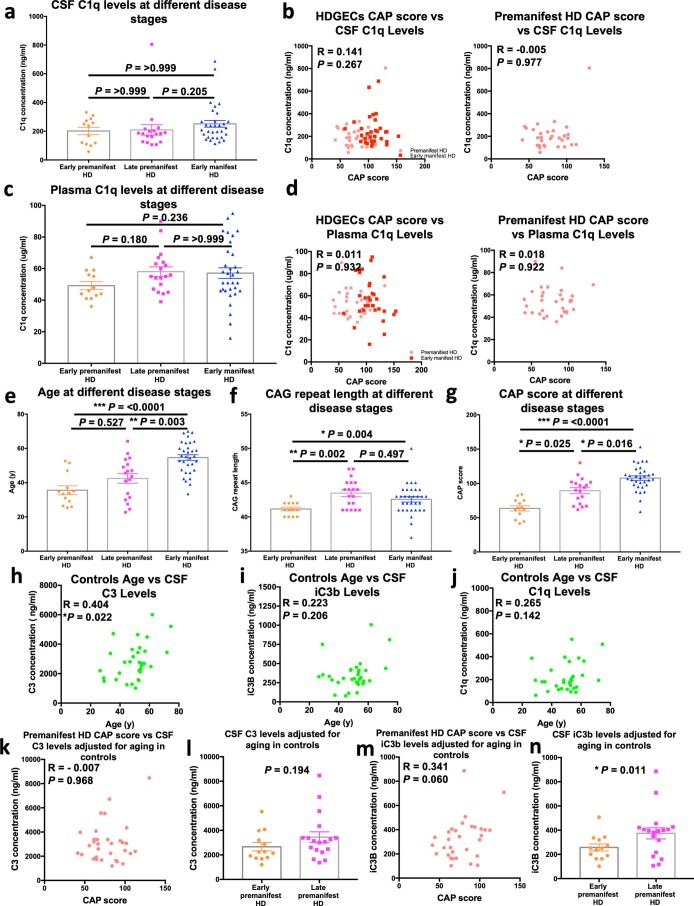
Association between disease stage and complement component levels and activity in the CSF and plasma of Huntington’s disease patients. **(a)** Bar chart showing the concentration of complement component C1q in CSF samples from early premanifest HD patients (see Methods for inclusion criteria), late premanifest HD patients (see Methods for inclusion criteria) and early manifest HD patients (see Methods for inclusion criteria) recruited into the HDClarity study. Each dot represents a sample from a separate individual and the bar denotes the mean for each group n = 13 early premanifest HD, n = 18 late premanifest HD and n = 32 early manifest HD. Kurkasl-Wallis test (non-parametric ANOVA) p = 0.175 with early premanifest versus late premanifest HD p = >0.999, late premanifest versus early manifest HD p = 0.205 and early premanifest versus early manifest HD p = >0.999 via Dunn’s multiple comparison test. **(b)** Graphs showing the association between CAP score and CSF C1q concentration for all samples from Huntington’s Disease gene expansion carriers (HDGECs) as well as those just from premanifest HD patients recruited into the HDClarity study. Each dot represents a sample from a separate individual n = 63 HDGEC’s and n = 31 premanifest HD. Spearman r for HDGEC’s p = 0.267; for premanifest HD p = 0.977. **(c)** Bar chart showing the concentration of complement component C1q in plasma samples from early premanifest HD patients (see Methods for inclusion criteria), late premanifest HD patients (see Methods for inclusion criteria) and early manifest HD patients (see Methods for inclusion criteria) recruited into the HDClarity study. Each dot represents a sample from a separate individual and the bar denotes the mean for each group n = 13 early premanifest HD, n = 19 late premanifest HD and n = 32 early manifest HD. Kurkasl-Wallis test (non-parametric ANOVA) p = 0.133 with early premanifest versus late premanifest HD p = 0.180, late premanifest versus early manifest HD p = >0.999 and early premanifest versus early manifest HD p = 0.236 via Dunn’s multiple comparison test. **(d)** Graphs showing the association between CAP score and plasma C1q concentration for all samples from Huntington’s Disease gene expansion carriers (HDGECs) as well as those just from premanifest HD patients recruited into the HDClarity study. Each dot represents a sample from a separate individual n = 64 HDGEC’s and n = 32 premanifest HD. Spearman r for HDGEC’s p = 0.932; for premanifest HD p = 0.922. **(e)** Bar chart showing the ages of the early premanifest HD patients (see Methods for inclusion criteria), late premanifest HD patients (see Methods for inclusion criteria) and early manifest HD patients (see Methods for inclusion criteria) from the HDClarity study whose samples were assessed in this study. Each dot represents a sample from a separate individual and the bar denotes the mean for each group n = 13 early premanifest HD, n = 19 late premanifest HD and n = 32 early manifest HD. Kurkasl-Wallis test (non-parametric ANOVA) p = 0.000009 with early premanifest versus late premanifest HD p = 0.527, late premanifest versus early manifest HD p = 0.003 and early premanifest versus early manifest HD p = 0.00003 via Dunn’s multiple comparison test. **(f)** Bar chart showing the ‘high’ CAG repeat number of the early premanifest HD patients (see Methods for inclusion criteria), late premanifest HD patients (see Methods for inclusion criteria) and early manifest HD patients (see Methods for inclusion criteria) from the HDClarity study whose samples were assessed in this study. Each dot represents a sample from a separate individual and the bar denotes the mean for each group n = 13 early premanifest HD, n = 19 late premanifest HD and n = 32 early manifest HD. Kurkasl-Wallis test (non-parametric ANOVA) p = 0.003 with early premanifest versus late premanifest HD p = 0.002, late-premanifest versus early manifest HD p = 0.497 and early premanifest versus early manifest HD p = 0.004 via Dunn’s multiple comparison test. **(g)** Bar chart showing the CAP score of the early premanifest HD patients (see Methods for inclusion criteria), late premanifest HD patients (see Methods for inclusion criteria) and early manifest HD patients (see Methods for inclusion criteria) from the HDClarity study whose samples were assessed in this study. Each dot represents a sample from a separate individual and the bar denotes the mean for each group n = 13 early premanifest HD, n = 18 late premanifest HD and n = 32 early manifest HD. Kurkasl-Wallis test (non-parametric ANOVA) p = 0.0000002 with early premanifest versus late premanifest HD p = 0.025, late premanifest versus early manifest HD p = 0.016 and early premanifest versus early manifest HD p = 0.0000002 via Dunn’s multiple comparison test. **(h)** Graph showing the association between age and CSF C3 concentration for control (clinically normal) individuals recruited into the HDClarity study. Each dot represents a sample from a separate individual n = 32. Spearman r p = 0.022. **(i)** Graph showing the association between age and CSF iC3b concentration for control (clinically normal) individuals recruited into the HDClarity study. Each dot represents a sample from a separate individual n = 32. Spearman r p = 0.206. (**J**) Graph showing the association between age and CSF C1q concentration for control (clinically normal) individuals recruited into the HDClarity study. Each dot represents a sample from a separate individual n = 32. Spearman r p = 0.142. **(k)** Graph showing the association between CAP score and CSF C3 concentration (after adjustment for the effects of aging in controls) in samples from premanifest HD patients recruited into the HDClarity study. Each dot represents a sample from a separate individual n = 31 premanifest HD. Spearman r p = 0.968. **(l)** Bar chart showing the CSF C3 concentration (after adjustment for the effects of aging in controls) in samples from early premanifest HD and late premanifest HD patients. Each dot represents a sample from a separate individual and bars equal the mean for each group n = 13 early premanifest HD and n = 18 late premanifest HD. Kolmogorov-Smirnov test p = 0.194. **(m)** Graph showing the association between CAP score and CSF iC3b concentration (after adjustment for the effects of aging in controls) in samples from premanifest HD patients recruited into the HDClarity study. Each dot represents a sample from a separate individual n = 31 premanifest HD. Spearman r p = 0.060. **(n)** Bar chart showing the CSF iC3b concentration (after adjustment for aging in controls) in samples from early premanifest HD and late premanifest HD patients. Each dot represents a sample from a separate individual and bars equal the mean for each group n = 13 early premanifest HD and n = 18 late premanifest HD. Kolmogorov-Smirnov test p = 0.011. For bar charts, bars depict the mean. All error bars represent SEM. Stars depict level of significance with *=p < 0.05, **p = <0.01 and ***p < 0.001. Source data

These findings were further confirmed by looking at the association between CSF concentrations of C3 and iC3b and the CAG Age Product (CAP) score of patients with HD. CAP score is a measure of disease burden derived from both the length of the individuals’ CAG expansions and their current age. Changes in this metric correlate with reductions in striatal volume, damage to white matter microstructure and, most importantly, development of motor impairments^1,135–142^ (Extended Data Fig. 10e–g). For C3 and iC3b, there was a significant positive correlation between their CSF concentrations and CAP score in premanifest HD patients—a relationship that was maintained for iC3b after incorporating samples from early manifest HD patients (Fig. 6c,d).

Both age and sex are potentially confounding variables. In line with previous studies, Spearman rank correlation models showed that CSF C3 levels were elevated with aging in control individuals (Extended Data Fig. 10h)^143–146^. iC3b levels, in contrast, were not (Extended Data Fig. 10i). To control for normal aging in our study, we adjusted measured CSF concentrations of C3 and iC3b using a formula that incorporates the regression coefficient of aging in control individuals and the mean age of the control population in our sample cohort. Although the significant association with CAP score in premanifest HD patients did not survive this adjustment, the difference in CSF iC3b levels between early and late premanifest HD patients was maintained (Extended Data Fig. 10k–n). However, the limited age range of the control samples and the fact that younger ages were substantially underrepresented (Supplementary Table 1) means that further analysis of larger and more balanced cohorts of CSF samples from control individuals will be required to gain a better understanding of the effect of normal aging on CSF levels of complement proteins. Gender also affects CSF C3 levels, with males having significantly more C3 in the HD patient samples; however, given the equal distribution of genders in all three patient groups, this did not alter the complement protein changes observed with disease stage (Supplementary Fig. 2a–c).

A positive correlation between CSF iC3b levels and CAP score was also observed in a separate smaller cohort of CSF samples collected at the University of Washington (Supplementary Fig. 3b), further demonstrating that iC3b increases with HD progression. Longitudinal measurements from three premanifest HD patients in this cohort, carried out 1.5 years after the first collection (Supplementary Table 1), all showed increased iC3b levels (Supplementary Fig. 3g–i), providing additional evidence that CSF iC3b concentration increases with disease progression. In contrast, C3 levels did not change in this CSF cohort, likely due to the smaller sample size and weaker relationship between CAP score and C3 levels (Fig. 6c and Supplementary Fig. 3a).

C3 and iC3b concentrations were unaltered with disease stage in paired plasma samples from the same patients, and there was no correlation between plasma and CSF levels, demonstrating that complement changes in the CSF are localized and not mirrored by systemic changes in other body fluids (Fig. 6e,f and Supplementary Fig. 2g–i). Plasma levels of C3 and iC3b also showed no correlation with CAP score and no significant difference in levels between genders (Fig. 6g,h and Supplementary Fig. 2d–f). This finding differs from what has been reported for downstream complement components such as C7, which have been found to be elevated in plasma samples from manifest HD patients relative to those from patients in the premanifest state^147,148^.

Interestingly, despite C1Q being the initiating protein in the classical complement cascade and, thus, acting to promote activation/cleavage of C3 to iC3b, its levels were not altered with disease stage in the CSF of patients with HD, and it showed no association with CAP score (Extended Data Fig. 10a,b). C1Q levels also showed no significant correlation with age in control individuals, were not different in males and females and were unaltered with disease stage in plasma samples (Extended Data Fig. 10c,d,j and Supplementary Fig. 2c,f).

Taken together, these results show that there are early changes in complement biology occurring specifically in the CSF of premanifest HD patients. Further work will be required to determine the factors prompting these changes and whether they correlate with clinical measures of disease progression as well as other known imaging and fluid markers.

## Discussion

Our study provides strong preclinical evidence that strategies to block complement-mediated microglial engulfment of synapses in HD may provide a therapeutic benefit in preventing loss of corticostriatal synaptic connectivity and reducing cognitive behavioral deficits. We found that, in two well-established HD mouse models expressing full-length mHTT, corticostriatal synapses are selectively lost at a very early disease stage, before onset of robust motor and cognitive deficits. We provide multiple lines of evidence that complement and microglia mediate this corticostriatal synaptic loss, with C1q and C3 specifically associating with this synaptic population early in disease progression and microglia engulfing more cortical inputs. Inhibition of the classical complement pathway with a therapeutically relevant C1q function-blocking antibody and genetic ablation of complement receptor CR3, a gene almost exclusively expressed by microglia in the brain, both reduce early loss of corticostriatal synapses in HD mouse models. In addition, ablation of microglial CR3 (ref. ^91^) prevented development of deficits in visual discrimination learning and rescued impairments in cognitive flexibility. Altough recent studies have demonstrated that microglia and complement coordinate to eliminate synapses in certain pathological contexts and also that specific complement proteins can be targeted to improve aspects of behavior and neuropathology in Alzheimerʼs disease models^30–32,34,36,149–151^, our study is the first, to our knowledge, to show that (1) these mechanisms can selectively target specific synapses in a disease-relevant neuronal circuit and (2) that strategies that block this process and preserve this synaptic population can also prevent the development of cognitive deficits.

An important question raised by these findings is what mechanisms mediate selective association of complement proteins with corticostriatal synapses. Cortical and thalamic inputs synapse preferentially onto different parts of striatal neuron dendrites^67–69^, and the receptor subunit composition and electrical properties of these two synaptic populations are distinct^152^. These structural differences may make thalamostriatal synapses more stable and less accessible for microglial engulfment. Equally, the different molecular compositions and pattern of electrical activity in these two synaptic populations could affect their levels of putative complement regulators, such as CSMD1 and CLU, both of which have been found to be downregulated at the protein level in mouse models of HD^17^. CD47, a protective signal that inhibits microglial engulfment and whose localization at synapses is regulated by levels of neuronal activity^17,153^, may also be differentially distributed between these two synaptic populations. Finally, differences in astrocyte proximity to synaptic populations at early stages of the disease may also play a role in corticostriatal vulnerability. A recent study using a FRET-based proximity detector found reduced astrocyte association with corticostriatal inputs, but not thalamostriatal inputs, in HD mice before synapse loss occurs^87^. Future studies to further delineate whether specific populations of corticostriatal synapses, such as those arising from intratelencephalic neuron or pyramidal neuron inputs, or those forming connections onto D1 or D2 MSNs, have a greater propensity for complement association and elimination at early stages of the disease will also be valuable in this regard.

mHTT is expressed ubiquitously but elicits selective pathology in specific brain regions and synaptic circuits and causes cell-autonomous and non-cell-autonomous pathological effects^10,19,49,106–109,111^. Our data show that mHTT expression in both presynaptic cortical and postsynaptic striatal neurons is specifically required to trigger complement and microglia-mediated synaptic elimination through non-cell-autonomous mechanisms. Furthermore, mHTT expression in microglia alone is not sufficient to induce this process. Whether mHTT expression in microglia acts to potentiate this pathology, however, remains an open question. A recent study found that a 50% reduction of mHTT in microglia normalizes expression of inflammatory cytokines but has no effect on deterioration of motor performance or brain atrophy in the BACHD model^108^.

Although our data show that neuronal mHTT expression initiates complement-mediated synaptic elimination, the mechanisms underlying this process remain to be determined. The corticostriatal pathway exhibits aberrant neuronal activity early in disease progression, before onset of motor or cognitive deficits^21,22^, so it could be that mHTT expression in neurons leads to changes in corticostriatal synaptic transmission and neuronal firing that are detected by microglia. During normal developmental critical periods, microglia engulf retinal inputs in the visual thalamus in an activity-dependent manner^95^. Thus, we could hypothesize that, in disease contexts, aberrant neuronal activity, arising from mHTT expression in cortical and striatal neurons^19,154^, promotes deposition of a pro-engulfment molecular cue, such as complement, or removal or redistribution of protective molecules, such as CD47, which then prompts microglial engulfment and removal of the synapse^95,153^. Understanding the link between mHTT-induced abnormalities in neuronal interactions and complement-mediated microglial engulfment will be crucial in shedding light on upstream drivers of this synaptic elimination mechanism.

We found that blocking complement-mediated microglial engulfment of synapses in HD mouse models prevented synaptic loss and inhibited the appearance of early deficits in visual discrimination and cognitive flexibility tasks. These results are consistent with previous studies where less specific methods were employed to demonstrate a selective role for this brain region in the performance of similar tasks^155^ as well as previous work showing that depleting microglia in HD mice can improve performance in some motor and cognitive tests^156^. However, it remains to be determined whether preservation of synapses has an impact on disease progression. It will, thus, be important to determine whether the synapses that remain after intervention are still functional and if there is a critical period after which it is not possible to intervene. Studies on developmental synaptic pruning have shown that complement knockout (KO) mice have excessive numbers of functional synapses in the retinogeniculate system^157^, suggesting that preserved corticostriatal synapses will still be able to transmit signals, and, indeed, our slice electrophysiology data show that striatal neurons in HD mice treated with the C1q function-blocking antibody have a greater number of excitatory inputs. However, a more in-depth analysis will be required to determine if these synapses still respond to different patterns of neuronal input in the same way and whether they are maintained as the disease progresses. Notwithstanding the converging evidence of synaptic and behavioral benefit from genetic and pharmacological strategies to inhibit complement-mediated synapse elimination in HD models, we are cognizant that our study is focused on relatively early stages of the disease. Thus, future investigations of the kinds described above are needed in preclinical models of HD to determine optimal therapeutic windows for intervention and to assess whether blocking a mechanism known to be important for synaptic pruning in development might have unintended adverse impacts on synaptic plasticity in the adult. The outcome of these studies will be important for translating these findings into the clinic.

We provided several lines of evidence that complement and microglia-mediated synaptic elimination mechanisms may also be operating in patients with HD using both imaging and biochemical approaches to interrogate postmortem human tissue samples. In addition, we found that, in premanifest and early manifest HD patients, C3 and iC3b levels in the CSF correlate with an established predictor of pathological severity and time to clinically defined disease onset. Taken together with a recent positron emission tomography (PET) imaging study that found specific loss of the presynaptic protein SV2A in the caudate and putamen of premanifest HD patients^158^, these results demonstrate that changes in complement biology and loss of synaptic markers occur in the central nervous system (CNS) of patients with HD before symptom onset and, thus, that the elimination mechanism that we describe may already be operating at this stage of the disease. Future longitudinal studies will be required to see whether changes in complement levels in HD patient CSF samples correlate with other aspects of clinical disease progression, such as changes in the well-established motor and cognitive tests that comprise the Unified Huntington’s Disease Rating Scale (UHDRS). If they do, they could be used alongside other fluid and imaging markers^132,159–161^ as determinates of disease state, enabling progression to be monitored and potentially predicting development of clinical symptoms. Furthermore, they would serve as important biomarkers of target engagement and pharmacodynamic impact, aiding in development of anti-complement therapies in the clinic in patients with HD. To this point, a phase 2 study of an initial anti-C1q therapy (ANX005) is currently ongoing in patients with HD (ClinicalTrials.gov NCT04514367).

To our knowledge, our study provides the first in vivo evidence to validate targeting complement component C1q and complement receptor CR3 to prevent synapse loss and improve cognitive function in HD mouse models, and it strengthens the rationale for developing complement-related therapeutics, including the anti-C1q antibody ANX005. Future work is needed to determine the extent to which these strategies will be successful in patients with HD.

## Methods

### Experimental procedures

#### Mice

zQ175 heterozygous mice^71,72^, in which a fragment extending upstream of *htt* exon 1 has been replaced with the human *Htt* sequence containing an expanded number of repeats of the CAG tract, and WT littermates, were either bred in-house or obtained from The Jackson Laboratory (JAX) (JAX stock no. 027410). BACHD (JAX stock no. 008197), BR, BE, BER and WT littermates^19,70^ were obtained from JAX and from the laboratory of William Yang at the University of California, Los Angeles (UCLA). C57BL/6J mice were obtained from JAX (JAX stock no. 000664), and CR3 KO mice (ref. ^162^; JAX stock no. 003991) were bred in-house and crossed with zQ175 mice to generate zQ175 heterozygous CR3 KO mice. Homer GFP mice^101^ were obtained from the laboratory of Shigeo Okabe at the Tokyo Medical and Dental University, bred in-house and crossed with zQ175 mice to generate zQ175 heterozygous Homer GFP mice. The zQ175, CR3 KO and Homer GFP mice were all on a C57BL/6J congenic background, whereas the BACHD mice were on an FVB/NJ congenic background. We used P1 or P2 and P300 mice for motor cortex injections and P120 mice for IP injections. For all other experiments, mice were used at the ages specified in the procedures listed below and displayed in relevant figure panels and legends. A mixture of male and female mice was employed in all experiments, and the exact distribution is stated in Supplementary Table 3. Animals were group housed in Optimice cages and maintained in the temperature range and environmental conditions recommended by the Association for Assessment and Accreditation of Laboratory Animal Care (AAALAC). A 12-h light/12-h dark cycle was implemented, and the temperature inside the cage was maintained between 18 °C and 23 °C with humidity between 40% and 60%. All experiments were approved by the Institutional Animal Care and Use Committee of Boston Children’s Hospital and UCLA in accordance with National Institutes of Health (NIH) guidelines for the humane treatment of animals.

#### Mouse genotyping

DNA was extracted from ear clips using the HotSHOT method described in the Quick DNA purification section of the JAX website (https://www.jax.org/jax-mice-and-services/customer-support/technical-support/genotyping-resources/dna-isolation-protocols; ref. ^163^) or from tail clips using the phenol/chloroform method described on the JAX website (https://www.jax.org/jax-mice-and-services/customer-support/technical-support/genotyping-resources/dna-isolation-protocolsz). zQ175 heterozygous mice were genotyped for the CAG expansion using the protocol provided by JAX and developed by Laragen, Inc. and for the Neo cassette using the protocol provided by JAX. CR3KO and Homer GFP mice were genotyped using the below protocols for reagent setup and thermocycling. Primer details can also be found in the Key Resources Table. In all cases, the PCR product was visualized on 2% agarose gels with ethidium bromide.

*CR3 genotyping*. Details of the reagent setup and thermocycling conditions can be found in Supplementary Table 4 alongside the expected band sizes.

*Homer GFP genotyping*. Details of the reagent setup and thermocycling conditions can be found in Supplementary Table 4 alongside the expected band sizes.

#### CSF and plasma samples (clinical details for all samples can be found in Supplementary Table 1)

HDClarity is a multi-site CSF collection initiative designed to facilitate therapeutic development for HD. It incorporates both longitudinal and cross-sectional sampling (HDClarity clinical protocol at https://hdclarity.net/). Before sample collection, appropriate ethical approval, including ethical review board (ERB) and institutional review board (IRB) consent, was obtained from all clinical sites in accordance with the rules and regulations in those countries. All participants were required to provide informed consent before undertaking study procedures, and these informed consents were obtained by clinical site staff using approved processes.

*Criteria for subject group placement*. Healthy controls – Individuals who have no known family history of HD or have been tested for the huntingtin gene glutamine codon expansion and are not at risk.

Early premanifest HD – Individuals who do not have any clinical diagnostic motor features of HD, defined as UHDRS score <4 and who have a GAG expansion of ≥40 as well as a burden of pathology score (computed as (CAG − 35.5) × age) of <250 *.

Late premanifest HD – Individuals who do not have any clinical diagnostic motor features of HD, defined as UHDRS score <4 and who have a CAG expansion of ≥40 as well as a burden of pathology score of >250 *.

Early manifest HD – Individuals who have an UHDRS score of 4 and a CAG expansion of ≥36 and have a UHDRS total functional capacity (TFC) score between 7 and 13 inclusive.

*Stratifying premanifest HD patients into two groups on the basis of their age and number of CAG repeats has been shown to reveal differences in total brain volume, subcortical gray matter volume, cortical thickness, white matter microstructure, resting-state cerebral blood flow, functional activity of the basal ganglia while performing a time discrimination task, multiple measures of motor and cognitive performance and differences in neuropsychiatric assessments^1,26,89,131,133,164^. In addition, particularly pertinent to this study, this stratification has shown differences in the levels of a putative fluid biomarker of disease progression^132^.

The burden of pathology score used to separate early and late premanifest HD patients in this study yielded a similar division to that obtained by Tabrizi et al. and Mason et al., who divided their premanifest patients into two groups (termed PreHD-A and PreHD-B) using the group median for predicted years to onset as determined by the survival analysis formula described by Langbehn et al.^131,164,165^. In fact, only three samples were differently allocated between the early and late premanifest groups when comparing these two stratification approaches, and this did not affect the disease-stage-dependent differences in CSF levels of C3 and iC3b (data not shown). Stratifying early and late premanifest HD patients on the basis of a threshold number of years to onset of 10.8 (determined using the Langbehn formula) as employed by Byrne et al. (on the basis of the group median predicted years to onset for the sample cohort in their study) also did not affect the observed disease-stage-dependent differences in CSF levels of C3 and iC3b (data not shown)^132,161^. For our sample cohort, the median estimated years to diagnosis for early premanifest HD patients when employing the Langbehn formula was 26, which contrasts with a value of 9.84 for the late premanifest HD patients. This is very similar to that reported for the Cambridge cohort in Mason et al. (22.1 and 10.8, respectively), in which differences in subcortical volumes were observed^164^.

*Sample collection*. For CSF samples, lumbar punctures were carried out according to the guidelines stipulated in the HDClarity clinical protocol and with the approval of the relevant IRBs. In brief, patients were placed into the lateral decubitus position, and CSF was extracted from the subarachnoid space at the L3–L4 or L4–L5 interspace using a spinal needle. All samples were collected in a highly standardized manner with the collection time, method of extraction and storage all controlled (for further details, see the HDClarity clinical protocol at https://hdclarity.net/). In addition, patients underwent blood tests and clinical screening to account for any confounding variables, such as underlying infections. CSF samples were also screened for blood cell contamination of >1,000 erythrocytes per microliter and were then aliquoted and maintained at −80 °C until use (for further details, see the HDClarity clinical protocol at https://hdclarity.net/). To further examine the degree of blood contamination or ascertain whether there is any evidence of blood–brain barrier breakdown in our samples, the CSF concentration of albumin, a molecule that constitutes around 50% of the total protein in the plasma, and which has also been shown in many studies to be present at significantly lower levels in the CSF, was quantified (Supplementary Fig. 2j,k)^166–169^. Previous studies using albumin labeled with radioactive iodine showed that the blood–brain barrier has a relatively low permeability for this species relative to other plasma proteins that undergo receptor-mediated transport and that it can reflect blood–brain barrier disruption under certain experimentally defined paradigms^117,170^. We found that, for all of the HDClarity samples, CSF albumin concentrations were below the average level found in a previous study of a large patient cohort (over 1,000 individuals whose serum:CSF albumin ratio was considered normal)^171^. Notably, there was also no disease-stage-specific changes in CSF albumin levels and no correlation of albumin levels with patient CAP score either when all Huntington’s disease gene expansion carriers (HDGECs) were considered or just when samples from premanifest patients were interrogated (Supplementary Fig. 2j,k). These findings are consistent with a previous study that found no difference in the serum:CSF albumin ratio when comparing CSF samples from patients with HD and control (clinically normal) individuals^172^ and suggest that any disease-stage-associated changes in the analytes that we are assessing are unlikely to be driven by differences in the levels of blood contamination observed in these patient subject groups.

For plasma samples, venous blood collection was carried out according to the guidelines stipulated in the HDClarity clinical protocol and with the approval of the relevant IRBs. In brief, blood was drawn immediately after CSF sample collection was complete and collected in lithium heparin tubes. Tubes were then spun at 1,300*g* for 10 min at 4 °C, and the plasma supernatant was aliquoted and stored at −80 °C. If the supernatant appeared pink after spinning, implying that hemolysis had taken place, the sample was discarded.

A full list of the organizations that have approved the HDClarity study protocol is provided below:

1. United States, Colorado, Rocky Mountain Movement Disorders Center Recruiting. Englewood, Colorado, United States; Contact: Karen Ortiz, 303-867-5473, kortiz@kumarneuro.com. Principal Investigator: Rajeev Kumar, MD
2. United States, District of Columbia, Georgetown University Recruiting. Washington, District of Columbia, United States; Contact: Erin Koppel, 202-687-1525, ek875@georgetown.edu. Principal Investigator: Karen Anderson, MD
3. United States, Maryland, Johns Hopkins University Recruiting, Baltimore, Maryland, United States; Contact: Kia Ultz, 410-955-1349, kcarte23@jhmi.edu. Principal Investigator: Jee Bang, MPH, MD
4. United States, North Carolina, Wake Forest University Recruiting, Winston-Salem, North Carolina, United States; Contact: Christine O’ Neill, 336-716-8611, coneill@wakehealth.edu. Principal Investigator: Francis Walker, MD
5. United States, Tennessee, Vanderbilt University Medical Center Recruiting, Nashville, Tennessee, United States; Contact: Elizabeth Huitz, RN, 615-936-1007, elizabeth.huitz@vumc.org. Principal Investigator: Daniel Claassen, MD, MS
6. United States, Texas, University of Texas Health Science Center Recruiting, Houston, Texas, United States; Contact: Brittany Duncan, 713-486-3134, brittany.j.duncan@uth.tmc.edu. Principal Investigator: Erin Furr-Stimming, MD
7. Canada, British Columbia, University of British Columbia, The Centre for Huntingtonʼs Disease Recruiting, Vancouver, British Columbia, Canada; Contact: Mike Adurogbangba, 604-822-4872, madurogbangba@cmmt.ubc.ca. Principal Investigator: Blair Leavitt, MD, CM
8. Canada, Ontario, Centre for Movement Disorders, completed, Toronto, Ontario, Canada
9. Germany, University Hospital Ulm Recruiting, Ulm, Baden-Württemberg, Germany; Contact: Hela Jerbi, +49 731-500-63080, hela.jerbi@uniklinik-ulm.de. Principal Investigator: Jan Lewerenz, MD
10. Dresden University, not yet recruiting, Dresden, Saxony, Germany; Contact: Simone Koegler, 351- 458 2524, simone.koegler@uniklinikum-dresden.de. Principal Investigator: Bjoern Falkerburger, MD
11. St. Josef and Elisabeth Hospital Recruiting, Bochum, Germany; Contact: Barbara Kaminski, b.kaminski@klinikum-bochum.de. Principal Investigator: Carsten Saft, MD
12. University Hospital of Erlangen Recruiting, Erlangen, Germany; Contact: Pia-Marie Pryssok, 09131-85-44751, pia-marie.pryssok@uk-erlangen.de. Principal Investigator: Jürgen Winkler, MD
13. George Huntington Institute Recruiting, Münster, Germany; Contact: Svenja Aufenberg, +49-251-788-788-0, svenja.aufenberg@ghi-muenster.de. Principal Investigator: Ralf Reilmann, MD
14. Italy Fondazione I.R.C.C.S. Istituto Neurologico Carlo Besta Recruiting, Milan, Italy; Contact: Anna Castaldo (+)39 0223942519, anna.castaldo@istituto-besta.it. Principal Investigator: Caterina Mariotti, MD
15. Lega Italiana Ricera Huntington Recruiting, Rome, Italy; Contact: Consuelo Ceccarelli, consuelo.ceccarelli@lirh.it. Principal Investigator: Ferdinando Squitieri, MD
16. Poland Institute of Psychiatry and Neurology Recruiting, Warsaw, Poland; Contact: Malgorzata Dusza-Rowińska, +48 698250623, m.dusza.rowinska@gmail.com. Principal Investigator: Grzegorz Witkowski, MD, PhD
17. Spain Hospital de Sant Pau Recruiting, Barcelona, Spain; Contact: Cristina Barrionuevo, +34 649 14 23 60, cIzquierdo@santpau.cat. Principal Investigator: Jamie Kulisevsky, MD, PhD
18. United Kingdom Royal Devon & Exeter NHS Foundation Trust Recruiting, Exeter, Devon, United Kingdom; Contact: Robert Wells, 01392408181, robert.wells2@nhs.net. Principal Investigator: Tim Harrower, MBBS
19. Glasgow Clinical Research Facility Recruiting, Glasgow, Scotland, United Kingdom; Contact: Helen Bannister, 0141 232 7600, helen.bannister@ggc.scot.nhs.uk. Principal Investigator: Matthew Sheridan, MBBS
20. Birmingham Huntingtonʼs Disease Clinic Recruiting, Birmingham, West Midlands, United Kingdom; Contact: Jennifer De Souza, 0121 301 2363, jennifer.desouza@bsmhft.nhs.uk. Principal Investigator: Hugh Rickards, MB, ChB, MSc, FRCPsych, MD
21. North Bristol NHS Trust, not yet recruiting, Bristol, United Kingdom; Contact: Catherine Watkins, catherine.watkins@nbt.nhs.uk, Principal Investigator: Elizabeth Coulthard, MBBS
22. Cambridge University Hospitals NHS Foundation Trust Recruiting, Cambridge, United Kingdom; Contact: Katie Andresen, 01223 331141, kera2@cam.ac.uk. Principal Investigator: Roger Barker, MBBS, PhD
23. Cardiff University Recruiting, Cardiff, United Kingdom; Contact: Alison Johnson, 02920746394, alison.johnson@wales.nhs.uk. Principal Investigator: Anne Rosser, MD
24. Fife Health Board, Whyteman’s Brae Hospital Recruiting, Kirkcaldy, United Kingdom; Contact: Fleur Davey, 01383 623623, fleurdavey@nhs.net. Principal Investigator: Robert Thompson, MBBS
25. Leeds Teaching Hospital Trust Recruiting, Leeds, United Kingdom; Contact: Callum Schofield, 0113 39 24679, callum.schofield@nhs.net. Principal Investigator: Jeremy Cosgrove, MBBS
26. The Walton Centre NHS Foundation Trust Recruiting, Liverpool, United Kingdom; Contact: Andrea Clyne, BSc, MSc, andrea.clyne@thewaltoncentre.nhs.uk. Principal Investigator: Rhys Davies, MA, BM, BCh, PhD, FRCP
27. University College London Hospitals NHS Foundation Trust Recruiting, London, United Kingdom; Contact: Fiona Kinsella, 0203 108 2638, fiona.kinsella.19@ucl.ac.uk. Contact: Alexander Lowe, alexander.lowe.16@ucl.ac.uk. Principal Investigator: Edward Wild, MD, PhD
28. St. George’s University of London Recruiting, London, United Kingdom; Contact: Sally Goff, 0208 725 5375, sally.goff@nihr.ac.uk. Principal Investigator: Nayana Lahiri, MD
29. Oxford University Hospitals NHS Foundation Trust Recruiting, Oxford, United Kingdom; Contact: Zara Skitt, 01865 234309, zara.skitt@ouh.nhs.uk. Principal Investigator: Richard Armstrong, MA, PhD, BMBCh, MRCP
30. University Hospitals Plymouth NHS Trust Recruiting, Plymouth, United Kingdom; Contact: Abigail Patrick, 01752 439636, abigail.patrick1@nhs.net. Principal Investigator: Daniel Lashley, MBBS

University of Washington cohort: This was a prospective single-site study with standardized longitudinal collection of CSF, blood and phenotypic data. The study was performed in compliance with the IRB protocol at the University of Washington. All participants were required to provide informed consent before undertaking study procedures, and these informed consents were obtained by clinical site staff using approved processes.

*Criteria for subject group placement*. Healthy controls – Individuals who have no known family history of HD or have been tested for the *Huntingtin* gene glutamine codon expansion and are not at risk.

Premanifest HD – Individuals who do not have any clinical diagnostic motor features of HD, defined as UHDRS score <4 and who have a GAG expansion of ≥40 as well as a burden of pathology score (computed as (CAG − 35.5) × age) of between 130 and 560.

*Sample collection*. For CSF samples, lumbar punctures were carried out according to the guidelines stipulated in the IRB protocol at the University of Washington. In brief, patients were placed into the lateral decubitus position, and CSF was extracted from the subarachnoid space at the L3–L4 or L4–L5 interspace using a spinal needle. All samples were collected in a standardized manner with the method of extraction and storage controlled. CSF samples were screened for blood cell contamination of ≤10 erythrocytes and maintained at −80 °C. They were thawed on ice immediately before use. To further examine the degree of blood contamination or ascertain whether there is any evidence of blood–brain barrier breakdown in our samples, the CSF concentration of albumin, a molecule that constitutes around 50% of the total protein in the plasma, and which has also been shown in many studies to be present at relatively lower levels in the CSF, was quantified^166–169^. Previous studies using albumin labeled with radioactive iodine showed that the blood–brain barrier has a relatively low permeability for this species relative to other plasma proteins that undergo receptor-mediated transport and that it can reflect blood–brain barrier disruption under certain experimentally defined paradigms^117,170^. We found that, for all of the University of Washington samples, CSF albumin concentrations were below the average level found in a previous study of a large patient cohort (over 1,000 individuals whose serum:CSF albumin ratio was considered normal)^171^. Notably, there was also no correlation of albumin levels with patient CAP score (Supplementary Fig. 3j). These findings are consistent with a previous study that found no difference in the serum:CSF albumin ratio when comparing CSF samples from patients with HD and control (clinically normal) individuals^172^ and suggest that any disease-stage-associated changes in the analytes that we are assessing are unlikely to be driven by differences in the levels of blood contamination observed in these patient subject groups.

For serum samples, venous blood collection was carried out according to the guidelines stipulated in the IRB protocol at the University of Washington. In brief, blood was drawn and allowed to clot before centrifuging at 1,000–2,000*g* for 10 min at 4 °C. Serum supernatant was then aliquoted and stored at −80 °C.

#### Postmortem human tissue (clinical details for all samples can be found in Supplementary Table 2)

Fresh frozen tissue from the globus pallidus and caudate putamen of patients with HD and control individuals was a kind gift from Richard H. Myers at Boston University and the Los Angeles VA Human Brain and Spinal Fluid Resource Center. Fixed tissue sections were obtained through a collaboration with Richard Faull and Marika Esez at the New Zealand Human Brain Bank (University of Auckland). For all fixed samples, brain tissue was perfused with PBS containing 1% sodium nitrite, followed by a 15% formalin solution and post-fixation for 12–24 h. Samples were subsequently cryopreserved in 30% sucrose and snap frozen before being sectioned. The Huntington’s tissue assessed in this study was classified as having a neuropathological score of either Vonsattel grade 2 or grade 4. Grade 2 is classified as displaying mild to moderate striatal atrophy, with the medial outline of the head of the caudate nucleus (HCN) being only slightly convex but still protruding into the lateral ventricle. Significant loss of neurons and increases in astrocyte number are evident in the dorsal half of the caudate nucleus and adjacent putamen. Astrocytes also show altered size and increased GFAP immunoreactivity. Initial signs of neurodegeneration are evident in the pallidum, with the external segment displaying greater atrophy than the internal segment. Grade 4 is classified as displaying severe striatal atrophy, with the medial contours of the internal capsule and HCN now concave. There is also a 95% loss of striatal neurons^59,173^.

#### C1q function-blocking antibody

The C1q function-blocking antibody, termed ANX-M1 in some publications^30,34,115,174–177^, was generated by Annexon Biosciences (Annexon American Type Culture Collection (ATCC) accession no. PTA-120399) according to the protocol outlined in Hong et al.^30^ and batch tested in rodent hemolytic assays (for details, see Hong et al.^30^). ANX-M1 is the murine parent antibody of the humanized anti-C1q antibody ANX005, currently being employed in a phase 2 clinical study in patients with HD (ClinicalTrials.gov NCT04514367). Conjugation to FITC or alkaline phosphatase was carried out using Abcam’s alkaline phosphatase and FITC conjugation kits (Abcam, ab102850 and ab102884, respectively) according to the protocols outlined in the manufacturer’s instruction manuals.

#### Motor cortex injections

*P1/P2 mice*. Mice were anesthetized on ice, and a glass capillary attached to a stereotax was used to deliver 0.4 μl of pAAV2-hsyn-EGFP (Addgene viral prep no. 50465-AAV2, http://n2t.netaddgene, and 50465; RRID: Addgene_50465) into the motor cortex using a Nanoject III programmable nanoliter injector (VWR, 490019-810) and a micromanipulator MM33 (Pipette.com, 3-000-024-R). Mice were subsequently allowed to recover on a warm plate before being transferred back to their home cage. At P120, mice were killed, and microglial engulfment was assayed as described below. Using this AAV serotype and promoter, no microglia could be seen expressing the construct in the cortex, but both types of corticostriatal projection neurons, the pyramidal tract-type neurons, which are mainly found in layer V, and the intratelencephalical-type neurons, which are mainly found in layer III and the upper half of layer V, were targeted (Supplementary Fig. 1e,f). As expected, VGLUT1-immunolabeled synaptic terminals, denoting corticostriatal synapses, were found to co-localize with GFP signal in the dorsal striatum of P120-injected mice, but no overlap of GFP signal was observed with markers of the thalamostriatal synapse (Fig. 4a). This finding is similar to what was observed with the use of anterograde tracers in Lei et al.^67^.

*P300 mice*. Mice were anesthetized with isoflurane, and a drill was used to open a small hole in the skull. A glass pipette attached to a stereotax was subsequently used to deliver 0.4 μl of pAAV2-hsyn-EGFP (Addgene viral prep no. 50465-AAV2, http://n2t.netaddgene, and 50465; RRID: Addgene_50465). The skin was then closed with sutures, and the mice were allowed to recover in a warm chamber before being transferred back to the home cage. Fourteen days after injection, mice were killed, and microglial engulfment was assayed as described below.

#### IP injections

P120 mice were injected IP with either 40 mg kg^−1^ of the C1q function-blocking antibody or a control IgG (BioXCell, BE0083) every 48 h for 4 weeks. A similar injection paradigm was previously shown to enable uptake of iodine-125-labeled IgG antibodies into the brain^118^ and led to the C1q function-blocking antibody becoming detectable in the CSF^116^. The weight of the mice was monitored throughout the injection period and did not show any significant changes with either treatment (Extended Data Fig. 8e,f).

#### Ex vivo slice electrophysiology

*Acute brain slice preparation*. After 1 month of treatment with either the C1q function-blocking antibody or the control IgG, coronal sections (300-μm thickness) containing the striatum were prepared from the brains of 5-month-old zQ175 and WT littermates (as well as those of untreated mice). As described previously^178^, mice were anesthetized with isoflurane and transcardially perfused with ice-cold artificial cerebrospinal fluid (ACSF) solution (in mM): 125 NaCl, 2.5 KCl, 25 NaHCO_3_, 2 CaCl_2_, 1 MgCl_2_, 1.25 NaH_2_PO_4_ and 11 glucose (300–305 mOsm kg^−1^). After slicing the tissue in ice-cold ACSF on a Leica VT1200, slices were transferred for 10 min at 33 °C into a chamber filled with a choline-based recovery solution (in mM): 110 choline chloride, 25 NaHCO_3_, 2.5 KCl, 7 MgCl_2_, 0.5 CaCl_2_, 1.25 NaH_2_PO_4_, 25 glucose, 11.6 ascorbic acid and 3.1 pyruvic acid. Slices were then transferred to a second 33 °C chamber containing ACSF for 30 min. After this recovery period, the chamber was moved to room temperature for the duration of the experiment.

To isolate sEPSCs, recordings were performed at 28 °C in ACSF containing gabazine to block inhibitory activity. Electrodes with a resistance of 2.5–4.5 MΩ were pulled from borosilicate glass (Sutter Instrument) and filled with a cesium-based internal solution (in mM): 135 CsMeSO_3_, 10 HEPES, 1 EGTA, 3.3 QX-314 (Cl^−^ salt), 4 Mg-ATP, 0.3 Na-GTP, 8 Na_2_-phosphocreatine (pH 7.3 adjusted with CsOH; 295 mOsm kg^−1^). At a holding potential of −80 mV, spontaneous activity was recorded for 5–10 min per cell. All recordings were carried out blinded to genotype and treatment.

Due to the large cohort sizes for each experiment, mice were recorded in small groups over several days (with the timing of treatment onset staggered to accommodate this), with each set of recordings comprising mice treated with both the control IgG and the C1q function-blocking antibody.

*sEPSC analysis*. Data were collected with a Multiclamp 700B amplifier (Molecular Devices) and a National Instruments acquisition board using custom ScanImage MATLAB software (MathWorks). Voltage-clamp recordings were filtered at 2 kHz and digitized at 10 kHz. Only recording epochs with stable baseline potentials (<10% change) and access resistance (±10% baseline) were used for analysis. To identify the frequency and amplitude of sEPSCs, custom MATLAB scripts and analysis pipeline were adapted from Merel et al.^179^. This process was carried out blinded to genotype and treatment. To ensure equal weighting of each recorded cell, the data were bootstrapped so as to randomly sample 100 events per cell over the course of 1,000 iterations (the error bars on the cumulative probability distribution curves reflect the variance seen from this interrogation). To compare the cumulative distributions for the sEPSC inter-event intervals and amplitude, each iteration of the bootstrapped data was evaluated to determine if one distribution was greater than the other at the 75th percentile, with a potential detection threshold set at *P* = 0.0002 (one of 5,000 iterations). Statistical analyses were subsequently performed using GraphPad Prism software (GraphPad) and MATLAB.

#### IHC

IHC was performed as described in ref. ^95^ and ref. ^153^. In brief, brains were harvested from mice after transcardial perfusion with 15 ml of PBS and 15 ml of 4% paraformaldehyde (PFA). Tissue was then post-fixed in 4% PFA for 2 h before being washed in PBS and transferred to a 30% sucrose solution for 24–48 h. Subsequently, tissue samples were embedded in a 2:1 mixture of 30% sucrose-PBS:Tissue-Tek O.C.T. Compound (Electron Microscopy Sciences) and stored at −80 °C. Then, 14-μm or 25-μm cryosections were cut using a Leica cryostat and affixed to Leica Surgipath X-tra slides before being processed for IHC as follows. Slides were heated for 10 min at 35 °C, followed by three rinses in PBS. They were then blocked with either blocking solution (150 mM NaCl, 50 mM Tris Base pH 7.4, 5% BSA, 100 mM l-lysine, 0.04% sodium azide) and 0.3% Triton X-100 for staining with complement antibodies or 10% normal goat serum (NGS) and 0.3% Triton X-100 solution (Sigma-Aldrich) for 1 h, before being incubated with primary antibodies overnight (O/N) at 4 °C in 4:1 antibody buffer (150 mM NaCl, 50 mM Tris Base pH 7.4, 1% BSA, 100 mM l-lysine, 0.04% azide): blocking solution or 5% NGS (Sigma-Aldrich) and 0.3% Triton X-100 solution (Sigma-Aldrich). Slides were then washed for 3 × 15 min in PBS, followed by incubation with appropriate secondary antibodies in 4:1 antibody buffer:blocking solution or 5% NGS for 2 h at room temperature. Slides were then washed for 3 × 15 min in PBS and mounted with VECTASHIELD with DAPI (Vector Laboratories, H-1000). For engulfment analysis, microglial density quantifications and Iba-1:P2RY12 or Iba-1:TMEM119 co-localization analysis 40-μm free-floating sections were cut with a sliding microtome (Leica) and stained with relevant antibodies in 10% NGS and 0.3% Triton X-100 O/N at room temperature. All other steps are identical to those described above. After the final wash, the tissue was spread on the slide, and a coverslip was mounted on top using VECTASHIELD with DAPI (Vector Laboratories, H-1000).

For the engulfment analysis, images were acquired using an UltraVIEW VoX spinning disc confocal microscope and a ×60 plan apochromat oil objective (1.4 numerical aperture (NA)). Volocity image acquisition software was employed (PerkinElmer). For other confocal images, either an LSM 700 confocal microscope with ×25 or ×63 plan apochromat oil objectives (0.8 NA and 1.4 NA, respectively) and ZEN 2009 image acquisition software (Carl Zeiss) or an LSM 880 confocal microscope with ×10 dry or ×63 oil plan apochromat objectives (0.45 NA and 1.4 NA, respectively) and ZEN Black 2.3 image acquisition software (Carl Zeiss) or a LEICA SP8 white light confocal with a ×100 plan apochromat oil objective (1.4 NA) was used (for each of the different types of analysis performed and all of the comparisons between genotypes or treatment groups, the same microscope and imaging setup was used to collect images from every sample). For structured illumination microscopy, a Zeiss ELYRA PS1 microscope with a ×100 plan apochromat objective (1.57 NA) and ZEN Black 2012 image acquisition software was used. Finally, for array tomography analysis, an AxioImager Z1 microscope with a ×60 plan-neofluar objective (1.4 NA) and AxioVision software (Carl Zeiss) was used.

The antibody dilutions used were as follows: Homer1 (Synaptic Systems (160003)) 1:200, VGLUT2 (Millipore Sigma (AB2251)), 1:1,000 IHC, 1:10,000 WB, VGLUT1 (Millipore Sigma (AB5905)) 1:1,000 IHC, 1:10,000 WB, Iba1 (Wako (019-19741)), 1:400, Iba1 (Wako (ncnp24)) 1:200, CD68 (Serotec clone FA-11 (MCA1957)), 1:200, CD68 (Dako (M087629-2)) 1:200, CD11b (Serotec clone 5C6 (MCA711G)), 1:200, clone 5C6, β-actin (Sigma-Aldrich (A2228)), 1:1,000 WB, C1q (Abcam (ab182451)) (validated in C1qA KO mice, https://www.abcam.com/c1q-antibody-48-ab182451.html#description_images_1), 1:500, C1q (Dako (A0136)) 1:500, C1q (JL-1) (Abcam (ab71940)) 1:500, C3d (Dako (A0063)), 1:500, C3c (Dako (F0201)) 1:1,000, iC3b (Quidel (A209)) 1:500, PSD-95 (Millipore (MAB1596)) 1:500; S100β (Dako (Z0311)) 1:500, TMEM119 (Abcam (ab209064)) 1:200, P2RY12 (Anaspec (AS-55043A)) 1:500 anti-fluorescein-POD (Roche (11 426 346 910)) 1:2,000, anti-digioxigenin (Roche (11207733910)) 1:2,000, Alexa Fluor-conjugated secondary antibodies (Life Technologies, A-11073, goat anti-guinea pig IgG (H+L) Highly Cross-Adsorbed Secondary Antibody, Alexa Fluor 488, A-11006, goat anti-rat IgG (H+L) Cross-Adsorbed Secondary Antibody, Alexa Fluor 488, A-11012, goat anti-rabbit IgG (H+L) Cross-Adsorbed Secondary Antibody, Alexa Fluor 594, A-21245, goat anti-rabbit IgG (H+L) Highly Cross-Adsorbed Secondary Antibody, Alexa Fluor 647) 1:250, goat anti-rabbit IgG H&L alkaline phosphatase (Abcam (ab97048)) 1:5,000, goat anti-rabbit HRP (Promega (W4011)) 1:5,000, goat anti-mouse HRP (Promega, W4021) 1:5,000 and Peroxidase-AffiniPure donkey anti-guinea pig IgG (H+L) (Jackson ImmunoResearch, 706-035-148) 1:5,000. Further details can be found in the Key Resources Table.

#### Microglia density quantification

To quantify microglial cell density, 40-μm sections from zQ175 heterozygote mice and WT littermates, collected at the specified ages, were stained with Iba1, and three fields of view in the dorsal striatum were imaged per animal (*n* = 4–5 per genotype). Microglia were then manually counted in each ×25 magnification field (65,571 μm^2^) using ImageJ software (NIH), and these values were averaged to generate a per-field number for each animal. All analysis was performed blinded to genotype.

#### Assessing changes in microglia morphology and lysosomal protein levels

Twenty-five-μm sections from zQ175 heterozygote mice and WT littermates, collected at the specified ages, were immunolabeled with CD68 and Iba1 and imaged with a ×25 pan apochromat oil objective (0.8 NA) using a Zeiss LSM700 confocal microscope with 0.4-μm *z*-steps. Three fields of view were captured in relevant brain regions, and maximum intensity projections (MIPs) were generated with Zeiss software. The phagocytic state of each microglia was categorized based on morphology and CD68 levels using an adapted version of the protocol in Schafer et al.^95^, with 0 being the least phagocytic and 5 the most. In brief, process morphology was scored as 0 (thin long processes with multiple branches), 1 (thicker processes but with similar branching), 2 (thick retracted processes with few branches) or 3 (no clear processes). CD68 was analyzed and scored as 0 (no/scarce expression), 1 (punctate expression) or 2 (aggregated expression or punctate expression all over the cell). For each cell analyzed, morphology and CD68 scores were summed, and a final score for microglia phagocytic state (0–5) was assigned. Between 30 and 50 cells were assessed per animal, and the number of microglia with a given score was represented as a percentage of the total population. All analysis was performed blinded.

Sholl analysis was performed by surface-rendering Iba1 staining using Imaris 9.3 software (Oxford Instruments) with the use of splitting algorithms to identify individual cells. The Filaments tool in Imaris was then used to trace individual cells and subsequently identify and quantify both branch points and terminal projections at different distances from the soma. Over 100 cells were analyzed for each genotype, and all analysis was performed blinded.

#### Assessing changes in CD11b levels

Sections from 3-month-old zQ175 heterozygote mice and WT littermates were immunolabeled with CD11b and Iba1 antibodies and imaged with a ×25 pan apochromat oil objective (0.8 NA) with 0.4-μm *z*-steps. Three fields of view were captured in relevant brain regions, and MIPs were generated with Zeiss software. ImageJ software was then used to assess the mean gray value of CD11b staining within individual fields of view that were subsequently averaged to generate a value for each animal. Between 30 and 50 cells were analyzed from each animal, and all analysis was performed blinded to genotype.

#### Engulfment analysis

zQ175 heterozygous homer GFP mice and their WT homer GFP littermates or zQ175 heterozygous mice and WT littermates that had received an injection of pAAV2-hsyn-EGFP were killed at P120 and P314 and transcardially perfused with 15 ml of ice-cold PBS, followed by 15 ml of 4% PFA. The brains of these mice were then dissected out and post-fixed for 2 h in 4% PFA before being washed and placed in a 30% sucrose solution in PBS for storage at 4 °C for 24–48 h (as described above). Brains were subsequently sectioned on a sliding microtome (Leica), and 40-μm sections were stained with Iba1 or S100β (as described above). S100β is thought to be a marker of all known astrocyte populations in the striatum and has identified similar numbers of cells to those seen in ALDH1eGFP mice^103^. Images from the dorsal striatum ipsilateral to the injection site were then acquired on an UltraVIEW VoX spinning disc confocal microscope using a 0.2-μm *z*-step. For each animal, 10–15 cells were imaged. Images were subsequently processed and analyzed as described previously using ImageJ (NIH) and Imaris (Bitplane) software^95,100,153^. The data generated were used to calculate percent engulfment (volume engulfed GFP / volume of the cell) and input density (total volume GFP inputs / volume of field of view). All experiments were performed blinded to genotype.

One potential caveat of this method of engulfment analysis is that it cannot exclude the contribution of autofluorescent granules, which have been reported to be present in the lysosomes of microglia, to the signal being analyzed^180–182^. However, when we quantified autofluorescence (the signal generated by exciting at a wavelength that will not activate the GFP fluorophore being expressed or any of the other fluorophores used for staining), we observed that less than 10% of the CD68-stained lysosome area was actually autofluorescent and, more importantly, that there was no difference in the autofluorescence area per field or the area of autofluorescence co-localized with CD68 when comparing WT and zQ175 heterozygote mice (Supplementary Fig. 1c,d).

#### Synapse quantification and complement co-localization with synaptic markers

*For confocal images*. A modified version of the protocol outlined in Hong et al.^30^ was employed. In brief, 14-μm sections were collected from mice with different genotypes at the ages specified in the text, figure legends and supplementary tables and from mice after treatment with different agents. They were stained with appropriate antibodies to synaptic or complement proteins and imaged with a Zeiss LSM 700, a Zeiss LSM 880 or a Leica SP8 white light confocal microscope. Three fields of view were captured at ×63 magnification in relevant brain regions (101.6 μm^2^), and, for each field, a 3-μm *z*-stack comprising 1-μm *z*-steps was imaged. *z*-planes were captured at a depth at which complement and synaptic staining was uniform across the field of view. ImageJ, ilastik and CellProfiler software were subsequently used to quantify the number of co-localized presynaptic and postsynaptic puncta or the number of co-localized complement and presynaptic puncta in each *z*-plane (nine images total for each animal) using a method that incorporates thresholding the images into a binary state and identifying overlapping puncta that fulfil specific dimension criteria. These were then averaged to generate a per-field number for each animal. All analysis was performed blinded to genotype and treatment.

*For structured illumination images*. A modified version of the protocol outlined in Hong et al.^183^ was employed. In brief, 14-μm sections from zQ175 heterozygote mice, zQ175 CR3 KO mice and WT littermates were stained with appropriate synaptic markers or antibodies to complement proteins and imaged with a Zeiss Elyra PS1 microscope. Three fields of view were captured at ×100 magnification in relevant brain regions, and, for each field, a 3-μm *z*-stack comprising 0.01-μm *z*-steps was imaged. Zeiss software and proprietary algorithms were subsequently used to generate structured illumination microscopy (SIM)-processed image files (images can appear slightly saturated because the demodulation algorithm used to reconstruct the image lacks a means of converting the raw detected signal into photon counts). To quantify different types of synapses or the number of synapses co-localized with different complement proteins, image files were opened in Imaris, and spot channels were generated for each set of immunoreactive puncta using dimensions determined empirically from averaged measurements. A MATLAB plugin (MathWorks) was then used to display and quantify only those spots within a threshold distance from each other (measured from the center of each spot). Depending on the analysis, the number of co-localized spots was normalized to the total number of one of the spot populations. For the complement co-localization experiments, an additional control was performed by rotating the channel containing the complement staining 90° relative to the VGLUT1 images to enable assessment of the amount of co-localization that would be expected to occur by chance. Statistical tests were then used to compare the fold change relative to WT in the % of synaptic puncta co-localizing with complement proteins in the rotated versus the non-rotated images. All analysis was performed blinded to genotype.

*For array tomography*. Array tomography was performed as previously described with minor modifications^1,95,157,184–187^. In brief, 300-μm vibratome sections of dorsal striatum from 7-month-old WT and zQ175 heterozygote mice were fixed in 4% PFA for 1.5 h at room temperature and embedded in LR White Resin. This tissue was then further sectioned to generate ribbons of between 20 and 30 serial 70-nm-thick sections, which were mounted on subbed glass coverslips and immunostained with VGLUT1 and Homer1 antibodies. The same field of view was subsequently imaged on serial sections using a Zeiss AxioImager Z1 microscope with a ×63 objective. A three-dimensional projection of this field was then obtained by aligning these images with ImageJ and AutoAligner (Bitplane) software. Finally, projections were analyzed using Imaris to quantify co-localized puncta using the spots function, as outlined above for analysis of SIM images. Analysis was performed blinded to genotype.

#### Detecting C1q function-blocking antibody in the neuropil

P120 zQ175 heterozygous mice were injected IP with 40 mg kg^−1^ of FITC-conjugated or unconjugated M1. Twenty-four hours after injection, mice were killed, and their brains were harvested after transcardial perfusion with PBS and 4% PFA. Tissue was then post-fixed in 4% PFA for 2 h, washed and transferred to a 30% sucrose solution. Then, 14-μm cryosections were subsequently prepared from tissue embedded in a 2:1 mixture of 20% sucrose:OCT and blocked with a 5% BSA and 0.2% Triton X-100 solution for 1 h before being incubated with anti-fluorescein-POD (Roche (11 426 346 910 1:2,000)) O/N. Signal was detected and amplified using the TSA Staining Kit (PerkinElmer, NEL0701001KT) according to the manufacturer’s instructions

#### IHC using postmortem human tissue sections

Postmortem human tissue was fixed according to the procedures outlined in Waldvogel et al.^188^. In brief, the brain was extracted, and the basal and internal carotid arteries were identified. The tissue was subsequently perfused by attaching winged infusion needles to these arteries and flowing through a solution of PBS containing 1% sodium nitrite, followed by 3 L of a fixative containing 15% formalin. After perfusion, the brain was post-fixed for 12–24 h in the same fixative before being dissected into blocks for sectioning. Tissue was stained by washing 25-μm free-floating postmortem human tissue sections for 3 × 5 min in PBS and then permeabilizing them in a 0.2% Triton X-100 PBS solution for 1 h at room temperature. Sections were subsequently blocked in a 10% BSA, 0.2% Triton X-100 PBS solution for 1 h at room temperature before applying appropriate primary antibodies in a 5% BSA, 0.2% Triton X-100 PBS solution O/N at 4 °C. Sections were then washed for 3 × 5 min in PBS before adding appropriate secondary antibody in 5% BSA PBS for 1 h at room temperature. After washing for a further 3 × 5 min in PBS, sections were incubated in a 0.5% Sudan Black solution dissolved in 70% ethanol to reduce autofluorescence from lipofuscin vesicles. Sections were then washed a further 7× in PBS to remove excess Sudan Black before being spread onto slides and left to dry. Coverslips were mounted in 90% glycerol PBS containing Hoechst (diluted 1:1,000).

#### Construction of a C3 expression construct

A mammalian expression plasmid containing the cDNA for mouse C3 was constructed according to the protocol outlined below. C3 cDNA obtained from Open Biosystems/Horizon (MMM1013-202768722; clone ID 5134713) was amplified using a two-step strategy. In the first step, the restriction site Sma I and a Kozac sequence were added to the 5′ end before a sequence encompassing everything up to the Xba I restriction site (2,287 bp) was amplified. In the second step, a restriction site BamH I and a stop codon were added at the end of the gene (2,705 bp). Both sequences were subsequently inserted into the pUltra EGFP plasmid (Addgene, 24129) by subcloning with Xba I and BamH I. The primer sequences employed were as follows: C3 beg Smal 1F (cccggggccaccatgggaccagcttcagggtcccagc); C3 beg Xbal 2R (tctagagataatatcttcttctgg); C3 end Xbal 1F (tctagaagccacttcccacagagc); C3 end BamH I 2R (ggatcctcagttgggacaaccataaacc).

#### Testing the specificity of the anti-C3d antibody by carrying out immunocytochemistry on HEK cells expressing a mouse C3 construct

HEK293 cells (ATCC, CRL-1573) grown on coverslips were transfected using Lipofectamine 2000 (Invitrogen, 11668-019) and 1–2 μg of either pULTRA EGFP or pULTRA EGFP T2A C3 Ms (see above) (a culture in which no DNA was employed in the transfection was included as a control). Twenty-four hours after transfection, cells were fixed using 4% PFA for 10 min at room temperature. After washing for 3 × 5 min with PBS, cells were subsequently blocked for 30 min in a 10% goat serum PBS solution. Cells were then incubated O/N at 4 °C with anti-C3d diluted 1:500 in a 10% goat serum, 0.2% Triton PBS solution. After washing for 3 × 5 min with PBS, goat anti-rabbit Alexa Fluor 594-conjugated secondary antibody diluted 1:1,000 in PBS was added to the cells for 30 min at room temperature. Cells were then washed again, and the coverslips were mounted using VECTASHIELD with DAPI (Vector Laboratories, H-1000). Staining was subsequently visualized on a Zeiss AxioImager Z.1 (Extended Data Fig. 1n). Note that, although the complement C3d antibody employed here was raised against purified C3d from human plasma, it is a polyclonal entity and, as such, contains species that bind to multiple different epitopes within the structure of the protein. Given that the mouse and human protein sequences for complement component C3 share a 77% identity (NCBI BLAST), the cross-reactivity of this antibody, which we demonstrate in Extended Data Fig. 1n, should not be unexpected.

#### Fluorescence in situ hybridization

*C1qa/C1QA* and *C3* antisense RNA probes were made and labeled with digoxigenin as described in Liddelow et al.^189^, and the NSE antisense RNA probe was labeled with fluorescein. In brief, plasmids for human *C3* (Open Biosystems reference MMM1013-202769931), human *C1QA* (Open Biosystems reference MMM1013-202702004) and mouse *C1qa* (Open Biosystems reference MMM1013-202704027) were digested with Sal I before RNA was transcribed from the T7 promoter. Alkaline hydrolysis was then performed at 60 °C for 30 min to fragment the target RNA before labeling it with digoxigenin and fluorescein according to kit instructions (Roche).

Labeled RNA probes were subsequently applied to 14-μm cryosections of mouse and human tissue according to the procedure outlined below. In brief, sections were initially prepared as described above for IHC analysis, before being dried at 65 °C for 30 min. Sections were then incubated in 100% methanol at −20 °C for 20 min before being washed for 3 × 5 min in PBS. Sections were subsequently treated with a 1 μg ml^−1^ proteinase K solution (1 μg ml^−1^ in 50 mM Tris pH 7.5 and 5 mM EDTA) for 10 min before being washed again for 3 × 5 min in PBS. Tissue sections were then re-fixed in 4% PFA/PBS for 5 min before being washed for 3 × 5 min in PBS. Sections were subsequently acetylated by being incubated in a solution containing 100 mM triethanolamine, 1.8 mM HCl and 0.5% acetic anhydride before being washed for 3 × 5 min in PBS and permeabilized in a 1% Triton/PBS solution for 30 min at room temperature. Endogenous peroxidase activity was then blocked by incubating in a 0.3% hydrogen peroxide solution for 30 min before washing for 2 × 5 min in PBS and then finally hybridizing with relevant probes O/N at 64 °C. Bound probes were detected with anti-digoxigenin and anti-fluorescein antibodies (Roche), and staining was amplified using a TSA Staining Kit (PerkinElmer, NEL0701001KT) according to the manufacturer’s instructions. Immunostaining for Iba1 and S100β was subsequently performed as described for IHC. The mouse *C1qa* probe was validated on tissue from *C1qa* KO mice (a kind gift from Marina Botto, Imperial College London) (data not shown), and sense probes for human *C1Q* and *C3* were found to generate no detectable signal when employed on sequential tissue sections (data not shown).

#### Single-molecule fluorescence in situ hybridization

Fourteen-μm cryosections of mouse brain tissue were generated according to the procedures outlined above for IHC, with the exception that, once extracted, mouse brains were post-fixed for 24 h in 4% PFA before being washed and placed in a 30% sucrose solution in PBS for storage at 4 °C for 24–48 h (as described above). Subsequent processing and sectioning steps were the same. Once generated, sections were prepared for in situ hybridization using the procedures outlined by Advanced Cell Diagnostics (ACD) branched DNA technology (RNAscope) for employing fixed frozen tissue in their multiplexed fluorescent amplification assay (ACD user manual nos. 320535 and 320293). In brief, sections were washed 3× in PBS before undergoing target retrieval by being placed into boiling 1× retrieval solution (ACD, 322000) for 5 min. After washing in distilled water, tissue sections were dehydrated by being placed into 100% ethanol. After this, tissue was subsequently permeabilized by treatment with protease III (ACD, 322340) for 30 min at 40 °C. After tissue preparation was complete, hybridization using probes to *C3* (ACD, 417841), *Acta2* (ACD, 319531-C2), *C1qa* (ACD, 441221-C2), *P2ry12* (ACD, 317601) and *FoxJ1* (ACD, 317091) was carried out along with subsequent steps to amplify and detect signal. All procedures were performed according to the guidelines stipulated in the multiplexed fluorescent amplification assay manual (ACD, 320293). Imaging was carried out using a Zeiss LSM 880 confocal microscope and ZEN Black 2.3 image acquisition software (Carl Zeiss). Tissue quality was assessed as good by having robust and uniform signal after hybridization with the RNAscope triplex positive control probes (ACD, 320881) and minimal signal with the triplex negative control (ACD, 320871, a probe to DapB, a bacterial transcript). Quantification of *C3* and *C1q* IF puncta was carried out using QuPath software version 0.4.2.

#### Quantitative RT–PCR

Microdissected mouse and human brain tissue was flash frozen and homogenized in TRIzol using a TissueLyser II (Qiagen). Phenol/chloroform extraction was then used together with the RNeasy Mini Kit (Qiagen) to isolate and purify RNA. RNA quantity was subsequently measured using a NanoDrop (Thermo Fisher Scientific), and cDNA was synthesized from all samples by employing SuperScript II reverse transcriptase (Thermo Fisher Scientific). After cDNA synthesis, qPCR reactions were assembled for the gene of interest and a housekeeping gene (*Gapdh*) (see Key Resources Table for the sequences of all primers used) using SYBR Green (Qiagen). Reactions were run on a Rotor-Gene qPCR machine (Qiagen), and expression levels were compared using the ΔΔCt method with normalization to *Gapdh* levels.

#### Immunoblotting

Microdissected mouse and human brain tissue was flash frozen on dry ice. Frozen tissue was then transferred into a HEPES-based lysis buffer (25 mM HEPES, 0.1 M NaCl, 1% Triton X-100) containing cOmplete Protease Inhibitor Cocktail (Millipore Sigma), and a 5-mm stainless steel bead was subsequently used to homogenize the tissue using the TissueLyser II system (Qiagen) for 2 × 5 min at 20 Hz. Lysates were then spun for 10 min at 15,000*g* in a benchtop centrifuge to pellet insoluble material before the supernatant was removed to a new tube, and protein concentration was assayed using the Pierce BCA Protein Assay Kit (Thermo Fisher Scientific, 23225) according to the manufacturer’s instructions. Protein samples (20 μg) were then mixed with 2× Laemmli buffer (Bio-Rad) before being loaded onto 10% tris-glycine gels and separated for 1 h at 150 V by SDS-PAGE. Separated proteins were transferred onto nitrocellulose membranes (GE Healthcare Amersham), which were subsequently blocked in 5% milk PBS for 1 h at room temperature before being probed with relevant antibodies O/N at 4 °C in 5% milk PBS. Membranes were then washed for 3 × 5 min in PBS before being incubated with relevant secondary antibodies diluted in 5% milk PBS for 1 h at room temperature. After washing again for 3 × 5 min in PBS, bands were visualized with luminol-based enhanced chemiluminescence HRP substrate (SuperSignal West Dura, Thermo Fisher Scientific) and the ImageQuant LAS 4000 system (GE Healthcare) avoiding saturation of any pixels. To strip membranes and re-probe with a different antibody, ReBlot Plus Strong Antibody Stripping Solution (EMD Millipore) was employed according to the manufacturer’s instructions before re-blocking in 5% milk and staining. For quantification, the intensity of bands generated by staining with synaptic antibodies was normalized to those generated by a β-actin antibody (employed as a loading control) in the same lane. ImageJ software was used to carry out densitometry analysis of all bands, which was performed blinded to genotype. Full-length images of all representative immunoblot images in the manuscript can be found in Source Data Extended Data Fig. 2.

#### ELISA assays

For tissue samples, microdissected human brain tissue was flash frozen and lysed to extract proteins using a TissueLyser II system (Qiagen), and total protein concentration was determined using the Pierce BCA Protein Assay Kit, as described above for immunoblotting. In the case of human CSF and plasma samples, these were acquired, prepared and stored as described above. For subsequent interrogation of complement protein levels, all samples were employed in the C3 (Abcam, ab108822 and ab108823), iC3b, C1q or CR3 sandwich ELISAs according to the procedures outlined below. Notably, for every ELISA, the same 96-well plate configuration was employed with two columns of standards and two dilutions of each sample, with technical replicates employed for each dilution. In addition, two common reference samples and four blank wells were included on each plate for calibration, and a duplicate of every plate was tested. For both the CSF and plasma samples on each plate, the gender balance and average age for each clinical group was kept equal and consistent with the average age of that clinical group as a whole. All assays were run on automated (Bravo liquid handler, Agilent) or semi-automated (VIAFLO, INTEGRA) platforms using novel pipelines, and analysis was performed blinded to sample type.

*C3 ELISA*. Five μg of protein extracted from frozen human tissue, CSF (diluted 1:400 and 1:800 in sample diluent) or plasma (diluted 1:166.6 and 1:333.2 in sample diluent) samples were employed in C3 sandwich and competitive ELISAs (Abcam, 108823 (for CSF and tissue samples) and 108822 (for plasma), respectively). The same lot of both ELISA kits was employed for analysis of all samples, and the assay was performed according to the manufacturer’s instructions. In addition, before employing this assay, tests were carried out to confirm that minimal signal was generated with C3-depleted serum (data not shown).

*iC3b ELISA*. Sixteen μg of protein extracted from frozen human tissue, CSF (diluted 1:5 and 1:10 in PBS) or plasma (diluted 1:400 and 1:800 in PBS) samples was employed in an iC3b sandwich ELISA with a monoclonal iC3b antibody (Quidel, A209; diluted 1:500) used for capture and a polyclonal C3 antibody (Dako, F0201; diluted 1:1,000) used for detection.

Before employing this assay, tests were carried out to confirm that minimal signal was generated with C3-depleted serum; that it could detect increased levels of iC3b after in vitro complement activation (either through incubation of serum at 4 °C for 7 d or zymosan treatment) (Extended Data Fig. 1k,l); and that it was selective for iC3b over native C3 and other C3 cleavage fragments (Extended Data Fig. 1m).

*C1Q ELISA*. CSF (diluted 1:25 and 1:50 in PBS) or plasma (diluted 1:125 or 1:250 in PBS) samples were employed in a C1q sandwich ELISA with a polyclonal rabbit antibody (Abcam, ab71940; diluted 1:50) used for capture and a monoclonal C1q antibody (Annexon M1) conjugated with alkaline phosphatase used for detection (diluted 1:2,000). Before employing this assay, tests were carried out to confirm that minimal signal was generated with C1q-depleted serum.

*CR3 ELISA*. Sixteen μg of protein extracted from frozen human tissue samples was employed in a human CR3 (CR3/ITGAM) ELISA (LSBio, LS-F11850). The same lot of this kit was employed for the analysis of all samples, and the assay was performed according to the manufacturer’s instructions.

*Hemoglobin ELISA*. Sixteen μg of protein extracted from frozen human tissue samples was employed in a hemoglobin sandwich ELISA (Abcam, ab157707). The same lot of this kit was employed for the analysis of all samples, and the assay was performed according to the manufacturer’s instructions.

*Albumin ELISA*. CSF (diluted 1:2,000 and 1:4,000 in sample diluent) samples were employed in an albumin sandwich ELISA (Abcam, ab108788). The same lot of this kit was employed for the analysis of all samples, and the assay was performed according to the manufacturer’s instructions.

All non-commercial ELISA assays were found to produce consistent results, with an intra-assay coefficient of between 7% and 10% for both the iC3b and C1q ELISA assays based on internal quality control samples.

*Age adjustment calculation*. To control for the effects of normal aging on CSF concentrations of complement proteins in patients with HD, the CSF concentrations of C3 and iC3b in control (clinically normal) individuals were regressed on the basis of patient age. The regression coefficient generated from this, termed *b*, was then employed in the below calculation where Y_hdi is the observation of the protein level in HDGECs; age_hdi is the corresponding age; and mean(age) is the mean age in the control (clinically normal) population.

$${\rm{Adjusted}}({\rm{Y\_hdi}})={\rm{Y\_hdi}}-{\rm{b}}({\rm{age\_hdi}}-{\rm{mean}}({\rm{age}}))$$

*CAP score calculation*. The CAP calculation was initially devised by Penny et al.^190^ to estimate the progression of HD pathology as a function of both CAG repeat length and the time of exposure to the effects of the expansion and was driven by the observation that the age of clinical onset in HD is strongly influenced by the length of the CAG trinucleotide expansion within the HTT gene. The authors found that an index of this form was a good predictor of striatal pathology in the brains of patients with HD at the time of autopsy. Subsequent studies have shown correlations between CAP score and levels of fluid biomarkers, motor and cognitive performance and differences in neuropsychiatric assessments and structural and molecular imaging markers^131,136,138–140,142,191–196^. It is important to note, however, that the CAP score formula itself does not incorporate any direct metric or measure of pathology.

When employed in this manuscript, the CAP score is defined as follows:

$${\rm{CAP}}=100{\rm{\times }}{\rm{Age}}{\rm{\times }}[({\rm{CAG}}-{\rm{L}})/{\rm{S}}]$$

where CAG is the patient’s CAG repeat length; Age is the patient’s current age at the time of observation; and L and S are constants of 30 and 627, respectively. S is a normalizing constant chosen so that the CAP score is approximately 100 at the patient’s expected age of onset as estimated by Langbehn et al.^165^. L is a scaling constant that anchors CAG length approximately at the lower end of the distribution relevant to HD pathology. Intuitively, L might be thought of as the lower limit of the CAG lengths for which some pathological effect might be expected.

*In vitro complement activation protocols*. Serum samples from control (clinically normal) individuals or serum samples in which C3 and C4 were inactivated were either thawed and maintained at 4 °C for 7 d or treated with 10 mg ml^−1^ zymosan for 30 min at 37 °C. They were then immediately employed in the iC3b ELISA detailed above.

#### Operant touchscreen visual discrimination and cognitive flexibility assays

*Apparatus*. Two-choice visual discrimination and cognitive flexibility assays were performed in Bussey–Saksida operant touchscreen chambers— sealed enclosures that have a trapezoidal wall shape structure with a touch-sensitive computer screen at the front and a feeder tray at the rear through which a liquid reward can be dispensed (Lafayette Instrument Company, 80614). The chambers were set up with a 1 × 2 mask to constrain access to two portions of the screen and operated using ABET II touch software housed on a WhiskerServer Controller. All chambers were configured using the standard touch screen environment file provided by Lafayette Instrument Company, and the hardware was tested before running programs using the 12-TouchMouseTestLines protocol in ABET II and the display test pattern feature in WhiskerServer to ensure that all screens and input/output responses were functioning as proscribed. All chambers were housed in a sound-attenuating cupboard.

*Food restriction procedures*. To motivate task engagement, all animals underwent a diet restriction paradigm before testing in which the quantity of food pellets that they were allowed access to was restricted. The mice were weighed on a daily basis, and a sufficient quantity of pellets was provided such that their mass was maintained at 80–85% of their original free-feeding weight. Food restriction was begun 1 week before training and continued throughout the testing period.

Training and shaping tasks. After the mice had reached target body weights, they were placed in the operant touchscreen chambers, and a habituation program was run to familiarize them with collecting liquid reward from the feeder tray. This program involved a tone being played and a light simultaneously being turned on in the feeder tray alongside the presentation of a liquid reward (a solution containing 14% sugar). Reward was presented on a variable schedule in which mice received a total of 60 reinforcers per 60-min session. All mice underwent two sessions of this feeder habituation on consecutive days.

After completion of feeder training, mice were familiarized with the touchscreen interface by running a ‘must touch’ program in which physical interaction with a random object presented at different locations on the screen was required to initiate the dispensing of a reward. Random geometric shapes consisting of similar dimensions and pixel intensities were presented at variable locations across the base of the screen and physical interaction with the displayed image, resulted in it being removed from the screen and a simultaneous dispensing of reward with the same mechanics of tone presentation and illumination of the feeder that were operated during feeder habituation. Images were presented on a variable schedule in which mice were offered the opportunity to complete 60 trials in a 60-min period. All mice underwent three sessions of this training on consecutive days, and, by the end, all had reached the criteria of completing at least 50 trials in a 60-min period. The 1 × 2 mask was not employed during this training.

Acquisition phase of visual discrimination learning. In this phase of the task, mice were presented with two different visual stimuli of similar size and pixel density that were randomly displayed in the two locations on the screen where access was not restricted by the 1 × 2 mask. Physical interaction with one of these two stimuli resulted in reward presentation through the same mechanisms outlined in the training section. If, however, the mice interacted with the other image presented, a different tone was played, and a light positioned to illuminate the chamber was switched on for 3 s. In addition, no reward was dispensed. A trial session was complete when the mice had performed 60 of these trials or 60 min had elapsed. The inter-trial interval was 2 seconds, and images were never presented in the same location more than three times consecutively. For each trial session, the total number of trials performed, the % of those trials in which the rewarded visual presentation was chosen and the latency to collect the reward were recorded. Mice were not allowed to move on to the reversal phase until they had interacted with the rewarded stimuli in 70% of the trials carried out within a session on at least three occasions (with one trial session being carried out per day).

*Reversal phase*. In this phase of the task, interaction with the previously rewarded stimulus resulted in presentation of the same aversive cues and non-dispense of the liquid reward that interacting with the previously unrewarded image yielded and vice versa. In all other aspects, the procedures followed during reversal were identical to those of the acquisition phase. Mice underwent 15 d of reversal testing, with a separate session of 60 trials carried out each day regardless of performance in the task.

Progressive ratio. After completion of testing in the reversal phase, mice were transitioned to a progressive ratio task in which one image was displayed in a central location on the screen. At the start of this assessment, a single physical interaction with this image resulted in reward being dispensed in the same manner as that described above; however, after completion of the first trial, the image remained on the screen until a second physical interaction had taken place, and only then was reward dispensed. This pattern was continued up to the fifth trial where five physical interactions were required to elicit a reward. For all subsequent trials after this point, five physical interactions were required to complete the trial and get the reward. The mice were assessed on how many trials of this nature they were able to complete in a 60-min period. All mice underwent progressive ratio testing in this format for 3 d. After this point, the whole procedure was repeated but this time with a food pellet present in the chamber. Both the total number of trials completed and the mass of food pellet consumed were recorded.

Image bias assessment. To discern whether mice show a pre-existing preference for either of the two images used in the visual discrimination learning and reversal tests, a separate cohort of 4-month-old C57BL/6J mice (JAX stock no. 000664) was randomly assigned to two groups, one in which the standard stimuli reward associations previously used for testing were employed and one in which these were reversed. The acquisition and reversal phases of the task were then carried out as described above. This testing revealed that there is a small but significant preference for one of the two visual stimuli that exists before any reinforcement learning takes place and that is maintained throughout testing (Extended Data Fig. 7l,m). This might explain the slightly lower than chance performance of all mice on day 1 of testing, but, as all of the genotypes assayed received equal numbers of trials in which this stimulus was associated with reward presentation, it is likely not responsible for the genotype-dependent differences observed.

Visual acuity assessment using the optomotor test. Visual acuity was assessed using the optomotor task outlined in Pursky et al.^197^. In brief, each mouse was placed unrestrained on a platform surrounded by four computer monitors that were set to display a rotating cylinder covered with a vertical sine wave grating (set at 100% contrast) that was projected in three-dimensional space. Mice reflexively tracked the grating with head and neck movements that could be documented by an observer. The spatial frequency of the grating was subsequently clamped at the viewing position by repeatedly re-centering the cylinder on the head of the mouse, and visual acuity was assessed by gradually increasing the frequency until a reflexive optomotor response could no longer be detected. This was designated as the spatial frequency threshold of visual acuity, and it was subsequently obtained for each direction of rotation. Previous studies showed that visual acuity increases markedly between the second and fourth postnatal weeks, but it remains stable at a spatial frequency of 0.4 cycles per degree after that point in C57BL/6J mice that have not undergone any procedures and that were maintained in similar conditions (JAX stock no. 000664) (ref. ^197^).

#### Open field assessment

Ambulatory activity and aspects of exploratory behavior were assessed using the Kinder Scientific Smart Frame Open Field System and Motor Monitor II software during the dark phase of the light cycle. This is a time period where mice are more active and during which previous studies showed a greater ability to discriminate between the performance of different treatments or genotypes^198,199^. In brief, infrared beam breaks caused by movements of the mice in the chamber were recorded over a 30-min window and then binned into 5-min intervals to provide metrics for the total time the mice spent in the center of the chamber, the total distance they traveled around the periphery and, finally, the number of rearing events that took place.

#### Statistical analysis and reproducibility

All experiments were repeated a minimum of three times, and, where images from representative exeriments are shown in the absence of accompanying graphs showing quantification of a particular metric, the precise number is listed in the figure legends. For all statistical analyses, GraphPad Prism 7 and Stata SE software were used. For data generated from mouse models, consultation on appropriate statistical tests was carried out with Kush Kapor, associate director of biostatistics at the Harvard Clinical and Translational Science Center. For data generated from CSF and plasma analysis, consultations on power calculations and appropriate statistical tests were carried out with Jen Ware, director of experimental design, and John Warner, director of biostatistics, at the CHDI Foundation.

To determine appropriate sample sizes for the CSF and plasma analysis, a pilot study was performed using a small number of CSF and serum samples (sourced from the University of Washington). Based on the effect sizes observed, a power analysis was performed using G*Power version 3.1.9.2, which determined the number of individuals required to detect the same effect with 80% power at an alpha level of 2.5% (corrected for two primary comparisons using the Bonferroni method).

For all data analysis, tests for normality (D’Agostino–Pearson normality test) were performed where appropriate. When comparing two groups, an unpaired two-tailed *t*-test, multiple unpaired two-tailed *t*-tests or the non-parametric Kolmogorov–Smirnov test were performed as appropriate. When comparing more than two groups, one-way ANOVA (fixed effects, omnibus) was used, followed by post hoc testing with Tukey’s multiple comparison test with a 95% confidence interval. For all comparisons involving more than two groups and data generated from human CSF and plasma, the non-parametric Kruskal–Wallis test was employed, followed by post hoc testing with Dunn’s multiple comparison test and a family-wise significance and confidence level of 0.05. The two-tailed non-parametric Spearman correlation test with a confidence interval of 95% was employed to test the significance of relationships between two variables within the CSF and plasma datasets. For the CSF, plasma and tissue extract analysis, potentially confounding variables, such as age, gender and blood contamination, were examined and, where appropriate, were included as covariates or adjusted for. For the age adjustment calculation, linear regression was used to compare the relationship between patient age and analyte concentration in control (clinically normal) individuals. All *P* values are indicated in both the figure panels and the figure legends.

#### Availability of biological materials and reagents

All unique biological materials, with the exception of the C1q function-blocking antibody, plasma, CSF and postmortem samples, whose distribution is limited by material transfer agreements, are available from the authors upon reasonable request or from standard commercial sources. Further information about this and requests should be directed to the lead contact, Beth Stevens (beth.stevens@childrens.harvard.edu).

### Reporting summary

Further information on research design is available in the Nature Portfolio Reporting Summary linked to this article.

## Online content

Any methods, additional references, Nature Portfolio reporting summaries, source data, extended data, supplementary information, acknowledgements, peer review information; details of author contributions and competing interests; and statements of data and code availability are available at 10.1038/s41591-023-02566-3.

### Supplementary information

Supplementary Information Supplementary Figs. 1–3, Supplementary Tables 1–4 and Key Resources Table.

Reporting Summary

Supplementary Data 1 Source data for graphs and statistical analysis associated with Supplementary Fig. 1.

Supplementary Data 2 Source data for graphs and statistical analysis associated with Supplementary Fig. 2.

Supplementary Data 3 Source data for graphs and statistical analysis associated with Supplementary Fig. 3.

### Source data

Source Data Fig. 1 Source data for graphs and statistical analysis associated with Fig. 1.

Source Data Fig. 2 Source data for graphs and statistical analysis associated with Fig. 2.

Source Data Fig. 3 Source data for graphs and statistical analysis associated with Fig. 3.

Source Data Fig. 4 Source data for graphs and statistical analysis associated with Fig. 4.

Source Data Fig. 5 Source data for graphs and statistical analysis associated with Fig. 5.

Source Data Fig. 6 Source data for graphs and statistical analysis associated with Fig. 6.

Source Data Extended Data Fig./Table 1 Source data for graphs and statistical analysis associated with Extended Data Fig. 1.

Source Data Extended Data Fig./Table 2 Source data for graphs and statistical analysis associated with Extended Data Fig. 2 and full-length, unprocessed blots associated with the immunoblot data presented in Extended Data Fig. 2c,g,l.

Source Data Extended Data Fig./Table 2 Source data for graphs and statistical analysis associated with Extended Data Fig. 2 and full-length, unprocessed blots associated with the immunoblot data presented in Extended Data Fig. 2c,g,l.

Source Data Extended Data Fig./Table 3 Source data for graphs and statistical analysis associated with Extended Data Fig. 3.

Source Data Extended Data Fig./Table 4 Source data for graphs and statistical analysis associated with Extended Data Fig. 4.

Source Data Extended Data Fig./Table 5 Source data for graphs and statistical analysis associated with Extended Data Fig. 5.

Source Data Extended Data Fig./Table 6 Source data for graphs and statistical analysis associated with Extended Data Fig. 6.

Source Data Extended Data Fig./Table 7 Source data for graphs and statistical analysis associated with Extended Data Fig. 7.

Source Data Extended Data Fig./Table 8 Source data for graphs and statistical analysis associated with Extended Data Fig. 8.

Source Data Extended Data Fig./Table 9 Source data for graphs and statistical analysis associated with Extended Data Fig. 9.

Source Data Extended Data Fig./Table 10 Source data for graphs and statistical analysis associated with Extended Data Fig. 10.

## Data Availability

All data supporting the findings of this study can be found within the article and its Extended Data and Source data files. Extended Data Fig. 1 has associated raw data for the immunoblots that can be located in Source Data Figs. 11 and 12. The biological repository identifiers for the CSF and plasma samples from the HDClarity cohort are restricted from distribution as a result of guidelines stipulated in the material transfer agreement. This was mandated by the foundation providing this material to ensure that IRB guidelines regarding the protection of participantsʼ personal information and identity are not disclosed. Further information about this, as well as the procedures and application forms required to gain access to this information, can be found at https://hdclarity.net/ and https://enroll-hd.org/. The timeframe from request to provision of data can take 1–2 months depending on the information technology and security infrastructure at your site. Source data are provided with this paper.

## Code availability

Details of the custom MATLAB scripts and analysis pipelines used to identify the frequency, amplitude and membrane properties of sEPSCs in the slice electrophysiology experiments are documented in ref. ^179^. All code related to FIJI, Ilastik and Cell Profiler pipelines, as well as the two-choice visual discrimination, reversal and progressive ratio tasks, will be made freely available upon reasonable request.
