# Author Correction: Microglia and complement mediate early corticostriatal synapse loss and cognitive dysfunction in Huntington’s disease

**DOI:** 10.1038/s41591-023-02663-3

**Published:** 2023-11-02

**Authors:** Daniel K. Wilton, Kevin Mastro, Molly D. Heller, Frederick W. Gergits, Carly Rose Willing, Jaclyn B. Fahey, Arnaud Frouin, Anthony Daggett, Xiaofeng Gu, Yejin A. Kim, Richard L. M. Faull, Suman Jayadev, Ted Yednock, X. William Yang, Beth Stevens

**Affiliations:** 1 F. M. Kirby Neurobiology Center, Department of Neurology, Boston Children’s Hospital, Harvard Medical School, Boston, MA US; 2 Center for Neurobehavioral Genetics, Jane and Terry Semel Institute for Neuroscience and Human Behavior, Department of Psychiatry and Biobehavioral Sciences, David Geffen School of Medicine at University of California, Los Angeles, CA USA; 3 Department of Anatomy with Radiology, Faculty of Medical and Health Sciences, University of Auckland, Auckland, New Zealand; 4 Department of Neurology, University of Washington, Seattle, WA USA; 5 Division of Medical Genetics, Department of Medicine, University of Washington, Seattle, WA USA; 6 Annexon Biosciences, South San Francisco, CA USA; 7 Stanley Center, Broad Institute, Cambridge, MA USA; 8 Howard Hughes Medical Institute, Boston Children’s Hospital, Harvard Medical School, Boston, MA USA

**Keywords:** Microglia, Huntington's disease

Correction to: *Nature Medicine* 10.1038/s41591-023-02566-3, published online 9 October 2023.

In the version of this article initially published, the first name of Jaclyn B. Fahey appeared as Jacyln and has now been corrected in the HTML and PDF versions of the article.

