# Supplementary material for: Microglia and complement mediate early corticostriatal synapse loss and cognitive dysfunction in Huntington’s disease
Source: Nat Med. 2023 Oct 9;29(11):2866–84. doi: 10.1038/s41591-023-02566-3 (PMC10667107; doi:10.1038/s41591-023-02566-3)
Supplement: Supplementary file 1 — Supplementary Figs. 1–3, Supplementary Tables 1–4 and Key Resources Table. [file 41591_2023_2566_MOESM1_ESM.pdf]

nature medicine

Article https://doi.org/10.1038/s41591-023-02566-3

Microglia and complement mediate early corticostriatal synapse loss and cognitive dysfunction in Huntington's disease

In the format provided by the authors and unedited

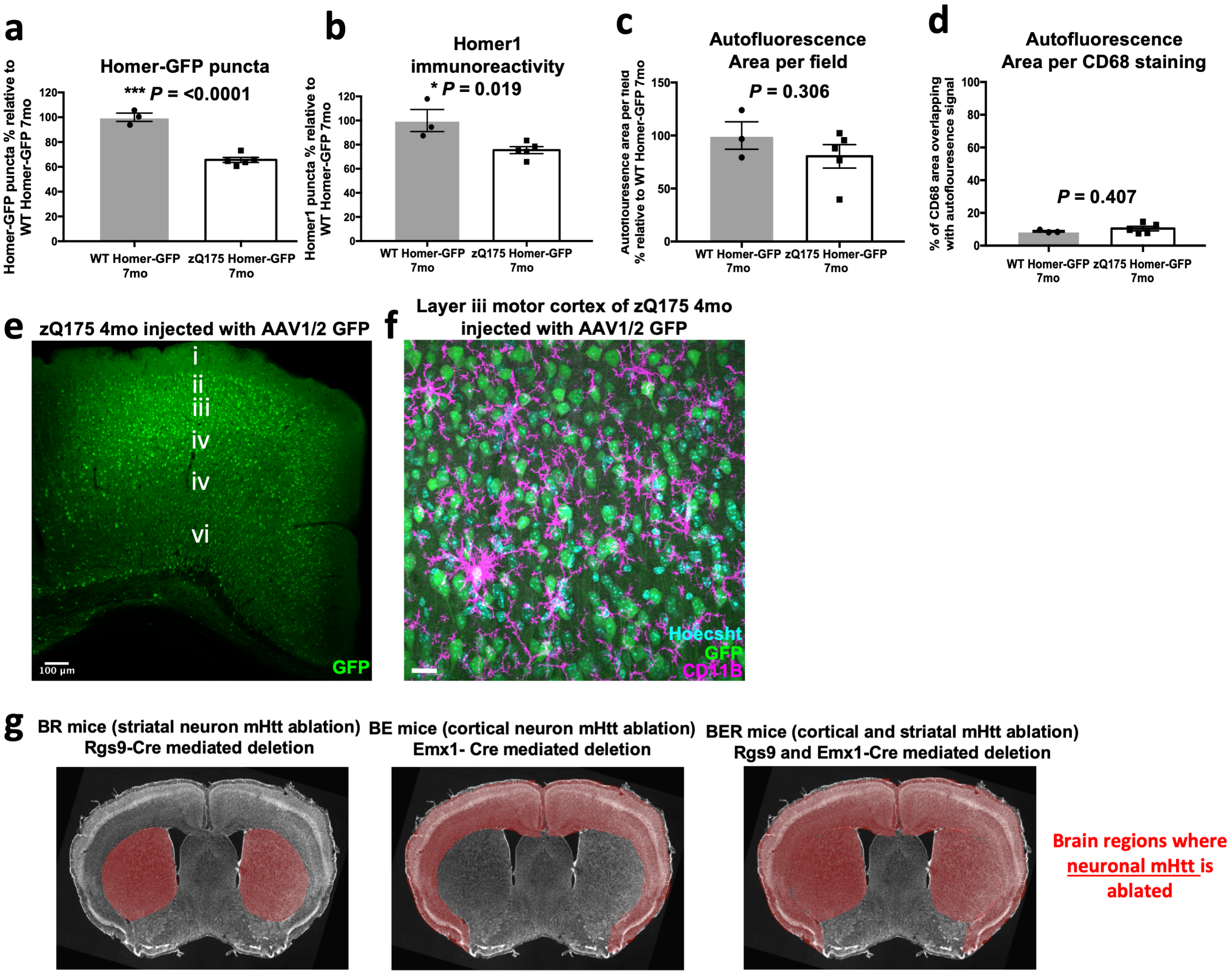
a
Homer-GFP puncta
*** P = <0.0001
Homer-GFP puncta % relative to WT Homer-GFP 7mo
0
20
40
60
80
100
120
WT Homer-GFP 7mo
zQ175 Homer-GFP 7mo
b
Homer1 immunoreactivity
* P = 0.019
Homer1 puncta % relative to WT Homer-GFP 7mo
0
20
40
60
80
100
120
WT Homer-GFP 7mo
zQ175 Homer-GFP 7mo
c
Autofluorescence Area per field
P = 0.306
Autoflourescence area per field % relative to WT Homer-GFP 7mo
0
50
100
150
WT Homer-GFP 7mo
zQ175 Homer-GFP 7mo
d
Autofluorescence Area per CD68 staining
P = 0.407
% of CD68 area overlapping with autoflouresence signal
0
20
40
60
80
100
WT Homer-GFP 7mo
zQ175 Homer-GFP 7mo
e
zQ175 4mo injected with AAV1/2 GFP
i
ii
iii
iv
iv
vi
100 µm
GFP
f
Layer iii motor cortex of zQ175 4mo injected with AAV1/2 GFP
Hoecsht
GFP
CD11B
g
BR mice (striatal neuron mHtt ablation) Rgs9-Cre mediated deletion
BE mice (cortical neuron mHtt ablation) Emx1- Cre mediated deletion
BER mice (cortical and striatal mHtt ablation) Rgs9 and Emx1-Cre mediated deletion
Brain regions where neuronal mHtt is ablated

Supplemental data figure 1: (a,b) Bar charts showing the percentage of Homer-GFP puncta **(a)** and the percentage of Homer1 immunoreactive puncta **(b)** in 7 mo zQ175 Homer-GFP mice relative to that seen in 7 mo WT Homer-GFP littermates, n=3 WT Homer-GFP and 5 zQ175 Homer-GFP mice (2F and 1M for WT Homer-GFP and 3F and 2M for zQ175 Homer-GFP). Unpaired t-test, for Homer-GFP puncta p=<0.0001; for Homer1 immunoreactivity p=0.019. **(c)** Bar chart showing the relative % area per field occupied by auto-fluorescent signal in 7 mo zQ175 Homer-GFP mice relative to that seen in 7 mo WT Homer-GFP littermates, n=3 WT Homer-GFP and 5 zQ175 Homer-GFP mice (2F and 1M for WT Homer-GFP and 3F and 2M for zQ175 Homer-GFP). Unpaired t-test p=0.306. **(d)** Bar chart showing the % of CD68 area overlapping with autofluorescent signal in 7 mo zQ175 Homer-GFP mice and WT Homer-GFP littermates, n=3WT Homer-GFP and 5 zQ175 Homer-GFP mice (2F and 1M for WT Homer-GFP and 3F and 2M for zQ175 Homer-GFP). Unpaired t-test p=0.407. **(e)** Representative 10x confocal image of the motor cortex of a 4 mo zQ175 mouse injected with pAAV2-hsyn-EGFP showing the distribution of transduced cells in different cortical layers. Scale bar =100 μm. **(f)** Representative 60x confocal image of layer iii of the motor cortex of a 4 mo zQ175 mice injected with pAAV2-hsyn-EGFP showing transduced cells and microglia denoted by CD11B staining. Scale bar = 10 μm. **(e)** Coronal section of a mouse brain showing the brain regions (highlighted in red) in which *mHtt* has been genetically removed from selected neuronal populations in BR (RGS-9 cre to excise *mHtt* in striatal neurons) BE (Emx-1 cre to excise *mHtt* in cortical neurons) and BER (Rgs-9 and Emx-1 cre to excise *mHtt* in both striatal and cortical neurons) mice. For bar charts, bars depict the mean. All error bars represent SEM. Stars depict level of significance with *=p<0.05, **p=<0.01 and ***p<0.0001.

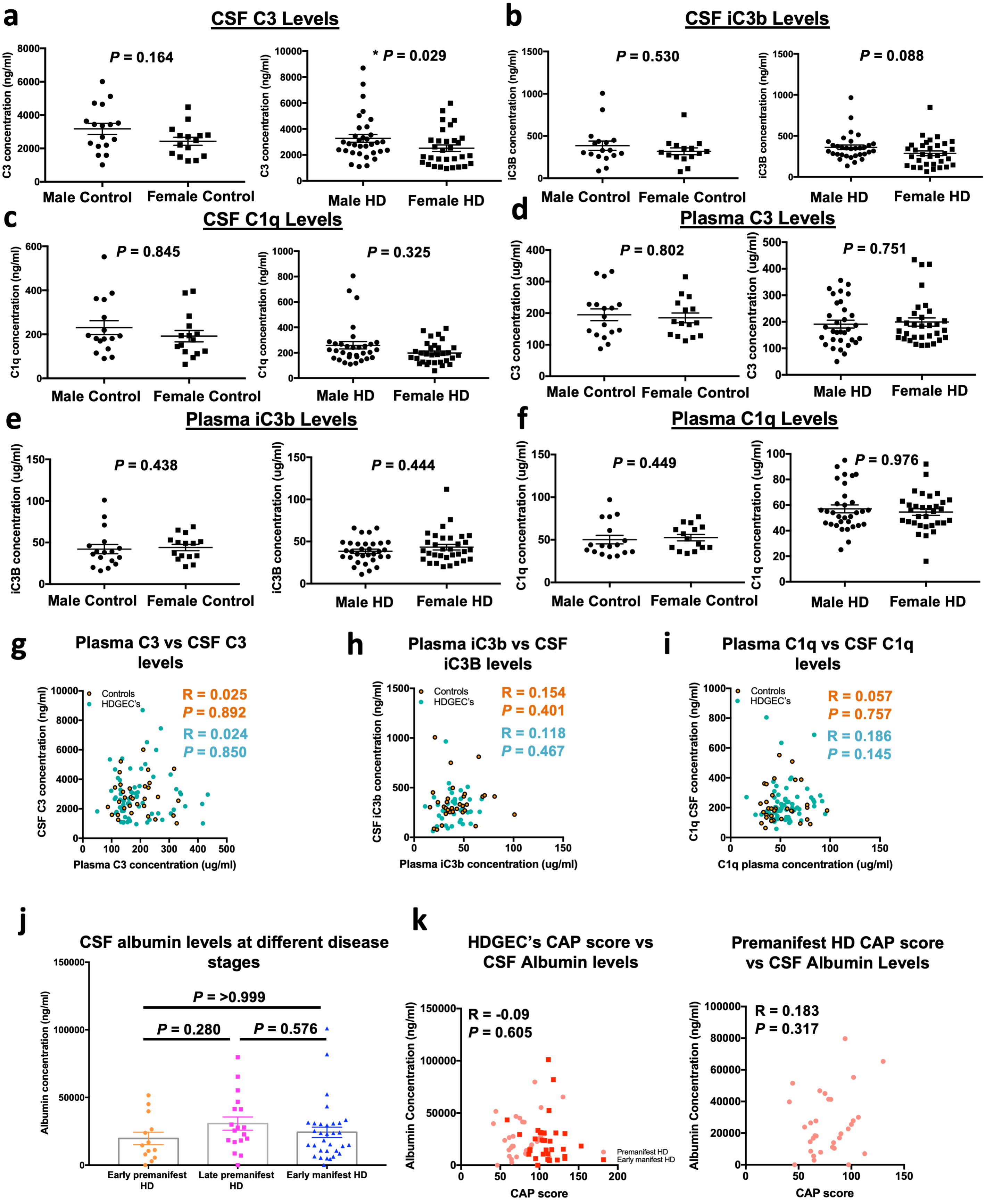
a
CSF C3 Levels
P = 0.164
* P = 0.029
C3 concentration (ng/ml)
Male Control
Female Control
Male HD
Female HD
b
CSF iC3b Levels
P = 0.530
P = 0.088
iC3B concentration (ng/ml)
c
CSF C1q Levels
P = 0.845
P = 0.325
C1q concentration (ng/ml)
d
Plasma C3 Levels
P = 0.802
P = 0.751
C3 concentration (ug/ml)
e
Plasma iC3b Levels
P = 0.438
P = 0.444
iC3B concentration (ug/ml)
f
Plasma C1q Levels
P = 0.449
P = 0.976
C1q concentration (ug/ml)
g
Plasma C3 vs CSF C3 levels
Controls
HDGEC's
R = 0.025
P = 0.892
R = 0.024
P = 0.850
CSF C3 concentration (ng/ml)
Plasma C3 concentration (ug/ml)
h
Plasma iC3b vs CSF iC3B levels
R = 0.154
P = 0.401
R = 0.118
P = 0.467
CSF iC3b concentration (ng/ml)
Plasma iC3b concentration (ug/ml)
i
Plasma C1q vs CSF C1q levels
R = 0.057
P = 0.757
R = 0.186
P = 0.145
C1q CSF concentration (ng/ml)
C1q plasma concentration (ug/ml)
j
CSF albumin levels at different disease stages
P = >0.999
P = 0.280
P = 0.576
Albumin concentration (ng/ml)
Early premanifest HD
Late premanifest HD
Early manifest HD
k
HDGEC's CAP score vs CSF Albumin levels
R = -0.09
P = 0.605
Albumin Concentration (ng/ml)
Premanifest HD
Early manifest HD
CAP score
Premanifest HD CAP score vs CSF Albumin Levels
R = 0.183
P = 0.317

Supplementary data figure 2: (a) Graphs showing the C3 concentration in CSF samples from males and females in the control (clinically normal) group and in CSF samples from males and females with HD (both premanifest and manifest (HDGEC's)). Each dot represents a sample from a separate individual and the line denotes the mean; for the controls n=17 males and 15 females, in the HD samples n=32 males and 31 females. Kolmogorov-Smirnov test p=0.164 for the controls and p=0.029 for the HDGEC's. **(b)** Graphs showing the iC3b concentration in CSF samples from males and females in the control (clinically normal) group and in CSF samples from males and females with HD (both premanifest and manifest (HDGEC's)). Each dot represents a sample from a separate individual and the line denotes the mean; for the controls n=17 males and 15 females, in the HD samples n=32 males and 31 females. Kolmogorov-Smirnov test p=0.530 for the controls and p=0.088 for the HDGEC's. **(c)** Graphs showing the C1q concentration in CSF samples from males and females in the control (clinically normal) group and in CSF samples from males and females with HD (both premanifest and manifest (HDGEC's)). Each dot represents a sample from a separate individual and the line denotes the mean; for the controls n=17 males and 15 females, in the HD samples n=32 males and 31 females. Kolmogorov-Smirnov test p=0.845 for the controls and p=0.325 for the HDGEC's. **(d)** Graphs showing the C3 concentration in plasma samples from males and females in the control (clinically normal) group and in CSF samples from males and females with HD (both premanifest and manifest (HDGEC's)). Each dot represents a sample from a separate individual and the line denotes the mean; for the controls n=17 males and 15 females, in the HD samples n=33 males and 31 females. Kolmogorov-Smirnov test p=0.802 for the controls and p=0.751 for the HDGEC's. **(e)** Graphs showing the iC3b concentration in plasma samples from males and females in the control (clinically normal) group and in CSF samples from males and females with HD (both premanifest and manifest (HDGEC's)). Each dot represents a sample from a separate individual and the line denotes the mean; for the controls n=17 males and 15 females, in the HD samples n=33 males and 31 females. Kolmogorov-Smirnov test p=0.438 for the controls and p=0.444 for the HDGEC's. **(f)** Graphs showing the C1q concentration in plasma samples from males and females in the control (clinically normal) group and in CSF samples from males and females with HD (both premanifest and manifest (HDGEC's)). Each dot represents a sample from a separate individual and the line denotes the mean; for the controls n=17 males and 15 females, in the HD samples n=33 males and 31 females. Kolmogorov-Smirnov test p=0.449 for the controls and p=0.976 for the HDGEC's. **(g)** Graph showing the association between plasma C3 concentration and CSF C3 concentration in samples from Huntington's Disease gene expansion carriers (HDGECs) as well as those from control (clinically normal) individuals recruited into the HDClarity study. Each dot represents a sample from a separate

individual, n=63 HDGEC's and n=32 controls. Spearman r for HDGEC's p=0.850; for controls p=0.892. **(h)** Graph showing the association between plasma iC3b concentration and CSF iC3b concentration in samples from Huntington's Disease gene expansion carriers (HDGECs) as well as those from control (clinically normal) individuals recruited into the HDClarity study. Each dot represents a sample from a separate individual, n=63 HDGEC's and n=32 controls. Spearman r for HDGEC's p=0.467; for controls p=0.401. **(i)** Graph showing the association between Plasma C1q concentration and CSF C1q concentration in samples from Huntington's Disease gene expansion carriers (HDGECs) as well as those from control (clinically normal) individuals recruited into the HDClarity study. Each dot represents a sample from a separate individual, n=63 HDGEC's and n=32 controls. Spearman r for HDGEC's p=0.145; for controls p=0.757. **(j)** Bar chart showing the concentration of albumin in CSF samples from early premanifest HD patients (see methods for inclusion criteria), late premanifest HD patients (see methods for inclusion criteria) and early manifest HD patients (see methods for inclusion criteria) recruited into the HDClarity study. Each dot represents a sample from a separate individual and the bar denotes the mean for each group n=13 early premanifest HD, n=18 late premanifest HD and n=32 early manifest HD. Kurkasl-Wallis test (non-parametric ANOVA) p=0.215 with early premanifest versus late premanifest HD p=0.280, late premanifest versus early manifest HD p=0.576 and early premanifest versus early manifest HD p=>0.999 via Dunn's multiple comparison test. **(k)** Graphs showing the association between CAP score and CSF albumin concentration for all samples from Huntington's Disease gene expansion carriers (HDGECs) as well as those just from premanifest HD patients recruited into the HDClarity study. Each dot represents a sample from a separate individual n=63 HDGEC's and n=31 premanifest HD. Spearman r for HDGEC's p=0.605; for premanifest HD p=0.317. For bar charts and dot plots, bars and lines depict the mean. All error bars represent SEM. Stars depict level of significance with *=p<0.05, **p=<0.01 and ***p<0.0001.

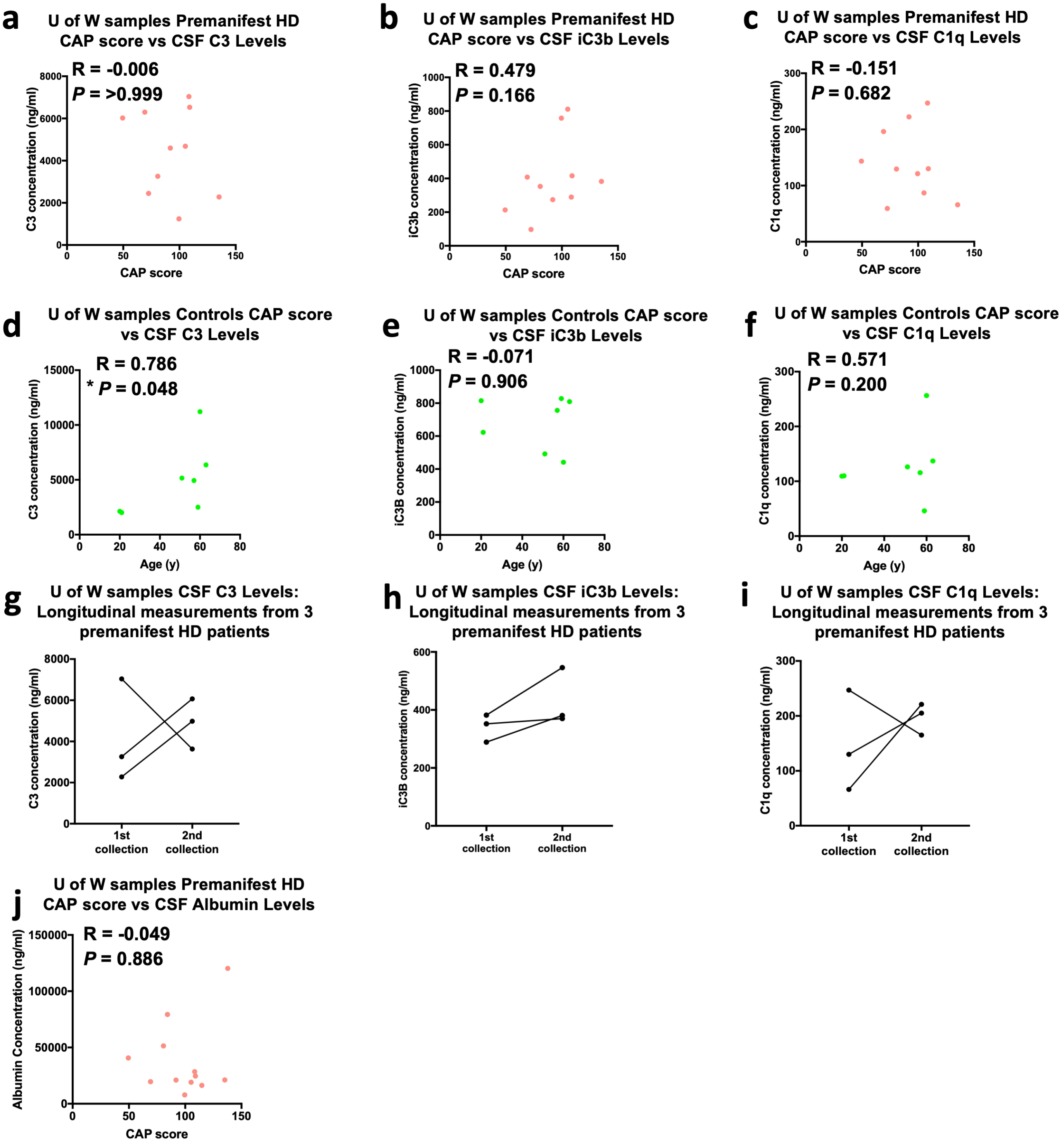
a
U of W samples Premanifest HD
CAP score vs CSF C3 Levels
R = -0.006
P = >0.999
C3 concentration (ng/ml)
CAP score
b
U of W samples Premanifest HD
CAP score vs CSF iC3b Levels
R = 0.479
P = 0.166
iC3b concentration (ng/ml)
CAP score
c
U of W samples Premanifest HD
CAP score vs CSF C1q Levels
R = -0.151
P = 0.682
C1q concentration (ng/ml)
CAP score
d
U of W samples Controls CAP score
vs CSF C3 Levels
R = 0.786
* P = 0.048
C3 concentration (ng/ml)
Age (y)
e
U of W samples Controls CAP score
vs CSF iC3b Levels
R = -0.071
P = 0.906
iC3B concentration (ng/ml)
Age (y)
f
U of W samples Controls CAP score
vs CSF C1q Levels
R = 0.571
P = 0.200
C1q concentration (ng/ml)
Age (y)
g
U of W samples CSF C3 Levels:
Longitudinal measurements from 3
premanifest HD patients
C3 concentration (ng/ml)
1st collection
2nd collection
h
U of W samples CSF iC3b Levels:
Longitudinal measurements from 3
premanifest HD patients
iC3B concentration (ng/ml)
1st collection
2nd collection
i
U of W samples CSF C1q Levels:
Longitudinal measurements from 3
premanifest HD patients
C1q concentration (ng/ml)
1st collection
2nd collection
j
U of W samples Premanifest HD
CAP score vs CSF Albumin Levels
R = -0.049
P = 0.886
Albumin Concentration (ng/ml)
CAP score

Supplementary data figure 3: (a) Graph showing the association between CAP score and CSF C3 concentration in samples from premanifest HD patients recruited into the University of Washington study. Each dot represents a sample from a separate individual n=10 Spearman r p=>0.999. **(b)** Graph showing the association between CAP score and CSF iC3b concentration in samples from premanifest HD patients recruited into the University of Washington study. Each dot represents a sample from a separate individual n=10 Spearman r p=0.166. **(c)** Graph showing the association between CAP score and CSF C1q concentration in samples from premanifest HD patients recruited into the University of Washington study. Each dot represents a sample from a separate individual n=10 Spearman r p=0.682. **(d)** Graph showing the association between patient age and CSF C3 concentration in samples from control (clinically normal) individuals recruited into the University of Washington study. Each dot represents a sample from a separate individual n=7 Spearman r p=0.048. **(e)** Graph showing the association between patient age and CSF iC3b concentration in samples from control (clinically normal) individuals recruited into the University of Washington study. Each dot represents a sample from a separate individual n=7 Spearman r p=0.906. **(f)** Graph showing the association between patient age and CSF C1q concentration in samples from control (clinically normal) individuals recruited into the University of Washington study. Each dot represents a sample from a separate individual n=7 Spearman r p=0.200. **(g)** Graph showing CSF C3 concentrations in samples from 3 premanifest HD patients from the University of Washington cohort that underwent a 2nd lumbar puncture approximately 1.5yr after the 1st sampling (see methods) n=3. **(h)** Graph showing CSF iC3b concentrations in samples from 3 premanifest HD patients from the University of Washington cohort that underwent a 2nd lumbar puncture approximately 1.5yr after the 1st sampling (see methods) n=3. **(i)** Graph showing CSF C1q concentrations in samples from 3 premanifest HD patients from the University of Washington cohort that underwent a 2nd lumbar puncture approximately 1.5yr after the 1st sampling (see methods) n=3. **(j)** Graph showing the association between CAP score and CSF albumin concentration for all premanifest samples from the University of Washington. Each dot represents a sample from a separate individual n=12 (this includes the 2nd collection samples referenced in panels g,h and i and excludes one sample employed in a, b and c which had a CAP score of 73, and was unfortunately no longer available). Spearman r p=0.886. Stars depict level of significance with *=p<0.05, **p=<0.01 and ***p<0.0001

Supplemental Table 1

Study cohort	Sample ID	Sex	Age	HD Category	CAG high	CAG low
HDClarity	EMHD1	male	54.4	early HD	43	17
HDClarity	EMHD2	female	50.5	early HD	43	17
HDClarity	EMHD3	female	60.3	early HD	39	15
HDClarity	EMHD4	male	69	early HD	41	15
HDClarity	EMHD5	male	39	early HD	42	18
HDClarity	EMHD6	female	52.3	early HD	43	10
HDClarity	EMHD7	male	55.8	early HD	43	17
HDClarity	EMHD8	male	63.9	early HD	43	23
HDClarity	EMHD9	male	42.3	early HD	44	25
HDClarity	EMHD10	female	58.5	early HD	44	23
HDClarity	EMHD11	female	58.6	early HD	42	19
HDClarity	EMHD12	male	50.9	early HD	44	17
HDClarity	EMHD13	male	67.2	early HD	41	21
HDClarity	EMHD14	male	52.8	early HD	42	18
HDClarity	EMHD15	female	45.8	early HD	44	10
HDClarity	EMHD16	male	42.3	early HD	43	17
HDClarity	EMHD17	male	45.9	early HD	43	17
HDClarity	EMHD18	female	55.4	early HD	45	22
HDClarity	EMHD19	female	63.2	early HD	41	17
HDClarity	EMHD20	male	64.1	early HD	45	18
HDClarity	EMHD21	male	33.5	early HD	50	18
HDClarity	EMHD22	female	47.5	early HD	44	18
HDClarity	EMHD23	female	55.2	early HD	40	17
HDClarity	EMHD24	male	57.8	early HD	42	18
HDClarity	EMHD25	male	63.5	early HD	41	17
HDClarity	EMHD26	female	70	early HD	41	17
HDClarity	EMHD27	female	40.9	early HD	45	17
HDClarity	EMHD28	male	47.3	early HD	43	18
HDClarity	EMHD29	male	52.6	early HD	37	17
HDClarity	EMHD30	female	58.9	early HD	41	18
HDClarity	EMHD31	female	60	early HD	41	20
HDClarity	EMHD32	male	69.3	early HD	42	15
HDClarity	EPMHD1	female	26.4	early pre-manifest HD	41	15
HDClarity	EPMHD2	male	51.5	early pre-manifest HD	40	17
HDClarity	EPMHD3	male	33.4	early pre-manifest HD	42	20
HDClarity	EPMHD4	female	47	early pre-manifest HD	40	17

HDClarity	EPMHD5	female	52.6	early pre-manifest HD	40	18
HDClarity	EPMHD6	female	35	early pre-manifest HD	41	18
HDClarity	EPMHD7	male	25.3	early pre-manifest HD	41	17
HDClarity	EPMHD8	female	29.1	early pre-manifest HD	42	17
HDClarity	EPMHD9	male	25.9	early pre-manifest HD	40	21
HDClarity	EPMHD10	female	31	early pre-manifest HD	43	17
HDClarity	EPMHD11	female	33.8	early pre-manifest HD	42	22
HDClarity	EPMHD12	female	34.8	early pre-manifest HD	41	16
HDClarity	EPMHD13	male	37	early pre-manifest HD	42	19
HDClarity	LPMHD1	male	41	late pre-manifest HD	42	17
HDClarity	LPMHD2	female	33.7	late pre-manifest HD	45	15
HDClarity	LPMHD3	male	22.7	late pre-manifest HD	47	17
HDClarity	LPMHD4	female	64.2	late pre-manifest HD	41	17
HDClarity	LPMHD5	female	49.1	late pre-manifest HD	43	20
HDClarity	LPMHD6	male	57	late pre-manifest HD	41	17
HDClarity	LPMHD7	male	31.4	late pre-manifest HD	44	21
HDClarity	LPMHD8	female	24.5	late pre-manifest HD	47	22
HDClarity	LPMHD9	male	54.4	late pre-manifest HD	45	18
HDClarity	LPMHD10	female	41.8	late pre-manifest HD	46	20
HDClarity	LPMHD11	female	47.1	late pre-manifest HD	42	21
HDClarity	LPMHD12	male	53.6	late pre-manifest HD	41	17
HDClarity	LPMHD13	male	27.4	late pre-manifest HD	45	18
HDClarity	LPMHD14	female	43.6	late pre-manifest HD	44	17
HDClarity	LPMHD15	male	45.7	late pre-manifest HD	44	19
HDClarity	LPMHD16	female	50.6	late pre-manifest HD	41	21
HDClarity	LPMHD17	female	29.9	late pre-manifest HD	44	15
HDClarity	LPMHD18	male	46.8	late pre-manifest HD	43	15
HDClarity	LPMHD19	male	47.4	late pre-manifest HD	41	18
HDClarity	HC1	female	48.2	healthy control	21	18
HDClarity	HC2	female	52.8	healthy control	24	22
HDClarity	HC3	male	62	healthy control	17	17
HDClarity	HC4	female	29.2	healthy control	22	14
HDClarity	HC5	male	54.6	healthy control	24	17
HDClarity	HC6	male	33.5	healthy control	24	17
HDClarity	HC7	male	71.9	healthy control	17	17
HDClarity	HC8	male	52	healthy control	17	9
HDClarity	HC9	female	43.2	healthy control	20	18
HDClarity	HC10	female	53.2	healthy control	22	19
HDClarity	HC11	male	53.7	healthy control	16	16

HDClarity	HC12	male	74.6	healthy control	17	15
HDClarity	HC13	female	58.4	healthy control	17	15
HDClarity	HC14	female	39.6	healthy control	18	9
HDClarity	HC15	male	26.5	healthy control	20	16
HDClarity	HC16	female	49	healthy control	18	17
HDClarity	HC17	female	28.9	healthy control	18	17
HDClarity	HC18	male	48.3	healthy control	15	15
HDClarity	HC19	male	50.6	healthy control	17	15
HDClarity	HC20	female	53.4	healthy control	12	10
HDClarity	HC21	male	54	healthy control	21	17
HDClarity	HC22	female	61	healthy control	24	20
HDClarity	HC23	male	35.4	healthy control	19	15
HDClarity	HC24	female	46.8	healthy control	17	17
HDClarity	HC25	male	52.2	healthy control	19	17
HDClarity	HC26	female	55.1	healthy control	22	19
HDClarity	HC27	male	57	healthy control	17	15
HDClarity	HC28	male	37.4	healthy control	24	19
HDClarity	HC29	female	44.7	healthy control	17	17
HDClarity	HC30	male	52.5	healthy control	22	15
HDClarity	HC31	female	53.1	healthy control	17	17
HDClarity	HC32	male	56.5	healthy control	18	17
U of W	EPMHD14	female	31	early pre-manifest HD	40	unknown
U of W	LPMHD20	female	34	late pre-manifest HD	50	unknown
U of W	LPMHD21	male	46	late pre-manifest HD	42	unknown
U of W	LPMHD22	male	55	late pre-manifest HD	42	unknown
U of W	LPMHD23	female	53	late pre-manifest HD	46	unknown
U of W	LPMHD24	female	38	late pre-manifest HD	48	unknown
U of W	LPMHD25	male	31	late pre-manifest HD	44	unknown
U of W	LPMHD26	male	35	late pre-manifest HD	35	unknown
U of W	LPMHD27	female	52	late pre-manifest HD	42	unknown
U of W	LPMHD28	male	42	late pre-manifest HD	42	unknown
U of W	HC33	female	59	Healthy control	unknown	unknown
U of W	HC34	female	21	Healthy control	unknown	unknown
U of W	HC35	female	20	Healthy control	unknown	unknown
U of W	HC36	female	51	Healthy control	unknown	unknown
U of W	HC37	female	57	Healthy control	unknown	unknown
U of W	HC38	female	60	Healthy control	unknown	unknown

U of W = University of Washington
EMHD = Early manifest HD
EPMHD = Early pre-manifest HD
LPHHD = Late pre-manifest HD
HC = Healthy control

Supplemental Table 2

Sample ID	Source	Sample prepar-ation	Sex	PMI	Age at death	Sample Type	CAG low/ high	Onset	COD	Brain Region	Vonsattel Grade
HC113	NZ Human Brain Bank	Fixed tissue	Male	14	58	HD	28/44	.	Broncho-pneumonia	Caudate Nucleus	2
HC114	NZ Human Brain Bank	Fixed tissue	Female	12	53	HD	21/47	.	Pneumonia	Caudate Nucleus	2
HC120	NZ Human Brain Bank	Fixed tissue	Male	15	51	HD	10/46	.	Pneumonia	Caudate Nucleus	2
HC126	NZ Human Brain Bank	Fixed tissue	Male	8	61	HD	17/43	.	Pneumonia	Caudate Nucleus	2
HC109	NZ Human Brain Bank	Fixed tissue	Female	7	59	HD	23/47	.	Bronchopneumonia	Caudate Nucleus	4
HC116	NZ Human Brain Bank	Fixed tissue	Male	8	54	HD	17/46	.	Pneumonia	Caudate Nucleus	4
HC122	NZ Human Brain Bank	Fixed tissue	Male	.	52	HD	10/50	.	Bowel obstruction	Caudate Nucleus	4
HC143	NZ Human Brain Bank	Fixed tissue	Female	16	45	HD	.	.	Respiratory arrest	Caudate Nucleus	4
H170	NZ Human Brain Bank	Fixed tissue	Male	17	60	Control	10/17	N/A	Ischemic heart disease	Caudate Nucleus	N/A
H189	NZ Human Brain Bank	Fixed tissue	Male	16	41	Control	18/22	N/A	Asphyxia	Caudate Nucleus	N/A
H165	NZ Human Brain Bank	Fixed tissue	Female	26	43	Control	17/17	N/A	Nitrogen poisoning	Caudate Nucleus	N/A
H238	NZ Human Brain Bank	Fixed tissue	Female	16	63	Control	14/16	N/A	Dissecting aortic aneurysm	Caudate Nucleus	N/A
H145	NZ Human Brain Bank	Fixed tissue	Male	6.5	54	Control	.	N/A	Ischemic heart disease	Caudate Nucleus	N/A
H140	NZ Human Brain Bank	Fixed tissue	Male	16	51	Control	.	N/A	Cardiomyopathy	Caudate Nucleus	N/A
B3470	Boston University	Fresh frozen tissue	Male	57.30	89	HD	40	70	.	Globus Pallidus	3
B3701	Boston University	Fresh frozen tissue	Female	14.30	67	HD	44	40	.	Globus Pallidus	3

B3703	Boston University	Fresh frozen tissue	Female	25.00	61	HD	45	35	.	Globus Pallidus	3
B4183	Boston University	Fresh frozen tissue	Female	20.30	76	HD	43	.	.	Globus Pallidus	4
B4230	Boston University	Fresh frozen tissue	Male	7.08	76	HD	41	58	.	Globus Pallidus	3
B4255	Boston University	Fresh frozen tissue	Female	17.50	52	HD	47	.	.	Globus Pallidus	4
5222	Human brain and spinal fluid resource center	Fresh frozen tissue	Male	.	61	Control	N/A	N/A	Micro-infarct (cerebrum)	Globus Pallidus	N/A
5214	Human brain and spinal fluid resource center	Fresh frozen tissue	Male	.	61	Control	N/A	N/A	.	Globus Pallidus	N/A
5293	Human brain and spinal fluid resource center	Fresh frozen tissue	Female	.	41	Control	N/A	N/A	Liver failure	Globus Pallidus	N/A
4308	Human brain and spinal fluid resource center	Fresh frozen tissue	Male	.	70	Control	N/A	N/A	Myocardial infarction	Globus Pallidus	N/A
5190	Human brain and spinal fluid resource center	Fresh frozen tissue	Male	.	68	Control	N/A	N/A	Myocardial infarction	Globus Pallidus	N/A
AN05954	Boston University	Fresh frozen tissue	Male	.	49	Pre-HD	.	N/A	.	Caudate and Putamen	N/A
AN16102	Boston University	Fresh frozen tissue	Female	.	86	Pre-HD	.	N/A	.	Caudate and Putamen	N/A

AN18592	Boston University	Fresh frozen tissue	Fem ale	.	82	Control	N/A	N/A	.	Caudate and Putame n	N/A
AN15392	Boston University	Fresh frozen tissue	Male	.	51	Control	N/A	N/A	.	Caudate and Putame n	N/A

PMI = post mortem interval
COD = cause of death
HD = Huntington's disease
N/A = not applicable
. = unkown

Supplemental Table 3

Figure	Panel	Genotype	Age	Brain region/Synaptic population	Treatment	No. of male mice	No. of female mice
2	b	WT	7 mo	Striatum	N/A	2	1
2	b	zQ175	7 mo	Striatum	N/A	2	1
2	c	WT	1 mo	Striatum	N/A	3	4
2	c	zQ175	1 mo	Striatum	N/A	3	4
2	c	WT	3 mo	Striatum	N/A	3	3
2	c	zQ175	3 mo	Striatum	N/A	3	3
2	c	WT	7 mo	Striatum	N/A	3	3
2	c	zQ175	7 mo	Striatum	N/A	3	3
2	d	WT	7 mo	Striatum	N/A	2	3
2	d	zQ175	7 mo	Striatum	N/A	2	3
2	e	WT	3 mo	Striatum	N/A	3	3
2	e	zQ175	3 mo	Striatum	N/A	3	3
2	f	WT	3 mo	Striatum	N/A	2	3
2	f	zQ175	3mo	Striatum	N/A	3	3
3	b	WT	7 mo	Striatum	N/A	2	2
3	b	zQ175	7 mo	Striatum	N/A	2	2
3	b	WT	7 mo	Motor cortex	N/A	1	2
3	b	zQ175	7 mo	Motor cortex	N/A	1	2
3	b	WT	7 mo	Hippocampus	N/A	1	2
3	b	zQ175	7 mo	Hippocampus	N/A	2	2
3	c	WT	3 mo	Striatum	N/A	2	3
3	c	zQ175	3 mo	Striatum	N/A	2	3
3	c	WT	7 mo	Striatum	N/A	2	2
3	c	zQ175	7 mo	Striatum	N/A	2	2
3	c	WT	12 mo	Striatum	N/A	1	2
3	c	zQ175	12 mo	Striatum	N/A	1	2
3	e	WT	7 mo	Striatum	N/A	2	3
3	e	zQ175	7 mo	Striatum	N/A	2	3
3	e	WT	7 mo	Motor cortex	N/A	2	2
3	e	zQ175	7 mo	Motor cortex	N/A	2	2
3	e	WT	7 mo	Hippocampus	N/A	2	2
3	e	zQ175	7 mo	Hippocampus	N/A	2	2
3	f	WT	3 mo	Striatum	N/A	2	3
3	f	zQ175	3 mo	Striatum	N/A	2	3
3	f	WT	7 mo	Striatum	N/A	2	3
3	f	zQ175	7 mo	Striatum	N/A	2	3
3	f	WT	12 mo	Striatum	N/A	2	2
3	f	zQ175	12 mo	Striatum	N/A	2	2
3	g	WT	3 mo	Corticostriatal	N/A	2	2
3	g	zQ175	3 mo	Corticostriatal	N/A	2	2
3	g	WT	3 mo	Thalamostriatal	N/A	2	2
3	g	zQ175	3 mo	Thalamostriatal	N/A	2	2
3	h	WT	3 mo	Striatum	N/A	2	2
3	h	zQ175	3 mo	Striatum	N/A	2	1
4	b	WT	4 mo	Striatum	N/A	2	2
4	b	zQ175	4 mo	Striatum	N/A	2	4
4	c	WT	4 mo	Striatum	N/A	2	3
4	c	zQ175	4 mo	Striatum	N/A	2	3
4	e	WT	12 mo	Striatum	N/A	2	2
4	e	BACHD	12 mo	Striatum	N/A	2	2
4	e	BR	12 mo	Striatum	N/A	2	2
4	e	BE	12 mo	Striatum	N/A	1	2
4	e	BER	12 mo	Striatum	N/A	2	2
4	f	WT	12 mo	Striatum	N/A	3	2
4	f	BACHD	12 mo	Striatum	N/A	1	2
4	f	BR	12 mo	Striatum	N/A	2	2
4	f	BE	12 mo	Striatum	N/A	1	2
4	f	BER	12 mo	Striatum	N/A	1	2
4	g	WT	12 mo	Striatum	N/A	3	2
4	g	BACHD	12 mo	Striatum	N/A	2	2
4	g	BR	12 mo	Striatum	N/A	2	2
4	g	BE	12 mo	Striatum	N/A	1	2
4	g	BER	12 mo	Striatum	N/A	1	2
5	b	zQ175	4 mo	Striatum	control IgG	2	3
5	b	zQ175	4 mo	Striatum	C1q function blocking antibody	2	3
5	c	zQ175	4 mo	Striatum	control IgG	3	4
5	c	zQ175	4 mo	Striatum	C1q function blocking antibody	3	3
5	e	WT	5mo	Striatum	control IgG	3	4
5	e	WT	5mo	Striatum	C1q function blocking antibody	3	4
5	e	zQ175	5mo	Striatum	control IgG	2	2
5	e	zQ175	5mo	Striatum	C1q function blocking antibody	3	4
5	f	WT	5mo	Striatum	control IgG	3	4
5	f	WT	5mo	Striatum	C1q function blocking antibody	3	4
5	f	zQ175	5mo	Striatum	control IgG	2	2
5	f	zQ175	5mo	Striatum	C1q function blocking antibody	3	4
5	h	zQ175	4 mo	Striatum	N/A	3	2
5	h	zQ175 CR3 KO	4 mo	Striatum	N/A	2	2
5	i	WT	4 mo	Striatum	N/A	16	12
5	i	zQ175	4 mo	Striatum	N/A	8	10
5	i	CR3 KO	4 mo	Striatum	N/A	13	11
5	i	zQ175 CR3 KO	4 mo	Striatum	N/A	9	4
5	i	WT	4 mo	Striatum	N/A	16	12
5	i	zQ175	4 mo	Striatum	N/A	8	10
5	i	CR3 KO	4 mo	Striatum	N/A	13	11
5	i	zQ175 CR3 KO	4 mo	Striatum	N/A	9	4

Supplemental Table 4

CR3 genotyping

Reagent setup

Reagent	μl per reaction
DNA	1
Water	2.5
CR3 Primer 1478 (5 μM)<br>TAGGCTATCCAGAGGTAGAC	0.5
CR3 Primer 1479 (5 μM)<br>CATACCTGTGACCAGAAGAGC	0.5
CR3 Primer 070 (5 μM)<br>ATCGCCTTCTTGACGAGTTC	0.5
Apex Red Taq DNA Master Mix, 2X	5

Thermocycling

Step	Temperature	Time/cycles
1	94°C	3 min
2	94°C	30 sec
3	58°C	1 min
4	72°C	1 min
5	Go to Step 2	35 cycles
6	72°C	2 min
7	10°C	Forever

Expected band sizes

CR3 KO 700bp

CR3 WT 350bp

Homer GFP genotyping

Reagent setup

Reagent	μl per reaction
DNA	1
Water	3
Homer GFP FW primer (5 μM)	0.5

CCTACGGCGTGCAGTGCTTCAGC	
Homer GFP Rev primer (5 μM)<br>CGGCGAGCTGCACGCTGCGTCCTC	0.5
Apex Red Taq DNA Master Mix, 2x	5

Thermocycling

**Step**	**Temperature**	**Time/cycles**
1	95°C	5 min
2	95°C	30 sec
3	50°C	30 sec
4	72°C	1 min
5	Go to Step 2	30 cycles
6	72°C	5 min
7	10°C	Forever

Expected band sizes

Homer-GFP 370 bp

KEY RESOURCES TABLE

REAGENT or RESOURCE	SOURCE	IDENTIFIER
Antibodies		
Homer1	Synaptic systems	Cat#160-003; RRID:AB _887730
VGLUT1	Millipore Sigma	Cat#AB5905; RRID:AB_2301751
VGLUT2	Millipore Sigma	Cat#AB2251, RRID:AB_2665454
PSD-95	Millipore Sigma	Cat#MAB1596, RRID:AB_2092365
Iba-1	Wako	Cat# 019-19741, RRID:AB_839504
Iba-1	Wako	Cat# ncnp24, RRID:AB_2811160
CD11b	Serotec	Cat# MCA711G; RRID:AB_321292
CD68	Serotec	Cat# MCA1957; RRID:AB_322219
CD68	Agilent Dako	Cat# M087629-2; RRID:AB_2074844
P2RY12	Anaspec	Cat# AS-55043A; RRID:AB_2298886
TMEM119	Abcam	Cat# ab209064; RRID:AB_2800343
C1q	Abcam	Cat# ab182451; RRID: AB_2732849
C1q	Agilent Dako	Cat# A0136; RRID: AB_2335698
C1q [JL-1]	Abcam	Cat# ab71940; RRID: AB_10711046
C3d	Agilent Dako	Cat# A006302; RRID: AB_578478
C3c	Agilent Dako	Cat# F0201; RRID: AB_2335709
iC3B	Quidel	Cat# A209; RRID: AB_452480
β-actin	Millipore Sigma	Cat#A2228; RRID:AB_476697
S100β	Agilent Dako	Cat#Z0311; RRID: AB_10013383
Secondary antibodies, Alexa Fluor conjugates various species	Thermo Fisher Scientific - Life Technologies	Cat#'s A-11073, A-11006, A-11012, A-21245; RRID's AB_2534117, AB_2534074, AB_141359, AB_141775
Digoxigenin	Roche	Cat#11207733910; RRIDAB_514500
Fluorescein	Roche	Cat#11426346910; RRIDAB_840257
Goat anti rabbit alkaline phosphatase	Abcam	Cat#ab97048; RRID:AB_10680574

Goat anti rabbit HRP	Promega	Cat#W4011; RRID:AB_430833
Peroxidase-AffiniPure Donkey anti-guinea pig IgG (H+L)	Jackson ImmunoResearch	Cat# 706-035-148 RRID:AB_2340447
Chemicals, Peptides, and Recombinant Proteins		
iC3B protein	Complement technologies	Cat# A115
C3c protein	Complement technologies	Cat# A116
C3 protein	Complement technologies	Cat# A113
C1q protein	Quidel	Cat# A400
Zymosan (preactivated)	Complement technologies	Cat# B400
C3/C4 inactivated serum	Complement technologies	Cat# A340
C3 depleted sera	Quidel	Cat# A508
C1q depleted sera	Quidel	Cat# A509
NaCl	Millipore Sigma	Cat# S9888
KCl	Millipore Sigma	Cat# P3911
$NaHCO_3$	Millipore Sigma	Cat# S6014
$CaCl_2$	Millipore Sigma	Cat# C1016
$MgCl_2$	Millipore Sigma	Cat# 208337
NaH_2PO_4	Millipore Sigma	Cat# S3139
Glucose	Millipore Sigma	Cat# G8270
Choline chloride	Millipore Sigma	Cat# C7017
Ascorbic acid	Millipore Sigma	Cat# 1043003
Pyruvic acid	Millipore Sigma	Cat# 107360
InVivoMab mouse IgG1 isotype control	Bio X Cell	Cat# BE0083; RRIDAB_1107784
Gabazine	Millipore Sigma	Cat# S106
$CsMeSO_3$	Millipore Sigma	Cat# C1426
Hepes	Millipore Sigma	Cat# 54457
EDTA	Millipore Sigma	Cat# 324626
MgATP	Millipore Sigma	Cat# A9187
QX-314	Millipore Sigma	Cat# 552233
Na-GTP	Millipore Sigma	Cat# 51120
Phosphocreatine	Millipore Sigma	Cat# P7936
CsOH	Millipore Sigma	Cat# 232068
16% paraformaldehyde	Electron microscope sciences	Cat# 15700
KH_2PO_4	Millipore Sigma	Cat# NIST200B
Sucrose	Millipore Sigma	Cat# S0389
Tissue-Tek O.C.T. Compound	Electron microscope sciences	Cat# 4583
Bovine serum albumin	Millipore Sigma	Cat# A2153
Triton-X 100	Millipore Sigma	Cat# T8787

Normal goat serum	Millipore Sigma	Cat# G9023
Vectashield with DAPI	Vector Laboratories	Cat# H-1000
2,2'-Thiodiethanol	Millipore Sigma	Cat# 166782
Dabco 33-LV	Millipore Sigma	Cat# 290734
Apex Red Taq DNA Master Mix, 2.0X	Genesee Scientific	Cat# 42-138
Power SYBR Green PCR Master Mix	Thermo Fisher Scientific	Cat# 4368708
1-Step PNPP Substrate solution	Thermo Fisher Scientific	Cat# 37621
ReBlot Plus Strong Antibody Stripping Solution 10x	EMD Millipore	Cat# 2504
Precision Plus Protein Kaleidoscope Standards	Bio-Rad	Cat# 161-0375
Super signal West Dura Substrate Trial Kit	Thermo Fisher Scientific	Cat# 37076
Glycine	Thermo Fisher Scientific - Fisher Bioreagents	Cat# BP381-1
ROCHE cOmplete protease inhibitor cocktail	Millipore Sigma	Cat# 4693116001
Sudan Black B	Millipore Sigma	Cat# 199664
Glycerol	Millipore Sigma	Cat# G5516
Hoechst	Thermo Fisher Scientific	Cat# H3570
LR white resin	Millipore Sigma	Cat# 62661
L-lysine	Millipore Sigma	Cat# L5501
Sodium azide	Millipore Sigma	Cat# 71289
Laemmli buffer	Bio-Rad	Cat# 161-0737
Sodium hydroxide	Millipore Sigma	Cat# 221465
Tris	Thermo Fisher Scientific	Cat# 15504020
SDS (sodium dodecyl sulphate)	Bio-Rad	Cat# 1610301
Proteinase K	Worthington Biochemical	Cat# LS004222
Phenol: Chloroform: Isoamyl Alcohol	Thermo Fisher Scientific	Cat# 15593031
Ethyl, alcohol, Pure	Millipore Sigma	Cat# E7023
Trizma base	Millipore Sigma	Cat# T1503
Methanol	Millipore Sigma	Cat# 179957
Triethanolamine	Millipore Sigma	Cat # T58300
Acetic anhydride	Millipore Sigma	Cat# AX0080-6
Hydrogen peroxide	Millipore Sigma	Cat# 95321
Experimental Models: Organisms/Strains		
Mouse: zQ175/ B6J.zQ175 KI	The Jackson Laboratory	Cat#027410; RRID:IMSR JAX:027410
Mouse: BACHD (and its variants BE/BR/BER[1])	The Jackson Laboratory	Cat#008197; RRID:IMSR JAX:008197
Mouse: CR3KO/ B6.129S4-Itgam^{tm1Myd} [2]	The Jackson Laboratory	Cat#003991; RRID:IMSR_JAX:003991
Mouse: Homer GFP[3]	From the laboratory of Professor Shigeo Okabe (Ebihara et al., 2003)	N/A

**Oligonucleotides**		
CR3 (Genotyping 1)<br>TAGGCTATCCAGAGGTAGAC	This paper	N/A
CR3 (Genotyping 2)<br>CATACCTGTGACCAGAAGAGC	This paper	N/A
CR3 (Genotyping 3)<br>ATCGCCTTCTTGACGAGTTC	This paper	N/A
Homer GFP (Genotyping 1)<br>CCTACGGCGTGCAGTGCTTCAGC	This paper	N/A
Homer GFP (Genotyping 2)<br>CGGCGAGCTGCACGCTGCGTCCTC	This paper	N/A
zQ175 hdh1 (Genotyping)<br>CATTCATTGCCTTGCTGCTAAG	Laragen	N/A
zQ175 hdh2 (Genotyping)<br>CTGAAACGACTTGAGCGACTC	Laragen	N/A
zQ175 Neo1 (Genotyping)<br>GATCGGCCATTGAACAAGATG	The Jackson Laboratory	
zQ175 Neo2 (Genotyping)<br>AGAGCAGCCGATTGTCTGTTG	The Jackson Laboratory	
GAPDH fw (RT-qPCR)<br>AGGTCGGTGTGAACGGATTTG	This paper	N/A
GAPDH rev (RT-qPCR)<br>TGTAGACCATGTAGTTGAGGTCA	This paper	N/A
P2RY12 mouse fw (RT-qPCR)<br>CACAGAGGGCTTTGGGAACTTA	This paper	N/A
P2RY12 mouse rev (RT-qPCR)<br>TGGTCCTGCTTCTGCTGAATC	This paper	N/A
Iba-1 mouse fw (RT-qPCR)<br>ATCAACAAGCAATTCCTCGATGA	This paper	N/A
Iba-1 mouse rev (RT-qPCR)<br>CAGCATTCGCTTCAAGGACATA	This paper	N/A
CR3 human fw (RT-qPCR)<br>GCCTTGACCTTATGTCATGGG	This paper	N/A
CR3 human rev (RT-qPCR)<br>CCTGTGCTGTAGTCGCACT	This paper	N/A
C3 human fw (RT-qPCR)<br>GGGGAGTCCCATGTACTCTATC	This paper	N/A
C3 human rev (RT-qPCR)<br>GGAAGTCGTGGACAGTAACAG	This paper	N/A
**Recombinant DNA**		
pCMV SPORT6 C3 (human)	Open Biosystems/Horizon	Cat# MHS6278-202800305; Clone Id# 40148812
pOTB7 C1QA (human)	Open Biosystems/Horizon	Cat# MHS6278-202832349; Clone Id# 4850418
pOTB7 ENO2 (human)	Open Biosystems/Horizon	Cat# MHS6278-202829522; Clone Id# 3629603
pCMV SPORT 6 C1qa (mouse)	Open Biosystems/Horizon	Cat# MMM1013-202704027; Clone Id# 3987436

pCMV-SPORT6 ENO2 (mouse)	Open Biosystems/Horizon	Cat# MMM1013-202703673; Clone Id# 3711960
pCMV-SPORT6 C3 (mouse)	Open Biosystems/Horizon	Cat# MMM1013-202768722; Clone Id# 5134713
pULTRA EGFP plasmid	Addgene/Horizon	Cat# 24129
RNAscope probes		
RNAscope® Probe- Mm-C3	ACD	Cat# 417841; Transcript target region 1821-2197
RNAscope® Probe- Mm-Acta2	ACD	Cat# 319531; Transcript target region 41-1749
RNAscope® 3-plex Positive Control Probe- Mm	ACD	Cat# 320881
RNAscope® 3-plex Negative Control Probe	ACD	Cat# 320871
Kits		
Alkaline phosphatase conjugation kit	Abcam	Cat# ab102850
FITC conjugation kit	Abcam	Cat# ab102884
TSA staining kit	Perkin Elmer	Cat# NEL0701001KT
SuperScript First-Strand Synthesis System for RT-PCR	Thermo Fisher Scientific	Cat# 11904018
Human Hemoglobin subunit alpha ELISA kit	Abcam	Cat# ab133999
Human Complement C3 ELISA kit (for CSF)	Abcam	Cat# ab108823
Human Complement C3 ELISA kit (for plasma)	Abcam	Cat# ab108822
Human Albumin ELISA kit	Abcam	Cat# ab108788
QuantiChrom Total Protein Assay kit	Fisher Scientific	Cat# QTPR-01K
BCA protein assay kit	Thermo Fisher Scientific	Cat# 23225
RNEasy Mini kit	Quiagen	Cat# 74104
RNAscope® Target Retrieval Reagents	ACD	Cat# 322000
RNAscope® Fluorescent Multiplex Reagent Kit	ACD	Cat# 320850
Viruses		
AAV1/2 h-syn-EGFP	Addgene	Cat# 50465: RRID: Addgene 50465

1. Wang, N.*, et al.* Neuronal targets for reducing mutant huntingtin expression to ameliorate disease in a mouse model of Huntington's disease. *Nat Med* **20**, 536-541 (2014).
2. Coxon, A.*, et al.* A novel role for the beta 2 integrin CD11b/CD18 in neutrophil apoptosis: a homeostatic mechanism in inflammation. *Immunity* **5**, 653-666 (1996).
3. Ebihara, T., Kawabata, I., Usui, S., Sobue, K. & Okabe, S. Synchronized formation and remodeling of postsynaptic densities: long-term visualization of hippocampal neurons expressing postsynaptic density proteins tagged with green fluorescent protein. *J Neurosci* **23**, 2170-2181 (2003).
