# Supplementary material for: Microglia and complement mediate early corticostriatal synapse loss and cognitive dysfunction in Huntington’s disease
Source: Nat Med. 2023 Oct 9;29(11):2866–84. doi: 10.1038/s41591-023-02566-3 (PMC10667107; doi:10.1038/s41591-023-02566-3)
Supplement: Supplementary file 2 — Reporting Summary [file 41591_2023_2566_MOESM2_ESM.pdf]

nature portfolio

Corresponding author(s): Beth Stevens

Last updated by author(s): Aug 22, 2023

Reporting Summary

Nature Portfolio wishes to improve the reproducibility of the work that we publish. This form provides structure for consistency and transparency in reporting. For further information on Nature Portfolio policies, see our Editorial Policies and the Editorial Policy Checklist.

Statistics

For all statistical analyses, confirm that the following items are present in the figure legend, table legend, main text, or Methods section.

n/a	Confirmed	
☐	☒	The exact sample size (*n*) for each experimental group/condition, given as a discrete number and unit of measurement
☐	☒	A statement on whether measurements were taken from distinct samples or whether the same sample was measured repeatedly
☐	☒	The statistical test(s) used AND whether they are one- or two-sided<br>*Only common tests should be described solely by name; describe more complex techniques in the Methods section.*
☐	☒	A description of all covariates tested
☐	☒	A description of any assumptions or corrections, such as tests of normality and adjustment for multiple comparisons
☐	☒	A full description of the statistical parameters including central tendency (e.g. means) or other basic estimates (e.g. regression coefficient) AND variation (e.g. standard deviation) or associated estimates of uncertainty (e.g. confidence intervals)
☐	☒	For null hypothesis testing, the test statistic (e.g. *F*, *t*, *r*) with confidence intervals, effect sizes, degrees of freedom and *P* value noted<br>*Give P values as exact values whenever suitable.*
☒	☐	For Bayesian analysis, information on the choice of priors and Markov chain Monte Carlo settings
☒	☐	For hierarchical and complex designs, identification of the appropriate level for tests and full reporting of outcomes
☐	☒	Estimates of effect sizes (e.g. Cohen's *d*, Pearson's *r*), indicating how they were calculated

Our web collection on statistics for biologists contains articles on many of the points above.

Software and code

Policy information about availability of computer code

Data collection	Electrophysiology - Data was collected with a Multiclamp 700B amplifier (Molecular Devices) and National Instruments acquisition board using custom ScanImage MATLAB software version 2017b (Mathworks; mbf bioscience).<br>Immunohistochemistry and in situ hybridisation - For confocal microscopy data was collected using Zen 2009 image acquisition software (Carl Zeiss), Zen Black 2.3 image acquisition software, Leica Application Suite X (LAS X) or Volocity image acquisition software (Perkin Elmer). For structured illumination microscopy data was collected using Zen Black 2012 image acquisition software (Carl Zeiss). Finally for array tomography analysis and cell culture staining data was collected using Axiovision software Rel. 4.8 (Carl Zeiss).<br>Quantitative RTPCR - For quantitative RTPCR analysis data was acquired using Q-Rex software (Qiagen).<br>Immunoblotting - ImageQuant LAS 4000 software was used for image acquisition.<br>ELISA assays - Absorbance readings were collected using EnSight software, Kaleido data acquisition and analysis version 1.2 (Perkin Elmer).<br>Operant touchscreen visual discrimination and cognitive flexibility assays - Measurements of trial completion, touchscreen interaction, and latency to collect reward were carried out using ABET II touch software running on a WhiskerServer Controller (Lafayette Instrument Company).<br>Open field assessment - Ambulatory activity and aspects of exploratory behavior were assessed using the Kinder Scientific Smart Frame Open Field System and Motor Monitor II software.
Data analysis	Electrophysiology - To identify the frequency and amplitude of sEPSCs, custom MATLAB scripts and analysis pipeline were adapted from Merel et al., J Neurosci Methods 269, 21-32 (2016).<br>Immunohistochemistry - Data analysis was performed using FIJI (Image J;NIH; Schindelin, J., Arganda-Carreras, I., Frise, E. et al. Fiji: an open-source platform for biological-image analysis. Nat. Methods 9, 676–682 (2012). https://doi.org/10.1038/nmeth.2019), Ilastik (Version 1.4; Berg, S., Kutra, D., Kroeger, T., Straehle, C.N., Kaisler, B.X., Haubold, C., Schiegg, M., Ales, J., Beier, T., Rudy, M., Eren, K., Cervantes, J.I., Xu, B.,

Beuttenmueller, F., Wolny, A., Zhang, C., Koethe, U., Hamprecht, F.A., Kreshuk, A., ilastik: interactive machine learning for (bio)image analysis. Nat. Methods 16, 1226-1232 (2019)), CellProfiler (Version 4.2.4; Lamprecht, M.R., Sabatini D.M., Carpenter, A.E., CellProfiler: free, versatile software for automated biological image analysis. Biotechniques 42, 71-75 (2007); www.cellprofiler.org) and Imaris 9.3 software (Oxford Instruments; Version 9.3.1, Bitplane, Zurich, Switzerland) with built in MATLAB plugins as detailed in the methods section.
In situ hybridization - Data analysis was performed using QuPath version 0.4.3
Quantitative RTPCR - Data analysis was performed using Q-Rex software (Quiagen) and Microsoft Excel (Microsoft version 16.16.3).
Immunoblotting - Data analysis was performed using FIJI (Image J;NIH software; Schindelin, J., Arganda-Carreras, I., Frise, E. et al. Fiji: an open-source platform for biological-image analysis. Nat Methods 9, 676–682 (2012). https://doi.org/10.1038/nmeth.2019).
ELISA assays - Data analysis was performed using Microsoft Excel (Microsoft version 16.16.3) and Prism 7 and 9 (GraphPad; versions 7e and 9.5.1) software.
Operant touchscreen visual disrimination and cognitive flexibility assays - Data analysis was performed using Microscoft Excel (Microscoft version 16.16.3) and Prism 9 software (GraphPad; version 9.5.1).
Open field assessment - Data analysis was performed using Microsoft Excel (Microsoft version 16.16.3) and Prism 9 (GraphPad; version 9.5.1) software.
Statistical analysis - Statistical analysis was performed using both Prism 7 and 9 (GraphPad; versions 7e and 9.5.1) and StataSE (Stata; Version 15.1).

For manuscripts utilizing custom algorithms or software that are central to the research but not yet described in published literature, software must be made available to editors and reviewers. We strongly encourage code deposition in a community repository (e.g. GitHub). See the Nature Portfolio guidelines for submitting code & software for further information.

Data

Policy information about availability of data

All manuscripts must include a data availability statement. This statement should provide the following information, where applicable:

- Accession codes, unique identifiers, or web links for publicly available datasets
- A description of any restrictions on data availability
- For clinical datasets or third party data, please ensure that the statement adheres to our policy

No information from previously published or publicly available datasets was employed in this study. All of the data supporting the findings of this study can be found within the article and its extended data and source data files. Extended data figure 1 has associated raw data for the immunoblots that can be located in Source data figures 11 and 12.
The biological repository identifiers for the CSF and plasma samples from the HDClarity cohort are restricted from distribution as a result of guidelines stipulated in the material transfer agreement. This was mandated by the foundation providing this material to ensure that IRB guidelines with regards to protection of participants personal information and identity are not disclosed. Further information about this as well as the procedures and application forms required to gain access to this information can be found at https://hdclarity.net/ and https://enroll-hd.org/. The timeframe from request to provision of data can take 1 to 2 mo depending on the IT and security infrastructure at your site.

Research involving human participants, their data, or biological material

Policy information about studies with human participants or human data. See also policy information about sex, gender (identity/presentation), and sexual orientation and race, ethnicity and racism.

Reporting on sex and gender

We have used the term sex in this manuscript to indicate a biological attribute as was suggested in the publishing guidelines provided by Nature medicine. Where data has been separated by sex and contrasts made on this basis this has been indicated in the figure panels, the figure legends and the results.
We considered sex in our study design and for the HDClarity cohort of CSF and plasma samples (Supplemental table 1) an approximately equal proportion of samples from male and female participants were collected and analyzed for each subject group (healthy controls, early premanifest HD, late premanifest HD, early manifest HD). This was also the case for the samples employed in the postmortem human tissue analysis (Figure 1, Extended data figure 1, Supplemental table 2) where similar numbers of HD and control samples were interrogated from males and females.
Information about the sex of the donors who provided CSF and plasma samples was collected by the clinicians who directly interacted with these patients. This was carried out in accordance with the laws and practices in place at their relevant medical institutions and according to the guidelines stipulated by the IRB protocols governing this research.
Where relevant in the source data and supplemental tables data is disaggregated on the basis of sex. Sex based analyses can be seen in Extended data a,b,c,d,e,f and sex was controlled for as a potentially confounding variable in figure 1d and e.

Reporting on race, ethnicity, or other socially relevant groupings

Information about the race or ethnicity of the individuals who donated CSF or plasma samples or those who consented to postmortem analysis of their brain tissue was unfortunately not provided to us and as such was not considered as a categorization variable.

Population characteristics

Relevant population characteristics of the human research participants include: age, sex, HD category i.e. early premanifest, late premanifest, early manifest, as defined by the HDClarity study protocol (see methods and https://hdclarity.net/) and CAG repeat number.

Recruitment

Recruitment of the patients who provided CSF and plasma for the HDClarity cohort of samples interrogated in this paper was carried out at clinical sites participating in the Enroll-HD program (http://www.enroll-hd.org) under the guidelines stipulated in the HDClarity study protocol (https://hdclarity.net/). Inclusion criteria for participants were as follows:

1. All eligible participants:
a. Are 21-75 years of age, inclusive; and
b. Are capable of providing informed consent or have a legal representative
authorized to give consent on behalf of the participant; and

c. Are capable of complying with study procedures, including fasting, blood sampling and lumbar puncture; and
d. Are participating in the Enroll-HD study; and
e. Will have had an Enroll-HD visit within two months of the Screening Visit.
2. For the Healthy Control group, participants eligible are persons who meet the following criteria:
a. Have no known family history of HD; or
b. Have known family history of HD but have been tested for the huntingtin gene CAG expansion and are not at genetic risk for HD (CAG < 36).
3. For the Early Pre-manifest HD group, participants eligible are persons who meet the following criteria:
a. Do not have clinical diagnostic motor features of HD, defined as Unified Huntington's Disease Rating Scale (UHDRS) Diagnostic Confidence Score < 4; and
b. Have CAG expansion ≥ 40; and
c. Have burden of pathology score, computed as (CAG – 35.5) × age, < 250.
4. For the Late Pre-manifest HD group, participants eligible are persons who meet the following criteria:
a. Do not have clinical diagnostic motor features of HD, defined as Unified Huntington's Disease Rating Scale (UHDRS) Diagnostic Confidence Score < 4; and
b. Have CAG expansion ≥ 40; and
c. Have burden of pathology score, computed as (CAG – 35.5) x age, ≥ 250.
5. For Early Manifest HD group, participants eligible are persons who meet the following criteria:
a. Have clinical diagnostic motor features of HD, defined as UHDRS Diagnostic Confidence Score = 4; and
b. Have CAG expansion ≥ 40; and
c. Have Stage I or Stage II HD, defined as UHDRS Total Functional Capacity (TFC) scores between 7 and 13 inclusive.

Exclusion criteria included:
1. For all groups, participants are ineligible if they meet any of the following exclusion criteria:
a. Use of investigational drugs or participation in a clinical drug trial within 30 days prior to Sampling Visit; or
b. Current intoxication, drug or alcohol abuse or dependence; or
c. If using any medications or nutraceuticals, the use of inappropriate (e.g., non-prescribed) or unstable dose within 30 days prior to Sampling Visit; or
d. Significant medical, neurological or psychiatric co-morbidity likely, in the judgment of the Site Principal Investigator, to impair participant's ability to complete study procedures, or likely to reduce the utility of the samples and data for the study of HD; or
e. Needle phobia, frequent headache, significant lower spinal deformity or major surgery; or
f. Antiplatelet or anticoagulant therapy within the 14 days prior to Sampling Visit, including but not limited to: aspirin, clopidogrel, dipyridamole, warfarin, dabigatran, rivaroxaban and apixaban; or
g. Clotting or bruising disorder; or
h. Screening blood test results more than 10% outside the lab's normal range for the following: white cell count, neutrophil count, lymphocyte count, hemoglobin (Hb), platelets, prothrombin time (PT) and activated partial thromboplastin time (APTT), or any combination of blood test results that the Site Principal Investigator deems to be of clinical significance; or
i. Screening blood test results for C-reactive protein (CRP)>2× upper limit of normal; or
j. Predictable non-compliance as assessed by Site Principal Investigator; or
k. Inability or unwillingness to undertake any of the study procedures; or
l. Exclusion during history or physical examination, final decision to be made by the Site Principal Investigator; including but not limited to:
i. any reason to suspect abnormal bleeding tendency, e.g. easy bruising, petechial rash; or
ii. any reason to suspect new focal neurological lesion, e.g. new headache, optic disc swelling, asymmetric focal long tract signs; or
iii. any other reason that, in the clinical judgment of the Site Principal Investigator, it is felt that lumbar puncture performed per this protocol and associated manuals is unsafe without brain imaging.
m. Serious Adverse Event (SAE) related to study procedures during or following any previous HDClarity Sampling Visit (if applicable), or SAE related to any other lumbar puncture in the previous 12 months.

The clinical collection sites are predominantly based in western Europe and the United States and thus the data will be reflective of the populations and ancestries of the peoples living in these regions and may not necessarily be replicated when interrogating samples from other areas of the world. With this exception the authors of this study are not aware of any other

biases that may have impacted the recruitment of sample donors.

Ethics oversight

A full list of the organizations that have approved the HDClarity study protocol has been provided below:
United States, Colorado
Rocky Mountain Movement Disorders Center Recruiting
Englewood, Colorado, United States
Contact: Karen Ortiz 303-867-5473 kortiz@kumarneuro.com
Principal Investigator: Rajeev Kumar, MD
United States, District of Columbia
Georgetown University Recruiting
Washington, District of Columbia, United States, 20057
Contact: Erin Koppel 202-687-1525 ek875@georgetown.edu
Principal Investigator: Karen Anderson, MD
United States, Maryland
John Hopkins University Recruiting
Baltimore, Maryland, United States, 21287
Contact: Kia Ultz 410-955-1349 kcarte23@jhmi.edu
Principal Investigator: Jee Bang, MPH, MD
United States, North Carolina
Wake Forest University Recruiting
Winston-Salem, North Carolina, United States, 27109
Contact: Christine O' Neill 336-716-8611 coneill@wakehealth.edu
Principal Investigator: Francis Walker
United States, Tennessee
Vanderbilt University Medical Center Recruiting
Nashville, Tennessee, United States, 37212
Contact: Elizabeth Huitz, RN 615-936-1007 elizabeth.huitz@vumc.org
Principal Investigator: Daniel Claassen, MD, MS
United States, Texas
University of Texas Health Science Center Recruiting
Houston, Texas, United States, 77030
Contact: Brittany Duncan 713-486-3134 brittany.j.duncan@uth.tmc.edu
Principal Investigator: Erin Furr-Stimming
Canada, British Columbia
University of British Columbia, The Centre for Huntingtons Disease Recruiting
Vancouver, British Columbia, Canada, V6T 2B5
Contact: Mike Adurogbangba 604-822-4872 madurogbangba@cmmt.ubc.ca
Principal Investigator: Blair Leavitt, MD, CM
Canada, Ontario
Centre for Movement Disorders Completed
Toronto, Ontario, Canada, M3B 2S7
Germany
University Hospital Ulm Recruiting
Ulm, Baden-Württemberg, Germany, 89081
Contact: Hela Jerbi +49 731-500-63080 hela.jerbi@uniklinik-ulm.de
Principal Investigator: Jan Lewerenz, MD
Dresden University Not yet recruiting
Dresden, Saxony, Germany, 01307
Contact: Simone Koegler 351- 458 2524 simone.koegler@uniklinikum-dresden.de
Principal Investigator: Bjoern Falkerburger, MD
St Josef And Elisabeth Hospital Recruiting
Bochum, Germany, 44791
Contact: Barbara Kaminski b.kaminski@klinikum-bochum.de
Principal Investigator: Carsten Saft
University Hospital of Erlangen Recruiting
Erlangen, Germany, 91054
Contact: Pia-Marie Pryssok 09131-85-44751 pia-marie.pryssok@uk-erlangen.de
Principal Investigator: Winkler Jurgen
George Huntington Institute Recruiting
Münster, Germany, 48149
Contact: Svenja Aufenberg +49-251-788-788-0 svenja.aufenberg@ghi-muenster.de
Principal Investigator: Ralf Reilmann
Italy
Fondazione I.R.C.C.S. Istituto Neurologico Carlo Besta Recruiting
Milan, Italy, 20133
Contact: Anna Castaldo (+)39 0223942519 anna.castaldo@istituto-besta.it
Principal Investigator: Caterina Mariotti, MD
Lega Italiana Ricera Huntington Recruiting
Rome, Italy, 00185
Contact: Consuelo Ceccarelli consuelo.ceccarelli@lirh.it
Principal Investigator: Ferdinando Squitieri, MD
Poland
Institute of Psychiatry and Neurology Recruiting
Warsaw, Poland, 02-957
Contact: Malgorzata Dusza-Rowińska +48 698250623 m.dusza.rowinska@gmail.com

Principal Investigator: Grzegorz Witkowski, MD, PhD
Spain
Hospital de Sant Pau Recruiting
Barcelona, Spain, 08041
Contact: Cristina Barrionuevo +34 649 14 23 60 CIzquierdo@santpau.cat
Principal Investigator: Jamie Kulisevsky, MD, PhD
United Kingdom
Royal Devon & Exeter NHS Foundation Trust Recruiting
Exeter, Devon, United Kingdom, EX2 5DW
Contact: Robert Wells 01392408181 Robert.wells2@nhs.net
Principal Investigator: Tim Harrower, MBBS
Glasgow Clinical Research Facility Recruiting
Glasgow, Scotland, United Kingdom, G51 4TF
Contact: Helen Bannister 0141 232 7600 helen.bannister@ggc.scot.nhs.uk
Principal Investigator: Matthew Sheridan, MBBS
Birmingham Huntingtons Disease Clinic Recruiting
Birmingham, West Midlands, United Kingdom, B15 2 FG
Contact: Jennifer De Souza 0121 301 2363 jennifer.desouza@bsmhft.nhs.uk
Principal Investigator: Hugh Rickards, MB, ChB, MSc, FRCPsych, MD
North Bristol NHS Trust Not yet recruiting
Bristol, United Kingdom, BS10 5NB
Contact: Catherine Watkins catherine.watkins@nbt.nhs.uk
Principal Investigator: Elizabeth Coulthard, MBBS
Cambridge University Hospitals NHS Foundation Trust Recruiting
Cambridge, United Kingdom, CB2 0PY
Contact: Katie Andresen 01223 331141 kera2@cam.ac.uk
Principal Investigator: Roger Barker
Cardiff University Recruiting
Cardiff, United Kingdom, CF24 4HQ
Contact: Alison Johnson 02920746394 alison.johnson@wales.nhs.uk
Principal Investigator: Anne Rosser, MD
Fife Health Board - Whyteman's Brae Hospital Recruiting
Kirkcaldy, United Kingdom, KY1 2ND
Contact: Fleur Davey 01383 623623 fleurdavey@nhs.net
Principal Investigator: Robert Thompson, MBBS
Leeds Teaching Hospital Trust Recruiting
Leeds, United Kingdom, LS7 4SA
Contact: Callum Schofield 0113 39 24679 callum.schofield@nhs.net
Principal Investigator: Jeremy Cosgrove, MBBS
The Walton Centre NHS Foundation Trust Recruiting
Liverpool, United Kingdom, L9 7LJ
Contact: Andrea Clyne, BSc MSc andrea.clyne@thewaltoncentre.nhs.uk
Principal Investigator: Rhys Davies, MA BM BCh PhD FRCP
University College London Hospitals NHS Foundation Trust Recruiting
London, United Kingdom, NW1 2PG
Contact: Fiona Kinsella 0203 108 2638 fiona.kinsella.19@ucl.ac.uk
Contact: Alexander Lowe alexander.lowe.16@ucl.ac.uk
Principal Investigator: Edward Wild, MD, PhD
St George's University Of London Recruiting
London, United Kingdom, SW17 0RE
Contact: Sally Goff 0208 725 5375 sally.goff@nihr.ac.uk
Principal Investigator: Nayana Lahiri
Oxford University Hospitals NHS Foundation Trust Recruiting
Oxford, United Kingdom, OX3 9DU
Contact: Zara Skitt 01865 234309 zara.skitt@ouh.nhs.uk
Principal Investigator: Richard Armstrong, MA, PhD, BMBCh, MRCP
University Hospitals Plymouth NHS Trust Recruiting
Plymouth, United Kingdom, PL6 5FP
Contact: Abigail Patrick 01752 439636 abigail.patrick1@nhs.net
Principal Investigator: Daniel Lashley, MBBS

Approval for the collection at the University of Washington was provided by the institutional review board at the University of Washington. The IRB approval number is 01174.

Note that full information on the approval of the study protocol must also be provided in the manuscript.

Field-specific reporting

Please select the one below that is the best fit for your research. If you are not sure, read the appropriate sections before making your selection.

☐ Life sciences ☐ Behavioural & social sciences ☐ Ecological, evolutionary & environmental sciences

For a reference copy of the document with all sections, see nature.com/documents/nr-reporting-summary-flat.pdf

Life sciences study design

All studies must disclose on these points even when the disclosure is negative.

Sample size	The sample size (n) of each experiment is provided in the corresponding main figure legend, extended data figure legend and/or methods section.<br>- Human postmortem tissue analysis: Figure 1 all; Sample sizes were chosen based on prior studies in which complement and microglial biology were assessed in human postmortem tissue in the context of neurodegenerative disease (Dejanovic et al., 2018; Wu et al., 2019).<br>- zQ175 and BACHD synaptic quantification studies: Fig. 2 a,b,c,d,e,f, Fig. 5 c,h, Extended data fig. 2, Extended data fig. 8 h and Extended data fig. 9 b,c; Sample sizes were chosen to support meaningful conclusions, based on prior studies assessing synaptic pathologies in these models using imaging and immunoblotting analysis (Indersmitten et al., 2015; Wang et al., 2014).<br>- zQ175 and BACHD complement deposition and colocalization analysis: Fig. 3 (all), Extended data fig. 3 (all), Extended data fig. 4 e,f,g,h Fig. 5 b and Extended data fig. 8 g and Extended data fig. 10 d,e; Group sizes were chosen based on prior studies which assessed complement biology in the brain using imaging strategies in the context of a range of different paradigms including pathological contexts (Hong et al., 2016; Schafer et al., 2016; Dejanovic et al., 2018; Lui et al., 2016; Werneburg et al., 2020).<br>- zQ175 complement transcript quantification: Extended data fig. 4 a,b,c,d; Group sizes were chosen based on prior studies which assessed levels of transcript associated with particular cell types (Hammond et al., 2019).<br>- zQ175 and BACHD assessments of microglial biology and engulfment: Fig. 4 (all), Extended data fig. 5,6,7 (all); Sample sizes were chosen based on in vivo quantifications of this parameter using comparable tools (Hong et al., 2016; Schafer et al., 2012; Lehrman et al., 2018).<br>- zQ175 electrophysiology studies: Fig. 5 d,e,f and Extended data fig. 8 i,j,k,l,m,n,o; Sample sizes were chosen based on previous studies which have observed differences in sEPSC frequency and amplitude as well as input resistance and capacitance in this and other HD model mice (Vezzoli et al., 2019; Indersmitten et al., 2015; Southwell et al., 2016; Goodliffe et al., 2018; Beaumont et al., 2016; Wood et al., 2008; Heikkinen et al., 2012).<br>- Operant touchscreen visual discrimination and cognitive flexibility assays and open field assessments of motor performance and exploratory behaviors: Fig. 5 i,j and Extended data fig. 9 d,e,f,g,h,i,j,k,l,m and Extended data fig. 10 a,b,c; Sample sizes were chosen based on previous studies in which the performance of zQ175 mice carrying out similar tasks was assessed. (Curtin et al., 2016; Piiponniemi et al., 2018; Menalled et al., 2012; Heikkinen et al., 2012).<br>- Human CSF and plasma analysis: Fig. 6 (all) and Extended data fig. 9,10 and 11 (all). To determine appropriate sample sizes for the CSF and plasma analysis a pilot study was performed using a small number of CSF and serum samples (sourced from the University of Washington). Based on the effect sizes observed a power analysis was performed using G*Power version 3.1.9.2 (Germany), which determined the number of individuals required to detect the same effect with 80% power at an alpha level of 2.5% (corrected for two primary comparisons using the Bonferroni method).
Data exclusions	No data was excluded.
Replication	Each experiment was repeated 2-3 times or more, with the total number of biological replicates indicated in the figure legends. All attempts at replication were successful.
Randomization	- For all studies zQ175 mice and their WT litter-mates at the age specified in the methods, results and figure legends, were randomly chosen (unless otherwise indicated in the methods section or figure legends this selection always comprised approximately equal numbers of male/female) from a large breeding colony comprised of multiple litters. An investigator who had not handled or inspected the mice for over 3 months or longer used a database in which the only characteristic noted was the age and the number of littermates present within the cage; there was no prior visual observation or metric such as weight used for the assignment. This was also the case for the zQ175 Homer, zQ175 CR3KO mice, BACHD, BR, BE and BER mice.<br>- For the studies involving treatment with the C1q blocking antibody or control IgG mice were randomly chosen (an investigator who had not handled or inspected the mice for over 3 months used a database in which the only characteristic noted was the age and the number of littermates present within the cage; there was no prior visual observation or metric such as weight used for the assignment) to receive a specific treatment but with a desire to ensure that mice from the same litter received both agents and that where possible mice with different genotypes in the same litter also received both agents.<br>- For studies involving the interrogation of plasma and CSF, samples were randomly assigned to a specific 96 well plate but distributed in such a way to ensure that the different patient patient subject groups were represented equally. In addition they were selected so that the gender balance and average age for each clinical group on that plate was kept equal and consistent with the average age of that clinical group as a whole.
Blinding	- For all studies methods for group allocation, data collection and all related analyses were predetermined. Blinding was applied to in vivo procedures and all data analysis (see methods).<br>- Electrophysiology: All mice were ear-tagged and subsequently given a coded id. The recordings were carried out by an investigator who was blinded to both genotype and treatment. Only after analysis was complete was the genotype and treatment information corresponding to the coded id provided by a separate investigator so that data could be assigned to the appropriate test group.<br>-Operant touchscreen and visual discrimination assays: All mice were ear-tagged and subsequently given a coded id. Mice were placed into the automated recording chambers by investigators who had no knowledge of their genotype and genotyping information was only revealed after the experiment was complete and data had been generated by the automated analysis pipeline in Abet II.<br>- For studies involving the interrogation of plasma and CSF, samples were pre-dispensed onto 96 well plates by one investigator. A different investigator who was blinded to the sample information subsequently performed the ELISA assays before a third investigator carried out the data analysis.

Behavioural & social sciences study design

All studies must disclose on these points even when the disclosure is negative.

Study description	*Briefly describe the study type including whether data are quantitative, qualitative, or mixed-methods (e.g. qualitative cross-sectional, quantitative experimental, mixed-methods case study).*
Research sample	*State the research sample (e.g. Harvard university undergraduates, villagers in rural India) and provide relevant demographic information (e.g. age, sex) and indicate whether the sample is representative. Provide a rationale for the study sample chosen. For studies involving existing datasets, please describe the dataset and source.*
Sampling strategy	*Describe the sampling procedure (e.g. random, snowball, stratified, convenience). Describe the statistical methods that were used to predetermine sample size OR if no sample-size calculation was performed, describe how sample sizes were chosen and provide a rationale for why these sample sizes are sufficient. For qualitative data, please indicate whether data saturation was considered, and what criteria were used to decide that no further sampling was needed.*
Data collection	*Provide details about the data collection procedure, including the instruments or devices used to record the data (e.g. pen and paper, computer, eye tracker, video or audio equipment) whether anyone was present besides the participant(s) and the researcher, and whether the researcher was blind to experimental condition and/or the study hypothesis during data collection.*
Timing	*Indicate the start and stop dates of data collection. If there is a gap between collection periods, state the dates for each sample cohort.*
Data exclusions	*If no data were excluded from the analyses, state so OR if data were excluded, provide the exact number of exclusions and the rationale behind them, indicating whether exclusion criteria were pre-established.*
Non-participation	*State how many participants dropped out/declined participation and the reason(s) given OR provide response rate OR state that no participants dropped out/declined participation.*
Randomization	*If participants were not allocated into experimental groups, state so OR describe how participants were allocated to groups, and if allocation was not random, describe how covariates were controlled.*

Ecological, evolutionary & environmental sciences study design

All studies must disclose on these points even when the disclosure is negative.

Study description	*Briefly describe the study. For quantitative data include treatment factors and interactions, design structure (e.g. factorial, nested, hierarchical), nature and number of experimental units and replicates.*
Research sample	*Describe the research sample (e.g. a group of tagged Passer domesticus, all Stenocereus thurberi within Organ Pipe Cactus National Monument), and provide a rationale for the sample choice. When relevant, describe the organism taxa, source, sex, age range and any manipulations. State what population the sample is meant to represent when applicable. For studies involving existing datasets, describe the data and its source.*
Sampling strategy	*Note the sampling procedure. Describe the statistical methods that were used to predetermine sample size OR if no sample-size calculation was performed, describe how sample sizes were chosen and provide a rationale for why these sample sizes are sufficient.*
Data collection	*Describe the data collection procedure, including who recorded the data and how.*
Timing and spatial scale	*Indicate the start and stop dates of data collection, noting the frequency and periodicity of sampling and providing a rationale for these choices. If there is a gap between collection periods, state the dates for each sample cohort. Specify the spatial scale from which the data are taken*
Data exclusions	*If no data were excluded from the analyses, state so OR if data were excluded, describe the exclusions and the rationale behind them, indicating whether exclusion criteria were pre-established.*
Reproducibility	*Describe the measures taken to verify the reproducibility of experimental findings. For each experiment, note whether any attempts to repeat the experiment failed OR state that all attempts to repeat the experiment were successful.*
Randomization	*Describe how samples/organisms/participants were allocated into groups. If allocation was not random, describe how covariates were controlled. If this is not relevant to your study, explain why.*
Blinding	*Describe the extent of blinding used during data acquisition and analysis. If blinding was not possible, describe why OR explain why blinding was not relevant to your study.*

Did the study involve field work? ☐ Yes ☐ No

Field work, collection and transport

Field conditions	*Describe the study conditions for field work, providing relevant parameters (e.g. temperature, rainfall).*
Location	*State the location of the sampling or experiment, providing relevant parameters (e.g. latitude and longitude, elevation, water depth).*
Access & import/export	*Describe the efforts you have made to access habitats and to collect and import/export your samples in a responsible manner and in compliance with local, national and international laws, noting any permits that were obtained (give the name of the issuing authority, the date of issue, and any identifying information).*
Disturbance	*Describe any disturbance caused by the study and how it was minimized.*

Reporting for specific materials, systems and methods

We require information from authors about some types of materials, experimental systems and methods used in many studies. Here, indicate whether each material, system or method listed is relevant to your study. If you are not sure if a list item applies to your research, read the appropriate section before selecting a response.

Materials & experimental systems

n/a	Involved in the study	
☐	☒	Antibodies
☐	☒	Eukaryotic cell lines
☒	☐	Palaeontology and archaeology
☐	☒	Animals and other organisms
☒	☐	Clinical data
☒	☐	Dual use research of concern
☒	☐	Plants

Methods

n/a	Involved in the study	
☒	☐	ChIP-seq
☒	☐	Flow cytometry
☒	☐	MRI-based neuroimaging

Antibodies

Antibodies used

Homer1 (Synaptic systems, Cat# 160003, RRID:AB _887730), VGLUT2 (Millipore Sigma, Cat# AB2251, RRID:AB_2665454), VGLUT1 (Millipore Sigma, Cat# AB5905, RRID:AB_2301751) Iba1 (Wako, Cat# 019-19741, RRID:AB_839504), Iba1 (Wako, Cat# ncnp24, RRID:AB_2811160), CD68 (Serotec, clone FA-11, Cat# MCA1957, RRID:AB_322219), CD68 (Dako, clone PG-M1, Cat# M087629-2, RRID:AB_2074844), CD11b (Serotec, clone 5C6, Cat# MCA711G, RRID:AB_321292), β -actin (Sigma, Cat# A2228, RRID:AB_476697), C1q (Abcam, clone 4.8, Cat# ab182451; RRID: AB_2732849), C1q (Dako, Cat# A0136, RRID: AB_2335698), C1q [JL-1] (Abcam, Cat # ab71940, RRID:AB_10711046), C3d (Dako, Cat# A0063, RRID:AB_578478), C3c (Dako, Cat# F0201, RRID:AB_2335709), iC3B (Quidel, Cat# A209, RRID:AB_452480) PSD-95 (Millipore, clone 6G6-1C9, Cat# MAB1596, RRID:AB_2092365), S100 beta (Dako, Cat# Z0311, RRID:AB_10013383), TMEM119 (Abcam, clone 28-3, Cat # ab209064, RRID:AB_2800343), P2RY12 (Anaspec, Cat # AS-55043A, RRID:AB_2298886) anti-flourescein-POD (Roche, Cat# 11 426 346 910, RRIDAB_840257), anti digioxigenin (Roche, Cat# 11207733910, RRID:AB_514500), alexa-flour conjugated secondary antibodies (Life Technologies, Cat#'s A-11073, Goat anti-Guinea Pig IgG (H+L) Highly Cross-Adsorbed Secondary Antibody, Alexa Fluor™ 488; A-11006, Goat anti-Rat IgG (H+L) Cross-Adsorbed Secondary Antibody, Alexa Fluor™ 488; A-11012, Goat anti-Rabbit IgG (H+L) Cross-Adsorbed Secondary Antibody, Alexa Fluor™ 594; A-21245, Goat anti-Rabbit IgG (H+L) Highly Cross-Adsorbed Secondary Antibody, Alexa Fluor™ 647; RRID's: AB_2534117, AB_2534074, AB_141359, AB_141775), Goat anti-Rabbit IgG H&L alkaline phosphatase (Abcam Cat# ab97048, RRID:AB_10680574) Goat anti rabbit HRP (Promega Cat# W4011, RRID:AB_430833), Goat anti mouse HRP (Promega Cat# W4021, RRID:AB_430834), Peroxidase-AffiniPure Donkey anti-guinea pig IgG (H+L) (Jackson ImmunoResearch Cat# 706-035-148, RRID:AB_2340447), C1q function blocking antibody (Annexon Biosciences ATCC accession number PTA-120399), control IgG (BioXCell, Cat# BE0083).

Validation

--Homer1 (Synaptic systems, Cat# 160003, RRID:AB _887730): Tests carried out by Synaptic Systems; assessment of specificity for Homer 1. Cross-reactivity of the serum to Homer 2 and 3 was removed by pre-adsorption with Homer 2 (aa 1 - 176) and Homer 3 (aa 1 - 177). Selected references: 1. Latrophilin-2 and latrophilin-3 are redundantly essential for parallel-fiber synapse function in cerebellum. Zhang RS, Liakath-Ali K, Südhof TCeLife (2020) 9; 2. Microglial activation arises after aggregation of phosphorylated-tau in a neuron-specific P301S tauopathy mouse model.van Olst L, Verhaege D, Franssen M, Kamermans A, Roucourt B, Carmans S, Ytebrouck E, van der Pol SMA, Wever D, Popovic M,Vandenbroucke RE, et al.Neurobiology of aging (2020) 89: 89-98; 3. Das MM, Godoy M, Chen S, Moser VA, Avalos P, Roxas KM, Dang I, Yáñez A, Zhang W, Bresee C, Arditi M, et al.Communications biology (2019) 2: 73.
--VGLUT2 (Millipore Sigma, Cat# AB2251, RRID:AB_2665454): Tests carried out by Millipore Sigma; evaluated by Western Blotting in rat brain membrane preparations. Selected references: 1. Lehrman, E.K., et al. CD47 Protects Synapses from Excess Microglia-Mediated Pruning during Development. Neuron 100, 120-134 e126 (2018). 2. Schafer, D.P., et al. 2. Microglia contribute to circuit defects in Mecp2 null mice independent of microglia-specific loss of Mecp2 expression. Elife 5(2016).
--VGLUT1 (Millipore Sigma, Cat# AB5905, RRID:AB_2301751): Tests carried out by Millipore Sigma; evaluated by Western Blotting in rat brain membrane preparations. Selected references: 1. Lehrman, E.K., et al. CD47 Protects Synapses from Excess Microglia-Mediated Pruning during Development. Neuron 100, 120-134 e126 (2018). 2. Schafer, D.P., et al., Microglia contribute to circuit defects in Mecp2 null mice independent of microglia-specific loss of Mecp2 expression. Elife 5(2016).
--Iba1 (Wako, Cat# 019-19741, RRID:AB_839504): Tests carried out by Fujifilm Wako show this antibody is reactive with human,

mouse and rat Iba-1. The manufacturers also state that this antibody is not suitable for immunoblotting applications. Selected references: 1. Ziehn, Marina O., Andrea A. Avedisian, Shannon M. Dervin, Elizabeth A. Umeda, Thomas J. O'Dell and Rhonda R. Voskuhl, "Therapeutic Testosterone Administration Preserves Excitatory Synaptic Transmission in the Hippocampus During Autoimmune Demyelinating Disease." Journal of Neuroscience, 32, no. 36 (September 5, 2012): 12312-24. 2. Lavisse, Sonia, Martine Guillermier, Anne-Sophie Herard, Fanny Petit, Marion Delahaye, Nadja Van Camp, Lucile Ben Haim, et al., "Reactive Astrocytes Overexpress TSPO and Are Detected by TSPO Positron Emission Tomography Imaging.", Journal of Neuroscience 32, no. 32, (August 8, 2012): 10809-18. 3. Shields, Shannon D., Xiaoyang Cheng, Nurcan Uceyler, Claudia Sommer, Sulayman D. Dib-Hajj, and Stephen G. Waxman. "Sodium Channel Nav1.7 Is Essential for Lowering Heat Pain Threshold After Burn Injury", Journal of Neuroscience, 32, no. 32 (August 8, 2012): 10819-32.
--Iba1 (Wako, Cat# ncnp24, RRID:AB_2811160): Tests carried out by Fujifilm Wako show that this antibody is reactive with human, mouse and marmoset Iba-1. The manufacturers have tested this antibody in IHC applications and provide representative images of Iba-1 positive cells in brain tissue sections of the listed species. Selected references: 1. Aran Groves, Yasuyuki Kiharam Deepa Jonnalagadda, Richard Rivera, Grace Kennedy, Mark Mayford, and Jerold Chun, "A functionally defined in vivo astrocyte population identified by c-Fos activation in a mouse model of multiple sclerosis modulated by S1P signaling: immediate-early astrocytes (ieAstrocytes)". eNeuro, (5) (September 24, 2018): 0239-18. 2. Jose Bruno N.F. Silva, Thayanne B. B. Calcia, Cyntia P. Sila, Rafael F. Guilherme, Fernando Almeida-Souza, Felipe S. Lemos, Katia S. Calabrese, Celso Caruso-Neves, Josiane S. Neves, and Claudia F. Benjamin. "ATRvD1 attenuates renal tubulointerstitial injury induced by albumin overload in sepsis-surviving mice". Int J Mol Sci. 22(21). (November 2021): 11634.
--CD68 (Serotec, clone FA-11, Cat# MCA1957, RRID:AB_322219): Tests carried out by Bio-Rad Serotec show that this antibody can be used in flow cytometry to detect intracellular mouse CD68 following permeabilization and can detect surface macrosialin at low levels in resident mouse peritoneal macrophages. Selected references: 1. Lehrman, E.K., et al. CD47 Protects Synapses from Excess Microglia-Mediated Pruning during Development. Neuron 100, 120-134 e126 (2018). 2. Schafer, D.P., et al. Microglia contribute to circuit defects in Mecp2 null mice independent of microglia-specific loss of Mecp2 expression. Elife 5(2016). 3. Hong, S., et al., Complement and microglia mediate early synapse loss in Alzheimer mouse models. Science 352, 712-716 (2016).
--CD68 (Dako, clone PG-M1, Cat# M087629-2, RRID:AB_2074844): Tests carried out by Agilent/Dako show that this antibody labels COS-1 and WOP cells transfected with CD68 cDNA. Unlike other CD68 antibodies, which label both macrophages and myeloid cells, tthis antibody detects a fixative-resistant epitope on the macrophage-restricted form of the CD68 antigen. Selected references: 1. Frafjord A, Skarshaug R, Hammarström C, et al. Antibody combinations for optimized staining of macrophages in human lung tumours. Scand J Immunol. 2020;92(1):e12889. doi:10.1111/sji.12889. 2. Carpino G, Nobili V, Renzi A, et al. Macrophage Activation in Pediatric Nonalcoholic Fatty Liver Disease (NAFLD) Correlates with Hepatic Progenitor Cell Response via Wnt3a Pathway. PLoS One. 2016;11(6):e0157246. Published 2016 Jun 16. doi:10.1371/journal.pone.0157246.
--CD11b (Serotec, clone 5C6, Cat# MCA711G, RRID:AB_321292): Tests carried out by Bio-Rad Serotec show that this antibody is reactive with mouse and human CD11b. Tests carried out by our laboratory; Evaluation of diminished signal in the cortex of zQ175 CR3 KO mice (This paper Extended data fig. 9a). Selected references: 1. Lehrman, E.K., et al. CD47 Protects Synapses from Excess Microglia-Mediated Pruning during Development. Neuron 100, 120-134 e126 (2018). 2. Schafer, D.P., et al. Microglia sculpt postnatal neural circuits in an activity and complement-dependent manner. Neuron 74, 691-705 (2012).
--β-actin (Sigma, Cat# A2228, RRID:AB_476697): Tested by the manufacturer in IHC and immunoblot paradigms using cell lines and extracts. Selected references: 1. Kanakkanthara A, Wilmes A, O'Brate A, et al. Peloruside- and laulimalide-resistant human ovarian carcinoma cells have βI-tubulin mutations and altered expression of βII- and βIII-tubulin isotypes. Mol Cancer Ther. 2011;10(8):1419-1429. doi:10.1158/1535-7163.MCT-10-1057. 2. Vandekerckhove J, Weber K. Actin amino-acid sequences. Comparison of actins from calf thymus, bovine brain, and SV40-transformed mouse 3T3 cells with rabbit skeletal muscle actin. Eur J Biochem. 1978;90(3):451-462. doi:10.1111/j.1432-1033.1978.tb12624.x 3. Drew JS, Moos C, Murphy RA. Localization of isoactins in isolated smooth muscle thin filaments by double gold immunolabeling. Am J Physiol. 1991;260(6 Pt 1):C1332-C1340. doi:10.1152/ajpcell.1991.260.6.C1332.
--C1q (Abcam, clone 4.8, Cat# ab182451; RRID: AB_2732849): Tests carried out by our laboratory and others; evaluation of diminished signal in C1qA KO mice relative to WT littermates by IHC (see www.abcam.com/c1q-antibody-48-ab182451.html#description_images_1). This antibody does not work in immunoblotting paradigms. Selected references: 1. Hong, S., et al., Complement and microglia mediate early synapse loss in Alzheimer mouse models. Science 352, 712-716 (2016). 2. Stephan AH, Madison DV, Mateos JM, et al. A dramatic increase of C1q protein in the CNS during normal aging. J Neurosci. 2013;33(33):13460-13474. doi:10.1523/JNEUROSCI.1333-13.2013.
--C1q (Dako, Cat# A0136, RRID: AB_2335698): Dako/Agilent claim that traces of contaminating antibodies have been removed by solid-phase absorption with human plasma proteins. Selected references: 1. Roumenina LT, Daugan MV, Noé R, et al. Tumor Cells Hijack Macrophage-Produced Complement C1q to Promote Tumor Growth. Cancer Immunol Res. 2019;7(7):1091-1105. doi:10.1158/2326-6066.CIR-18-0891. 2. Lubbers R, Oostindie SC, Dijkstra DJ, et al. Carbamylation reduces the capacity of IgG for hexamerization and complement activation. Clin Exp Immunol. 2020;200(1):1-11. doi:10.1111/cei.13411.
--C1q (Abcam, Cat # ab71940, RRID:AB_10711046): Tests carried out by Abcam show that this antibody recognizes mouse and human C1q. It has been tested in ELISA and dot blot applications. Selected references: 1. Datta D et al. Classical complement cascade initiating C1q protein within neurons in the aged rhesus macaque dorsolateral prefrontal cortex. J Neuroinflammation 17:8 (2020). 2. Bie B et al. Activation of mGluR1 Mediates C1q-Dependent Microglial Phagocytosis of Glutamatergic Synapses in Alzheimer's Rodent Models. Mol Neurobiol N/A:N/A (2019). 3. Zhu X et al. All-trans retinoic acid protects mesenchymal stem cells from immune thrombocytopenia by regulating the complement-IL-1ß loop. Haematologica N/A:N/A (2019).
--C3d (Dako, Cat# A0063, RRID:AB_578478): Tests carried out by Dako/Agilent using crossed immunoelectrophoresis show that only the C3d precipitation arch appears when using 12.5 ul A0063 per square cm gel area against 2 ul of complement activated human serum. No precipitate is seen when the antibody is tested against 10 ug of purified C3c. Tests carried out by our laboratory; evaluation of diminished signal in the livers of C3KO mice (data not shown), evaluation of capacity to detect enforced C3 expression in HEK 293 cells (this paper Extended data fig. 1n) and evaluation of diminished signal in the dorsal striatum of aged BAHCD C3KO mice (data not shown). Selected references: 1. Troldborg A, Jensen L, Deleuran B, Stengaard-Pedersen K, Thiel S, Jensenius JC. The C3dg Fragment of Complement Is Superior to Conventional C3 as a Diagnostic Biomarker in Systemic Lupus Erythematosus. Front Immunol. 2018;9:581. Published 2018 Mar 26. doi:10.3389/fimmu.2018.00581. 2. Liddelow SA, Guttenplan KA, Clarke LE, et al. Neurotoxic reactive astrocytes are induced by activated microglia. Nature. 2017;541(7638):481-487. doi:10.1038/nature21029.
--C3c (Dako, Cat# F0201, RRID:AB_2335709): Tests carried out by Dako/Agilent using crossed immunoelectrophoresis show that only reactivity with C3 complement and its C3c containing fragments is observed when using this antibody in a paradigm corresponding to 40ul of F0201 per square cm gel area against 2 ul of human plasma. In rocket electrophoresis, the antibody reacts with C3c complement from all 11 animal species tested so far: cat, cow, dog, guinea pig, horse, mink, mouse, rat, sheep and swine. Selected references: 1. Lipitsä T, Naukkarinen A, Laitala J, Harvima IT. Complement C3 is expressed by mast cells in cutaneous vasculitis and is degraded by chymase. Arch Dermatol Res. 2016;308(8):575-584. doi:10.1007/s00403-016-1677-0. 2. van den Hoogen P, de Jager

SCA, Huibers MMH, et al. Increased circulating IgG levels, myocardial immune cells and IgG deposits support a role for an immune response in pre- and end-stage heart failure. J Cell Mol Med. 2019;23(11):7505-7516. doi:10.1111/jcmm.14619.
--iC3b (Quidel, Cat# A209, RRID:AB_452480): Tests carried out by Quidel confirmed the specificity of the monoclonal antibody via a series of immunological techniques including ELISA, hemagglutination and RIA. The antibody was firstly shown by ELISA to bind to C3 antigens using highly pure, immobilized C3. Subsequent studies showed that this antibody agglutinates EC3bi but not EC3b or EC3d cells in an indirect hemagglutination assay. Further experiments showed that this antibody bound to radio-labeled purified iC3b but not to similarly labeled C3, C3b, C3d or C3. Tests carried out by our laboratory; when employed in a sandwich ELISA this antibody is selective for iC3b relative to full uncleaved C3 or a subsequent cleavage product C3c (This paper Extended data fig. 1m). It is also capable of detecting C3 activation induced by incubating serum at 4C or treating it with zymosan (This paper Extended data fig. 1k,l)." Selected references: 1. Tamerius, J.D., Pangburn, M., et al. Detection of a neoantigen on human iC3b and C3d by monoclonal Antibody, J. Immunol. 135:2015, 1985. 2. Rogers, J., Cooper, N., et al. Complement Activation by ß-amyloid in Alzheimer disease, PNAS 89:10016-10020, 1992.
--PSD-95 (Millipore, clone 6G6-1C9, Cat# MAB1596, RRID:AB_2092365): Tests carried out by Millipore have shown that this antibody recognizes bovine, mouse and rat PSD-95. The manufacturers have provided a representative IHC image on their website. Selected references: 1. STIM2 regulates PKA-dependent phosphorylation and trafficking of AMPARs. Garcia-Alvarez, G; Lu, B; Yap, KA; Wong, LC; Thevathasan, JV; Lim, L; Ji, F; Tan, KW; Mancuso, JJ; Tang, W; Poon, SY; Augustine, GJ; Fivaz, M. Molecular biology of the cell 26 1141-59 2015. 2. The X-linked mental retardation protein OPHN1 interacts with Homer1b/c to control spine endocytic zone positioning and expression of synaptic potentiation. Nakano-Kobayashi, A; Tai, Y; Nadif Kasri, N; Van Aelst, L. The Journal of neuroscience : the official journal of the Society for Neuroscience 34 8665-71 2014.
--S100 beta (Dako, Cat# Z0311, RRID:AB_10013383): Tests carried out by the manufacturer; "The antibody has been solid-phase absorbed with human plasma and cow serum proteins. In Western blotting of purified human recombinant S100 proteins, the antibody labels S100B strongly, S100A1 weakly, and S100A6 very weakly. No reaction was observed with the other S100 proteins tested, S100A2, S100A3 and S100A4. In indirect ELISA, the antibody shows no reaction with human plasma and cow serum. As demonstrated by IHC on formalin-fixed, paraffin-embedded tissue sections, the antibody cross-reacts with the S100 equivalent protein in man." Selected references: 1. Dun XP, Parkinson DB. Visualizing peripheral nerve regeneration by whole mount staining. PLoS One. 2015;10(3):e0119168. Published 2015 Mar 4. doi:10.1371/journal.pone.0119168. 2. Hastings RL, Mikesh M, Lee YI, Thompson WJ. Morphological remodeling during recovery of the neuromuscular junction from terminal Schwann cell ablation in adult mice. Sci Rep. 2020;10(1):11132. Published 2020 Jul 7. doi:10.1038/s41598-020-67630-1.
--TMEM119 (Abcam, clone 28-3, Cat # ab209064, RRID:AB_2800343): Tests carried out by the manufacturer; "this Tmem119 antibody has been knockout validated in IHC, meaning it demonstrates the a staining pattern consistent with microglial morphologies in wild type mouse brain sections and no or very little staining was observed in Tmem119 knockout mouse brain sections." Selected references 1. Hammond TR et al. Single-Cell RNA Sequencing of Microglia throughout the Mouse Lifespan and in the Injured Brain Reveals Complex Cell-State Changes. Immunity 50:253-271.e6 (2019). 2. Yousef H et al. Aged blood impairs hippocampal neural precursor activity and activates microglia via brain endothelial cell VCAM1. Nat Med 25:988-1000 (2019). 3. Bennett ML, Bennett FC, Liddelow SA, et al. New tools for studying microglia in the mouse and human CNS. Proc Natl Acad Sci U S A. 2016;113(12):E1738-E1746. doi:10.1073/pnas.1525528113.
--P2RY12 (Anaspec, Cat # AS-55043A, RRID:AB_2298886): The manufacturers established that this antibody yielded a band of approximately the correct molecular weight when probing STO whole cell lysate using western blotting. Selected references: 1. Bernier LP, Bohlen CJ, York EM, et al. Nanoscale Surveillance of the Brain by Microglia via cAMP-Regulated Filopodia. Cell Rep. 2019;27(10):2895-2908.e4. doi:10.1016/j.celrep.2019.05.010. 2. Peng, J., Liu, Y., Umpierre, A.D. et al. Microglial P2Y12 receptor regulates ventral hippocampal CA1 neuronal excitability and innate fear in mice. Mol Brain 12, 71 (2019). https://doi.org/10.1186/s13041-019-0492-x.
--anti flourescein-POD (Roche, Cat# 11 426 346 910, RRIDAB_840257): Selected references: 1. Vasek, M., Garber, C., Dorsey, D. et al. A complement–microglial axis drives synapse loss during virus-induced memory impairment. Nature 534, 538–543 (2016). https://doi.org/10.1038/nature18283. 2. Liddelow, S., Guttenplan, K., Clarke, L. et al. Neurotoxic reactive astrocytes are induced by activated microglia. Nature 541, 481–487 (2017). https://doi.org/10.1038/nature21029.
--anti digioxigenin (Roche, Cat# 11207733910, RRID:AB_514500): Selected references: 1. Vasek, M., Garber, C., Dorsey, D. et al. A complement–microglial axis drives synapse loss during virus-induced memory impairment. Nature 534, 538–543 (2016). https://doi.org/10.1038/nature18283. 2. Liddelow, S., Guttenplan, K., Clarke, L. et al. Neurotoxic reactive astrocytes are induced by activated microglia. Nature 541, 481–487 (2017). https://doi.org/10.1038/nature21029.
--alexa-flour conjugated secondary antibodies (Life Technologies, Cat#'s A-11073, Goat anti-Guinea Pig IgG (H+L) Highly Cross-Adsorbed Secondary Antibody, Alexa Fluor™ 488; A-11006, Goat anti-Rat IgG (H+L) Cross-Adsorbed Secondary Antibody, Alexa Fluor™ 488; A-11012, Goat anti-Rabbit IgG (H+L) Cross-Adsorbed Secondary Antibody, Alexa Fluor™ 594; A-21245, Goat anti-Rabbit IgG (H+L) Highly Cross-Adsorbed Secondary Antibody, Alexa Fluor™ 647; RRID's: AB_2534117, AB_2534074, AB_141359, AB_141775): Tests carried out by the manufacturer; "To minimize cross-reactivity, antibodies have been pre cross-adsorbed against bovine IgG, goat IgG, rabbit IgG, rat IgG, human IgG, and human serum. Cross-adsorption or pre-adsorption is a purification step to increase specificity of the antibody resulting in less background staining and cross-reactivity. The secondary antibody solution is passed through a column matrix containing immobilized serum proteins from potentially cross-reactive species. Only the nonspecific-binding secondary antibodies are captured in the column, and the highly specific secondaries flow through. Further passages through additional columns result in highly cross-adsorbed preparations of secondary antibody." Selected references: 1. Gonzalez-Perez O, López-Virgen V, Ibarra-Castaneda N. Permanent Whisker Removal Reduces the Density of c-Fos+ Cells and the Expression of Calbindin Protein, Disrupts Hippocampal Neurogenesis and Affects Spatial-Memory-Related Tasks. Front Cell Neurosci. 2018;12:132. Published 2018 May 15. doi:10.3389/fncel.2018.00132. 2. Hahn JM, Combs KA, Lloyd CM, McFarland KL, Boyce ST, Supp DM. Identification of Merkel cells associated with neurons in engineered skin substitutes after grafting to full thickness wounds. PLoS One. 2019;14(3):e0213325. Published 2019 Mar 5. doi:10.1371/journal.pone.0213325.
--goat anti-rabbit IgG H&L alkaline phosphatase (Abcam Cat# ab97048, RRID:AB_10680574): Tests carried out by the manufacturer; "By immunoelectrophoresis and ELISA this antibody reacts specifically with rabbit IgG and with light chains common to other rabbit immunoglobulins. No antibody was detected against non- immunoglobulin serum proteins. This antibody may cross react with IgG from other species." Selected references: 1. Jacques LC et al. Increased pathogenicity of pneumococcal serotype 1 is driven by rapid autolysis and release of pneumolysin. Nat Commun 11:1892 (2020). 2. Mondal P et al. Mixture effect of arsenic and fluoride at environmentally relevant concentrations in zebrafish (Danio rerio) liver: Expression pattern of Nrf2 and related xenobiotic metabolizing enzymes. Aquat Toxicol 213:105219 (2019).
--Goat anti rabbit HRP (Promega Cat# W4011, RRID:AB_430833): Selected references: 1. Hu, D. et al. (2012) Novel insight into KLF4 proteolytic regulation in estrogen receptor signaling and breast carcinogenesis. J. Biol. Chem. 287, 13584–97. 2. Hesse, E. et al. (2010) Zfp521 controls bone mass by HDAC3-dependent attenuation of Runx2 activity. J. Cell Biol. 191, 1271–83.
--Goat anti mouse HRP (Promega Cat# W4021, RRID:AB_430834): Selected references: Hu, D. et al. (2012) Novel insight into KLF4

proteolytic regulation in estrogen receptor signaling and breast carcinogenesis. J. Biol. Chem. 287, 13584–97. 2. Hesse, E. et al. (2010) Zfp521 controls bone mass by HDAC3-dependent attenuation of Runx2 activity. J. Cell Biol. 191, 1271–83.
--Peroxidase-AffiniPure Donkey anti-guinea pig IgG (H+L) (Jackson ImmunoResearch Cat# 706-035-148, RRID:AB_2340447): Tests carried out by the manufacturer; "Based on immunoelectrophoresis and/or ELISA, the antibody reacts with whole molecule guinea pig IgG. It also reacts with the light chains of other guinea pig immunoglobulins. No antibody was detected against non-immunoglobulin serum proteins. The antibody has been tested by ELISA and/or solid-phase adsorbed to ensure minimal cross-reaction with bovine, chicken, goat, syrian hamster, horse, human, mouse, rabbit, rat and sheep serum proteins, but it may cross-react with immunoglobulins from other species." Selected references: 1. Leidal AM, Huang HH, Marsh T, et al. The LC3-conjugation machinery specifies the loading of RNA-binding proteins into extracellular vesicles. Nat Cell Biol. 2020;22(2):187-199. doi:10.1038/s41556-019-0450-y. 2. Zhang XM, François U, Silm K, et al. A proline-rich motif on VGLUT1 reduces synaptic vesicle super-pool and spontaneous release frequency. Elife. 2019;8:e50401. Published 2019 Oct 30. doi:10.7554/eLife.50401.
--C1q function blocking antibody (Annexon Biosciences ATCC accession number PTA-120399): Tests carried out by our laboratory; Following 1mo of treatment with the C1q function blocking antibody the concentration of free unbound C1q in mouse serum was significantly reduced relative to that seen in mice treated with control IgG (this paper Extended data fig. 8d). Tests carried out by the manufacturer; In in vitro paradigms the antibody was found to bind to C1q from multiple species. It was also capable of blocking red blood cell hemolysis induced by classical complement pathway activation. Selected references: 1. Lansita JA, Mease KM, Qiu H, Yednock T, Sankaranarayanan S, Kramer S. Nonclinical Development of ANX005: A Humanized Anti-C1q Antibody for Treatment of Autoimmune and Neurodegenerative Diseases. Int J Toxicol. 2017;36(6):449-462. doi:10.1177/1091581817740873 2. Hong S, Beja-Glasser VF, Nfonoyim BM, et al. Complement and microglia mediate early synapse loss in Alzheimer mouse models. Science. 2016;352(6286):712-716. doi:10.1126/science.aad8373. 2. Vukojicic A, Delestrée N, Fletcher EV, et al. The Classical Complement Pathway Mediates Microglia-Dependent Remodeling of Spinal Motor Circuits during Development and in SMA. Cell Rep. 2019;29(10):3087-3100.e7. doi:10.1016/j.celrep.2019.11.013.
--Control IgG (BioXCell, Cat# BE0083): Tests carried out by the manufacturer; >95% purity as assessed by SDS-PAGE. Selected references: 1. Faraco, G., et al. (2018). “Dietary salt promotes neurovascular and cognitive dysfunction through a gut-initiated TH17 response.” Nat Neurosci 21(2): 240-249. 2. Macal, M., et al. (2018). “Self-Renewal and Toll-like Receptor Signaling Sustain Exhausted Plasmacytoid Dendritic Cells during Chronic Viral Infection.” Immunity 48(4): 730-744 e735.

Eukaryotic cell lines

Policy information about cell lines and Sex and Gender in Research

Cell line source(s)	- HEK293 cells for transduction with the pULTRA EGFP and pULTRA EGFP T2A C3Ms constructs were obtained from ATCC ref CRL-1573
Authentication	This cell line was not authenticated.
Mycoplasma contamination	This cell line was not tested for mycoplasma contamination.
Commonly misidentified lines (See ICLAC register)	There are no commonly misidentified cell lines used in this study.

Animals and other research organisms

Policy information about studies involving animals; ARRIVE guidelines recommended for reporting animal research, and Sex and Gender in Research

Laboratory animals	--zQ175 mice (JAX stock number 027410) and their WT littermates, which are on a C57BL/6J congenic background, were either provided directly by CHDI or were bred in house. In all experiments both male and female mice were used at the ages indicated in the figure legends, results and methods (these include 1,2,3,4,7 and 12 mo of age).<br>--BACHD (JAX stock number 008197),BR,BE,BER mice and their WT littermates (Wang et al., 2014 and Gray et al., 2008), which are on an FVB/NJ congenic background, were obtained from The Jackson Laboratory or the laboratory of William Yang at UCLA. In all experiments both male and female mice were used at the ages indicated in the figure legends, results and methods (these include 7 and 13 mo of age).<br>--CR3KO mice (JAX stock number 027410) were bred in house and crossed to zQ175 mice to generate zQ175 heterozygous CR3KO mice. These mice are on a C57BL/6J congenic background. In all experiments both male and female mice were used at the ages indicated in the figure legends, results and methods (these include 1 and 4 mo of age).<br>--Homer GFP mice were obtained from the laboratory of Shiego Okabe at Tokyo Medical and Dental University and bred in house. These mice are on a C57BL/6J congenic background. In all experiments both male and female mice were used at the ages indicated in the figure legends, results and methods (these include 4 and 7 mo of age).
Wild animals	No wild animals were used in this study.
Reporting on sex	An approximately equal number of male and female mice were utilized for all of the experiments carried out in this study (the exact breakdown is stipulated in supplemental table 3) and as such we can confirm that the findings of this study do not apply to only one sex. Sex was considered in the study design in the following way: to ensure that the findings were not applicable to only one sex a mixed population of male and female mice was employed in all experiments. Data is only provided disaggregated for sex in Extended data figure 8 e and f where weight changes in response to treatment with the C1q blocking antibody or control IgG were being evaluated. This was provided for clarity due to the well established disparity in the weight of male and female mice. In all other instances separating data generated from male and female mice would have made sample sizes to small to draw any meaningful insights or conclusions.

Field-collected samples	No field-collected samples were used in this study.
Ethics oversight	All studies were performed at either Boston Children's Hospital or UCLA and were approved by the institutional care and use committee of Boston Children’s Hospital and UCLA in accordance with National Insitutes of Health (NIH) guidelines for the humane treatment of animals.

Note that full information on the approval of the study protocol must also be provided in the manuscript.
