# Supplementary material for: Microglia and complement mediate early corticostriatal synapse loss and cognitive dysfunction in Huntington’s disease
Source: Nat Med. 2023 Oct 9;29(11):2866–84. doi: 10.1038/s41591-023-02566-3 (PMC10667107; doi:10.1038/s41591-023-02566-3)
Supplement: Supplementary file 14 — Source data for graphs and statistical analysis associated with Extended Data Fig. 2 and full-length, unprocessed blots associated with the immunoblot data presented in Extended Data Fig. 2c,g,l. [file 41591_2023_2566_MOESM14_ESM.pdf]

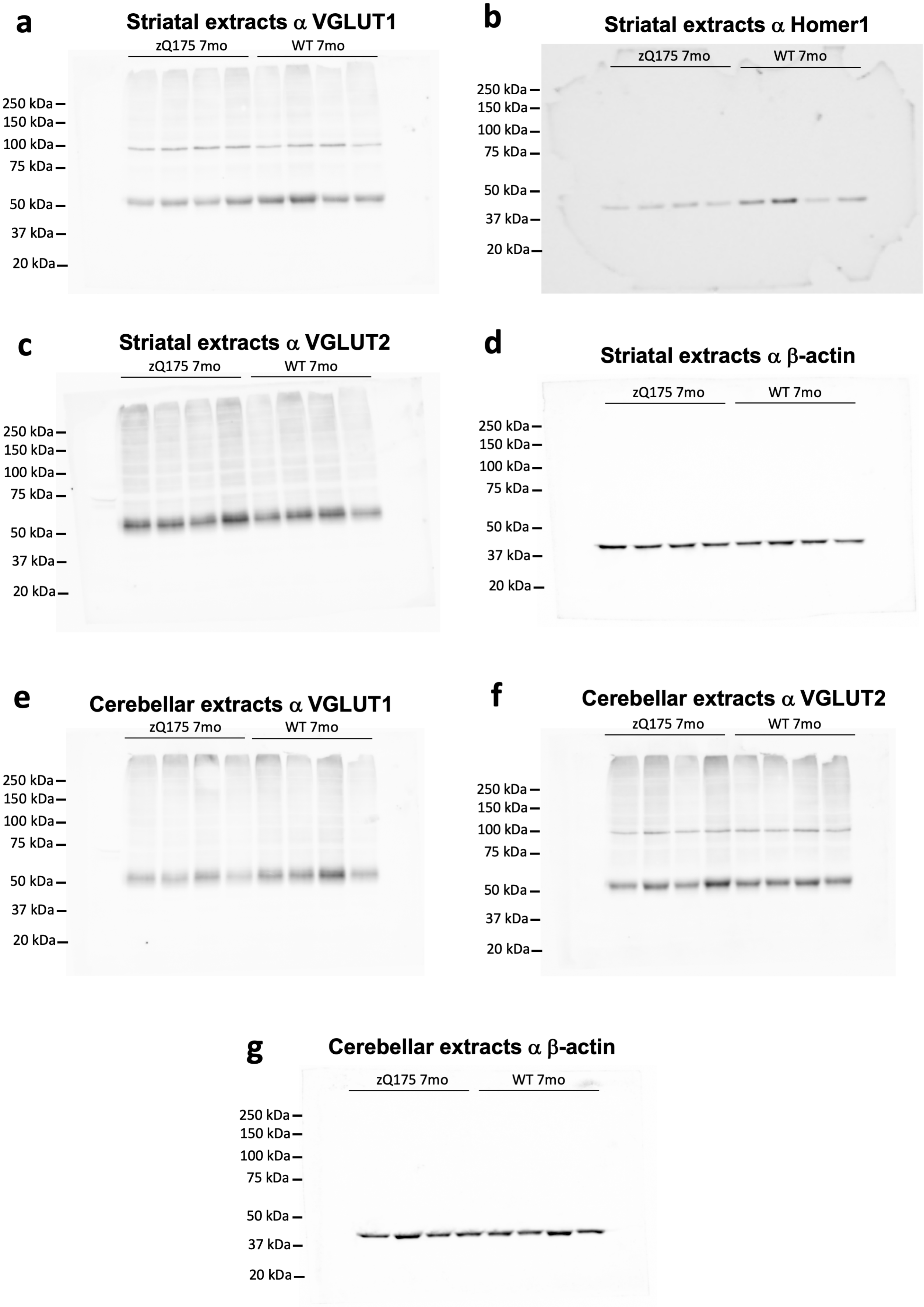
a
Striatal extracts α VGLUT1
zQ175 7mo
WT 7mo
250 kDa
150 kDa
100 kDa
75 kDa
50 kDa
37 kDa
20 kDa
b
Striatal extracts α Homer1
zQ175 7mo
WT 7mo
250 kDa
150 kDa
100 kDa
75 kDa
50 kDa
37 kDa
20 kDa
c
Striatal extracts α VGLUT2
zQ175 7mo
WT 7mo
250 kDa
150 kDa
100 kDa
75 kDa
50 kDa
37 kDa
20 kDa
d
Striatal extracts α β-actin
zQ175 7mo
WT 7mo
250 kDa
150 kDa
100 kDa
75 kDa
50 kDa
37 kDa
20 kDa
e
Cerebellar extracts α VGLUT1
zQ175 7mo
WT 7mo
250 kDa
150 kDa
100 kDa
75 kDa
50 kDa
37 kDa
20 kDa
f
Cerebellar extracts α VGLUT2
zQ175 7mo
WT 7mo
250 kDa
150 kDa
100 kDa
75 kDa
50 kDa
37 kDa
20 kDa
g
Cerebellar extracts α β-actin
zQ175 7mo
WT 7mo
250 kDa
150 kDa
100 kDa
75 kDa
50 kDa
37 kDa
20 kDa

Source data figure 11 (a) Full length lanes of the chemiluminescent anti VGLUT1 (~55kDa) signal displayed in Extended data figure 1c; approximate molecular weights, estimated with Precision Plus Protein Kaleidoscope prestained protein standards (BIO-RAD), are shown to the left. **(b)** Full length lanes of the chemiluminescent anti Homer1 (~39kDa) signal displayed in Extended data figure 1c; approximate molecular weights, estimated with Precision Plus Protein Kaleidoscope prestained protein standards (BIO-RAD), are shown to the left. **(c)** Full length lanes of the chemiluminescent anti VGLUT2 (~56kDa) signal displayed in Extended data figure 1c; approximate molecular weights, estimated with Precision Plus Protein Kaleidoscope prestained protein standards (BIO-RAD), are shown to the left. **(d)** Full length lanes of the chemiluminescent anti β-actin (~42kDa) signal displayed in Extended data figure 1c; approximate molecular weights, estimated with Precision Plus Protein Kaleidoscope prestained protein standards (BIO-RAD), are shown to the left. **(e)** Full length lanes of the chemiluminescent anti VGLUT1 (~55kDa) signal displayed in Extended data figure 1g; approximate molecular weights, estimated with Precision Plus Protein Kaleidoscope prestained protein standards (BIO-RAD), are shown to the left. **(f)** Full length lanes of the chemiluminescent anti VGLUT2 (~56kDa) signal displayed in Extended data figure 1g; approximate molecular weights, estimated with Precision Plus Protein Kaleidoscope prestained protein standards (BIO-RAD), are shown to the left. **(g)** Full length lanes of the chemiluminescent anti β-actin (~42kDa) signal displayed in Extended data figure 1g; approximate molecular weights, estimated with Precision Plus Protein Kaleidoscope prestained protein standards (BIO-RAD), are shown to the left.

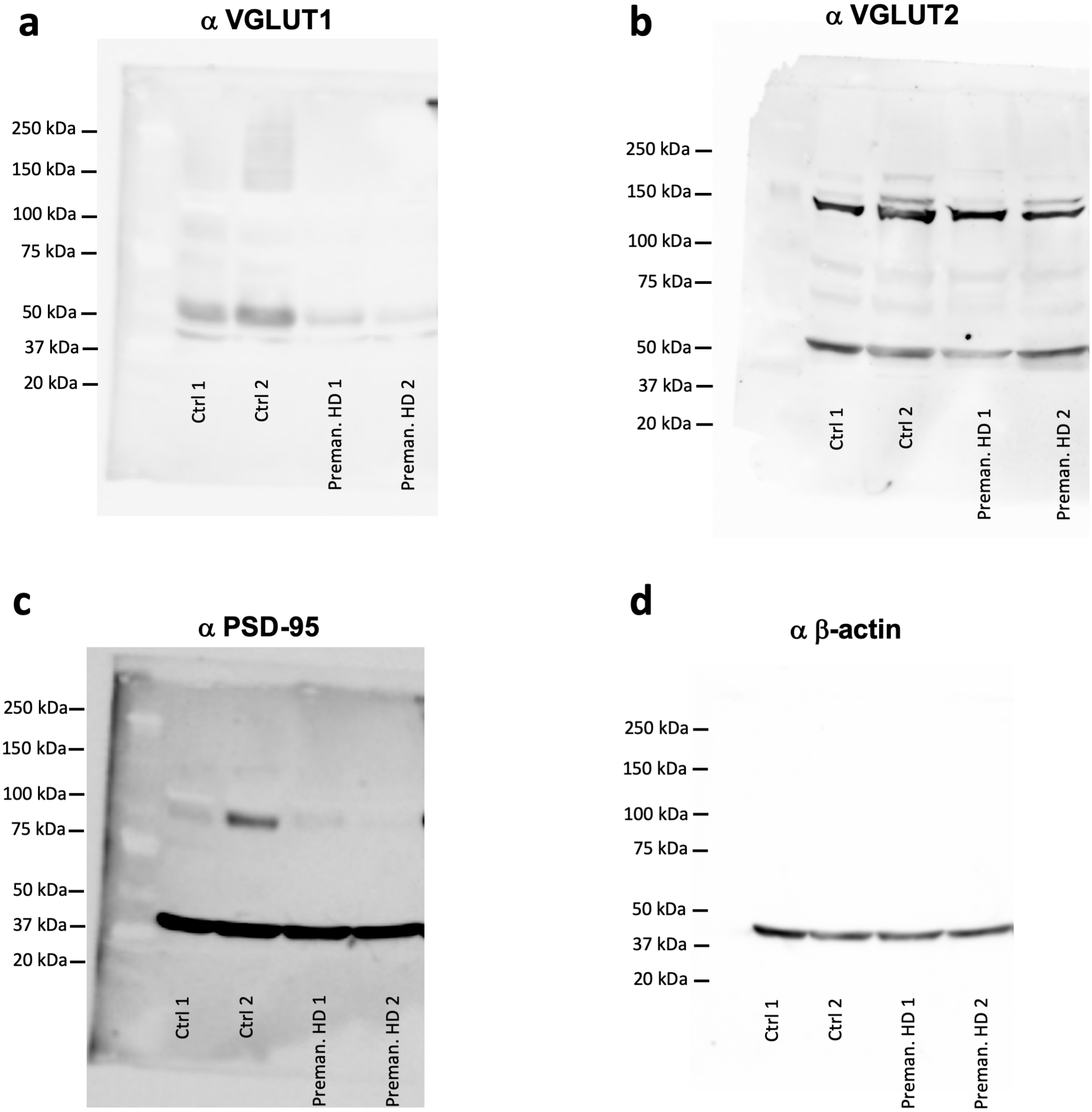
a
α VGLUT1
250 kDa
150 kDa
100 kDa
75 kDa
50 kDa
37 kDa
20 kDa
Ctrl 1
Ctrl 2
Preman. HD 1
Preman. HD 2
b
α VGLUT2
250 kDa
150 kDa
100 kDa
75 kDa
50 kDa
37 kDa
20 kDa
Ctrl 1
Ctrl 2
Preman. HD 1
Preman. HD 2
c
α PSD-95
250 kDa
150 kDa
100 kDa
75 kDa
50 kDa
37 kDa
20 kDa
Ctrl 1
Ctrl 2
Preman. HD 1
Preman. HD 2
d
α β-actin
250 kDa
150 kDa
100 kDa
75 kDa
50 kDa
37 kDa
20 kDa
Ctrl 1
Ctrl 2
Preman. HD 1
Preman. HD 2

Source data figure 12 (a) Full length lanes of the chemiluminescent anti VGLUT1 (~55kDa) signal displayed in Extended data figure 1l; approximate molecular weights, estimated with Precision Plus Protein Kaleidoscope prestained protein standards (BIO-RAD), are shown to the left. **(b)** Full length lanes of the chemiluminescent anti VGLUT2 (~56kDa) signal displayed in Extended data figure 1l; approximate molecular weights, estimated with Precision Plus Protein Kaleidoscope prestained protein standards (BIO-RAD), are shown to the left. **(c)** Full length lanes of the chemiluminescent anti PSD-95 (~95kDa) signal displayed in Extended data figure 1l; approximate molecular weights, estimated with Precision Plus Protein Kaleidoscope prestained protein standards (BIO-RAD), are shown to the left. **(d)** Full length lanes of the chemiluminescent anti β-actin (~42kDa) signal displayed in Extended data figure 1l; approximate molecular weights, estimated with Precision Plus Protein Kaleidoscope prestained protein standards (BIO-RAD), are shown to the left. Please note that for all blots additional lanes to the right of Preman.HD 2, which contained extracts from a different source and which were run to address questions for an alternate project, have been cropped for clarity. Abbreviations: Preman. = Premanifest. Ctrl = Control
